# Increased Expression of α‐Synuclein Impairs Trafficking of the Essential SNARE Protein Synaptobrevin‐2

**DOI:** 10.1111/jnc.70568

**Published:** 2026-10-08

**Authors:** Elyas H. Arvell, James P. Carroll, Joshua Saito, Holly Melland, Ranjith R. Unnithan, Chris French, Danny M. Hatters, Sarah L. Gordon

**Affiliations:** ^1^ The Florey Institute of Neuroscience and Mental Health The University of Melbourne Parkville Victoria Australia; ^2^ Drug Discovery Biology, Monash Institute of Pharmaceutical Sciences, Faculty of Pharmacy and Pharmaceutical Sciences Monash University Parkville Victoria Australia; ^3^ Neuromedicines Discovery Centre Monash University Parkville Victoria Australia; ^4^ Department of Electrical and Electronic Engineering The University of Melbourne Parkville Victoria Australia; ^5^ Department of Medicine The University of Melbourne Parkville Victoria Australia; ^6^ Department of Biochemistry and Pharmacology The University of Melbourne Parkville Victoria Australia

**Keywords:** α‐synuclein, endocytosis, neurotransmission, Parkinson's disease, protein trafficking, synaptobrevin‐2

## Abstract

α‐Synuclein is a presynaptic protein that has been extensively studied for its role in Parkinson's disease. Multiplication and missense variants in the gene encoding α‐synuclein are causes of familial PD, and aggregated α‐synuclein is a major component of Lewy bodies and Lewy neurites, the histopathological hallmark of the disease. However, the native function of α‐synuclein remains unclear, though several lines of evidence suggest it modulates presynaptic function. α‐Synuclein binds the vesicular SNARE protein synaptobrevin‐2/VAMP2, and increased levels of α‐synuclein potentiate SNARE complex levels without impacting exocytic rate. Given the essential role of synaptobrevin‐2 in synaptic function we sought to examine how increased expression of α‐synuclein, at levels observed in familial synucleinopathies with *SNCA* triplication, impacts the trafficking of this essential SNARE protein. Modest (approximately 2‐fold) overexpression of α‐synuclein in cultured murine hippocampal neurons caused a slowing in the endocytic retrieval of synaptobrevin‐2, without impacting either the targeting of the protein to synapses or altering endocytosis. We also show that α‐synuclein is not working antagonistically with the synaptobrevin‐2 chaperone synaptophysin, and likely acts at least partially via direct binding to synaptobrevin‐2. Finally, increased expression of α‐synuclein induces synaptic depression upon repeated neuronal depolarisation, in a manner requiring binding to synaptobrevin‐2. We reveal α‐synuclein as a modulator of synaptobrevin‐2 endocytic trafficking and highlight perturbed SNARE protein trafficking as a potential contributor to synaptic dysfunction and neuronal health in Parkinson's disease.

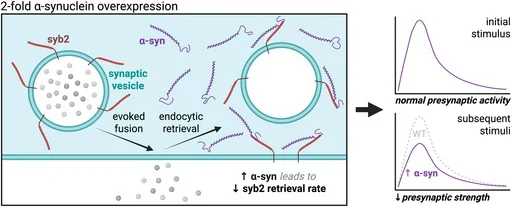

## Graphical abstract

α‐Synuclein is a presynaptic protein extensively studied for its role in Parkinson's disease; however, its native function remains unclear. Here, we show that modest (approximately 2‐fold) overexpression of α‐synuclein in cultured neurons, at levels observed in familial synucleinopathies with *SNCA* triplication, impairs the endocytic retrieval of the vesicular SNARE protein synaptobrevin‐2, at least partially via direct binding to synaptobrevin‐2. This results in synaptic depression upon repeated neuronal depolarisation. We reveal α‐synuclein as a modulator of synaptobrevin‐2 endocytic trafficking and highlight perturbed SNARE protein trafficking as a potential contributor to synaptic dysfunction in Parkinson's disease.

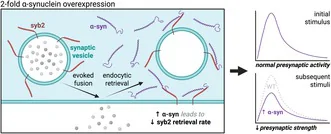

**Abbreviations**
CNQX: 6‐cyano‐7‐nitroquinoxaline‐2,3‐dione
CV: coefficient of variation
DL‐AP5: DL‐2‐amino‐5‐phosphonopentanoic acid
MEM: minimal essential medium
PD: Parkinson's disease
PLA: proximity ligation assay
ROIs: regions of interest
SNARE: soluble N‐ethylmaleimide‐sensitive factor attachment protein receptors
syb2: synaptobrevin‐2
syt1: synaptotagmin‐1
VAMP2: vesicle‐associated membrane protein (also known as synaptobrevin‐2)
WT: wild‐type
α‐syn^AAA^
: KKD > AAA mutant α‐synuclein
α‐syn‐GFP: α‐synuclein‐GFP

## 1 Introduction

Parkinson's disease (PD) is the second most common neurodegenerative disorder, with global prevalence estimated to exceed 8.5 million (Ou et al. 2021). 5%–10% of PD cases are familial, but the majority of cases are idiopathic with unknown aetiology (Balestrino and Schapira 2020). α‐Synuclein is implicated in the pathophysiology of both genetic and idiopathic forms of the disorder; aggregated α‐synuclein is a major component of the hallmark PD pathology of Lewy bodies and Lewy neurites, and point mutations, duplications and triplications in the *SNCA* gene encoding this protein contribute to the development of familial PD (Trinh et al. 2018). Copy number variations in *SNCA* have been demonstrated to have a dose‐dependent relationship with α‐synuclein expression, wherein *SNCA* triplication results in a 2‐fold increase in neuronal α‐synuclein (Farrer et al. 2004; Miller et al. 2004). Overexpression of α‐synuclein is thought to potentiate the aggregation of the protein (Nuber et al. 2013), which is theorised to contribute to PD pathogenesis, and has been the focus of intense scrutiny. However, an area that is much less studied is how changes to the cellular load of α‐synuclein impacts synaptic function and neuronal health. This is relevant, as while it has long been hypothesised that synaptic dysfunction may underly early pathogenesis of PD (reviewed by Gcwensa et al. (2021) and Picconi et al. (2012)), the molecular mechanisms behind this dysfunction are still poorly understood.

α‐Synuclein is a neuronal protein widely expressed throughout the brain and highly enriched at nerve terminals (Jakes et al. 1994; Maroteaux et al. 1988; Iwai et al. 1995). There is strong evidence that within presynaptic boutons, α‐synuclein acts as a regulator of the synaptic vesicle cycle (reviewed by Gao et al. (2023) and Sharma and Burre (2023)), the process that controls the fusion (exocytosis) and retrieval (endocytosis) of neurotransmitter‐containing synaptic vesicles to enable high‐fidelity and sustained neurotransmission. Increased expression of α‐synuclein has been shown to influence presynaptic dynamics in complex and sometimes contradictory ways. The recycling pool of vesicles was reduced in hippocampal neuronal cultures overexpressing α‐synuclein (Nemani et al. 2010; Scott and Roy 2012), and replenishment of the readily releasable pool of vesicles was reduced in hippocampal slices from mice overexpressing α‐synuclein (Gureviciene et al. 2007). Increased expression of α‐synuclein has been shown to either enhance (Janezic et al. 2013) or impair (Scott et al. 2010; Song et al. 2023) synaptic vesicle clustering within presynaptic terminals. Overexpression of α‐synuclein has also been demonstrated to slow synaptic vesicle fusion kinetics (Scott et al. 2010; Krenn et al. 2020), and impair both exocytosis (DeWitt and Rhoades 2013; Darios et al. 2010) and endocytosis (Scott et al. 2010; Song et al. 2023).

Consequently, it is unsurprising that increased expression of α‐synuclein can alter neurotransmission. Overexpressing α‐synuclein in hippocampal neuronal cultures led to reduced neuronal excitability, action potential frequency and network activity (Tolö et al. 2018), and in hippocampal slices decreased field EPSP frequency (Nemani et al. 2010). Increased expression of α‐synuclein also reduced the number of active presynaptic terminals over time in neuronal cultures (Scott et al. 2010; Leite et al. 2022). 5‐HT neurotransmission was impaired in transgenic mice overexpressing α‐synuclein in serotonergic neurons, inducing a depressive phenotype (Miquel‐Rio et al. 2022). Firing was also dampened in dopaminergic neurons of the substantia nigra in slices from transgenic mice overexpressing α‐synuclein, although dopamine content was increased in the caudate putamen of the striatum (Janezic et al. 2013). In contrast, increased firing rate and alterations to firing patterns have been observed in dopaminergic neuronal cultures overexpressing α‐synuclein, as well as heightened intra‐ and extracellular dopamine levels (Dagra et al. 2021).

The molecular mechanisms by which α‐synuclein mediates presynaptic activity are still poorly understood. The dynamic C‐terminal tail of α‐synuclein, rich in acidic and proline residues, can bind to the N‐terminus of the vesicular SNARE (Soluble N‐ethylmaleimide‐Sensitive Factor Attachment Protein Receptors) protein synaptobrevin‐2, also known as VAMP2 (Burre et al. 2012; Diao et al. 2013; Atias et al. 2019). Synaptobrevin‐2 assembles into ternary *trans* SNARE complexes with the plasma membrane SNARE proteins syntaxin‐1 and SNAP‐25 that drive vesicle fusion and thus neurotransmitter release (Jahn et al. 2023). Increased levels of α‐synuclein potentiate SNARE complex levels; however, this does not impact exocytic rate (Burre et al. 2010), suggesting that the increased SNARE complex levels could reflect changes to SNARE protein dynamics.

We have recently demonstrated that activity‐dependent phosphorylation of α‐synuclein mediates the post‐stimulus trafficking of synaptobrevin‐2 (Arvell et al. 2026). Given the essential role of synaptobrevin‐2 in synaptic function, we sought to examine how increased expression of α‐synuclein, at levels observed in PD patients with *SNCA* triplication, affects the trafficking of this essential vesicular SNARE protein and, consequently, synaptic function. By exogenously introducing human α‐synuclein into primary cultured neurons, we reveal that modest (approximately 2‐fold) overexpression of α‐synuclein impairs synaptobrevin‐2 retrieval following stimulation, without impacting endocytosis per se, and that upon repeated stimulation this leads to synaptic depression. The impacts were observed in the presence of wild‐type α‐synuclein, but not an α‐synuclein mutant with reduced affinity for synaptobrevin‐2, suggesting that a direct interaction between α‐synuclein and synaptobrevin‐2 underlies these effects.

## 2 Methods

### 2.1 Materials

Synaptobrevin‐2‐pHluorin (syb2‐pHluorin) and synaptotagmin‐1‐pHluorin (syt1‐pHluorin) were supplied by Prof. G. Miesenböck (Oxford University, England) and Prof. V. Haucke (Leibniz Institute of Molecular Pharmacology, Berlin, Germany), respectively. mCerulean, synaptophysin‐mCerulean, synaptobrevin‐2‐flag (syb2‐flag) and mCherry were supplied by Prof. M. Cousin (Centre for Discovery Brain Sciences, University of Edinburgh, Scotland), human α‐synuclein in pcDNA3.1+ vector was from Roberto Cappai (University of Melbourne, Australia), and human α‐synuclein‐GFP (αsyn‐GFP) was from Jason Howitt (Swinburne University, Australia). QuikChange II Site‐Directed Mutagenesis kit was from Agilent Technologies (Cat# 200523, Santa Clara, CA, USA). Thermo Plasmid Maxiprep Kit (Cat# K210017), Neurobasal media (Cat# 21103049), B‐27 supplement (Cat# 17504044), penicillin/streptomycin (Cat# 15140122), L‐glutamine (Cat# 25030081), Minimal Essential Medium (MEM) (Cat# 11095080), Dulbecco's MEM/F‐12 (Cat# 11320033), Lipofectamine 2000 (Cat# 11668019), goat anti‐chicken Alexa Fluor 488 (Cat# A11039, RRID:AB_2534096), goat anti‐chicken DyLight 550 (Cat# SA5‐10071, RRID:AB_2556651), donkey anti‐mouse DyLight 550 (Cat# SA5‐10167, RRID:AB_2556747), and donkey anti‐rabbit Alexa Fluor 647 (Cat# A31573, RRID:AB_2536183) antibodies were obtained from Thermo Fisher Scientific (Scoresby, VIC, Australia). The mouse anti‐α‐synuclein antibody (Cat# 610787, RRID:AB_398108) was from BD Biosciences (Franklin Lakes, NJ, USA), and the chicken anti‐GFP (Cat# ab13970, RRID:AB_300798) and rabbit anti‐syb2 (Cat# ab3347, RRID:AB_2212462) antibodies were obtained from Abcam (Cambridge, UK). The rabbit anti‐syt1 antibody (Cat# 105102, RRID:AB_887835) was obtained from Synaptic Systems (Göttingen, Germany). Dako Fluorescence Mounting Medium (Cat# S302380‐2) was obtained from Agilent Technologies (Santa Clara, CA, USA). Papain (Cat# LK003178) was obtained from Worthington Biochemical Corporation (Lakewood, NJ, USA). 6‐cyano‐7‐nitroquinoxaline‐2,3‐dione (CNQX) was from ENZO Life Sciences (Cat#: A16110, Lausen, Switzerland). DL‐2‐Amino‐5‐phosphonopentanoic acid (DL‐AP5) (Cat#: 14540) was from Cayman Chemical (Ann Arbor, MI, USA). All other reagents were obtained from Sigma‐Aldrich (Castle Hill, Australia).

### 2.2 Plasmid Preparation

Site‐directed mutagenesis was used to mutate residues 96–98 in human α‐synuclein or human α‐synuclein‐GFP from KKD to AAA (α‐synuclein^AAA^) in two steps. Mutagenesis was performed using the QuikChange II site‐directed mutagenesis kit as per the manufacturer's instructions. Mutagenic primers for step 1 were CTTTGTCGCAGCAGACCAGTTGGGCAAG (forward) and CTTGCCCAACTGGTCTGCTGCGACAAAG (reverse), and for step 2 were CTTTGTCGCAGCAGCACAGTTGGGCAAG (forward) and CTTGCCCAACTGTGCTGCTGCGACAAAG (reverse). All mutations were confirmed by Sanger sequencing (Australian Genome Research Facility, Melbourne, Australia). Transfection‐grade DNA was prepared using the Thermo Plasmid Maxiprep Kit as per the manufacturer's directions.

### 2.3 Hippocampal Primary Neuronal Cultures

All procedures were approved by the Florey Animal Ethics Committee and performed in accordance with the guidelines of the National Health and Medical Research Council Code of Practice for the Care and Use of Animals for Experimental Purposes in Australia (approval number FINMH18045, FINMH22024 and 2023‐051). Wild‐type C57BL/6J mice (RRID:IMSR_JAX:000664) were obtained from Ozgene ARC and maintained as an in‐house colony, and B6;129X1‐*Snca* ^*tm1Rosl*^/J α‐synuclein knockout (KO) mice (Abeliovich et al. 2000) (RRID:IMSR_JAX:003692) were obtained from The Jackson Laboratory (Bar Harbour, ME, USA) and backcrossed onto a C57BL6/J background for 10 generations. Mice were group‐housed in individually ventilated cages in a temperature‐controlled (approximately 21°C) room on a 12‐h light–dark cycle with free access to food and water. Animals were time‐mated overnight. Visual confirmation of a vaginal plug the following morning was considered embryonic day (E) 0.5.

Pregnant dams were killed by cervical dislocation, after which uterine horns were isolated and embryos were killed by decapitation. As previously described (Baker et al. 2018; Gordon et al. 2011), dissociated primary hippocampal‐enriched neuronal cultures were prepared from wild‐type C57BL6/J or α‐synuclein knockout (KO) E16.5–18.5 mouse embryos (with brains pooled from at least 3 embryos per culture) of both sexes by incubation of isolated hippocampi in papain followed by trituration in DMEM/F12 with 10% *v*/*v* FBS and 1% *v*/*v* penicillin/streptomycin, centrifugation at 363 *g* for 5 min and then resuspension of single dissociated cells in neurobasal media supplemented with 5% *v*/*v* B‐27, 0.5 mM L‐glutamine and 1% *v*/*v* penicillin/streptomycin. Hippocampal cultures were plated at a density of 3.5 × 10^4^ cells/coverslip on 13 mm coverslips in 24‐well plates for immunolabelling or 6 × 10^4^ cells/coverslip on 25 mm coverslips in 6‐well plates for live fluorescence imaging. Cultures were kept at 37°C and 5% CO_2_ in neurobasal media supplemented with 5% *v*/*v* B‐27, 0.5 mM L‐glutamine and 1% *v*/*v* penicillin/streptomycin, supplemented with 1 μM cytosine β‐d‐arabinofuranoside after 2–3 days to inhibit glial cell proliferation. Hippocampal cultures were transfected after 6–8 days in culture using Lipofectamine 2000 as previously described (Baker et al. 2018; Gordon et al. 2011). For fixed cultures, each coverslip was co‐transfected using 1 μL of Lipofectamine 2000 with 0.5 μg of either an empty vector (pcDNA3.1/Zeo(+); Addgene plasmid # V86020) or α‐synuclein plasmid, 0.5 μg of either syb2‐pHluorin or syt1‐pHluorin, and 0.5 μg of mCerulean. For live‐imaged cultures, each coverslip was co‐transfected using 2 μL Lipofectamine 2000 with 1 μg of either an empty vector or α‐synuclein, 1 μg of either syb2‐pHluorin or syt1‐pHluorin and 1 mg of mCherry, mCerulean or synaptophysin‐mCerulean.

### 2.4 HEK Cell Culture

HEK293T cells were maintained in Dulbecco's modified Eagle medium (DMEM) supplemented with 10% fetal bovine serum at 37°C and 5% CO_2_. For imaging PLA experiments, 2.5 × 10^4^ low passage HEK293T cells were seeded in 0.34 cm^2^ μ‐Slide 18 Well Glass coverslip from ibidi Cat# 81817 (Gräfelfing, Germany). For Western Blot, 3.0 × 10^5^ low passage HEK293T cells were seeded in 9.5 cm^2^ Costar 6‐well Clear Plate (Cat # 3516). After 24 h cells were co‐transfected with synaptobrevin‐2‐FLAG (syb2‐FLAG) and either WT α‐synuclein‐GFP or α‐synuclein^AAA^‐GFP carrying alanine substitutions within the KKD motif using Lipofectamine 3000 (Thermo Fisher Scientific) according to the manufacturer's instructions.

To assess synaptobrevin‐2‐FLAG and α‐synuclein‐GFP expression, HEK293T cells were washed twice with PBS and lysed in RIPA Buffer (50 mM Tris–HCl (pH 7.4), 150 mM NaCl, 1% Triton X‐100, 0.5% sodium deoxycholate, and 0.1% SDS supplemented with cOmplete Mini Protease Inhibitor Cocktail (Roche)). Lysates were incubated on ice for 20 min and clarified by centrifugation at 16000 × *g* for 10 min at 4°C. Equal amounts of clarified protein in RIPA Buffer were boiled in Laemmli sample buffer containing 50 mM DTT for 10 min at 95°C. Proteins were separated by SDS‐PAGE using a TGX Stain‐Free FastCast Acrylamide kit (Bio‐Rad) and UV‐activated for 5 min for stain‐free total‐protein imaging, followed by transfer to 0.22‐μm PVDF membranes using the iBlot 2 Transfer System (Thermo Fisher Scientific). Membranes were blocked in 3% (*w*/*v*) BSA in PBST and incubated with either mouse anti‐FLAG (1:2000, WEHI, Cat# KM5‐1C7) or rabbit anti‐GFP (1:2000, Invitrogen, Thermo Fisher Scientific, Cat# A‐6455) for 16 h at 4°C. Following incubation with either Goat anti‐Rabbit IgG (H + L) Secondary Antibody, HRP (1:2000 Thermo Fisher Scientific, Cat# 65‐6120) or Goat anti‐Mouse IgG (H + L) Secondary Antibody, HRP (1:2000 Thermo Fisher Scientific, Cat# 31430) for 1 h at Room Temperature, immunoreactive bands were detected with Clarity Western ECL substrate (Bio‐Rad) chemiluminescence. Following detection of α‐synuclein‐GFP, membranes were stripped using Pierce Restore PLUS Western Blot Stripping Buffer (Thermo Scientific) according to the manufacturer's instructions, washed extensively with PBST, and subsequently blocked and reprobed with mouse anti‐β‐actin antibody (1:2000 Abcam, Cat# 8226).

### 2.5 Proximity Ligation Assay (PLA)

24 h after transfection, HEK293T cells were washed with PBS, fixed with 4% paraformaldehyde in PBS for 10 min, and permeabilised for 5 min with 0.3% Triton X‐100 in PBS with 3× PBS washes between incubation steps. Proximity ligation assay was performed using the Duolink In Situ Red Starter Kit Mouse/Rabbit Cat# DUO92101‐1KT (Sigma‐Aldrich) according to the manufacturer's instructions. Briefly, cells were blocked with Duolink Blocking Solution (Cat# DUO82007) in a heated humidity chamber for 60 min at 37°C. Cells were incubated overnight at 4°C with mouse anti‐FLAG (1:500, WEHI, Cat# KM5‐1C7) and rabbit anti‐GFP (1:500, Invitrogen, Thermo Fisher Scientific, Cat# A‐6455) in Duolink Antibody Diluent (Cat# DUO82008). Cells were washed for 2× 5 min in 1× Wash Buffer A (Cat# DUO82049) at room temperature and then incubated with PLA probes: Duolink In Situ PLA Probe Anti‐Mouse MINUS (Cat# DUO92004) and Duolink In Situ PLA Probe Anti‐Rabbit PLUS (Cat# DUO92002) in antibody diluent for 1 h at 37°C. After two washes of 5 min with Wash Buffer A, the ligation step was performed with Ligase in Duolink Ligation buffer (Cat# DUO92008) diluted in UltraPure Water (Invitrogen, Thermo Fisher Scientific) for 30 min at 37°C. Cells were washed with Wash Buffer A twice for 5 min before incubation for 100 min at 37°C with Polymerase (Cat# DUO92008) in Amplificaiton Buffer (Cat# DUO92008) diluted in UltraPure Water (Invitrogen, Thermo Fisher Scientific). After two washes of 10 min with 1× Wash Buffer B (Cat# DUO82049) and one wash of 1 min with 0.01× Wash Buffer B in PBS, cells were mounted with Duolink In Situ Mounting Medium with DAPI (Cat# DUO82040). A negative control experiment was performed where either mouse anti‐FLAG or rabbit anti‐GFP were incubated with the PLA probes.

### 2.6 Fixation and Immunocytochemistry of Neurons

On DIV13‐14 primary hippocampal neuronal cultures were washed with standard saline buffer (136 mM NaCl, 2.5 mM KCl, 2 mM CaCl_2_, 1.3 mM MgCl_2_, 10 mM glucose, 10 mM HEPES, pH 7.4) and then fixed with 4% paraformaldehyde in phosphate buffered saline (PBS), quenched with 50 mM NH_4_Cl in PBS, permeabilised in 0.1% Triton X‐100 in 1% bovine serum albumin (BSA) in PBS, and blocked in 1% BSA in PBS as previously described (Baker et al. 2018). The cells were incubated with chicken anti‐GFP (which also recognises GFP derivatives pHluorin and mCerulean; 1:2000, Abcam, ab13970), mouse anti‐α‐synuclein (1:500, BD Biosciences, 610787), rabbit anti‐syb2 (1:1000, Abcam, ab3347) and/or rabbit anti‐syt1 (1:100, Synaptic Systems, 105102) primary antibodies diluted in 1% BSA in PBS for 2 h in a dark humidification chamber at room temperature. Cells were then incubated with goat anti‐chicken Alexa Fluor 488 (1:500, Thermo Fisher Scientific, A11039), goat anti‐chicken DyLight 550 (1:500, Thermo Fisher Scientific, SA5‐10071), donkey anti‐mouse DyLight 550 (1:200, Thermo Fisher Scientific, SA5‐10167) and/or donkey anti‐rabbit Alexa Fluor 647 (1:500, Thermo Fisher Scientific, A31573) secondary antibodies diluted in 1% BSA in PBS for 1 h (with three PBS washes after each incubation). Immunolabelled coverslips were washed in Milli‐Q Type 1 ultrapure H_2_O twice, dried and mounted on microscope slides with Dako Fluorescence Mounting Medium.

### 2.7 Fixed and Live Cell Fluorescence Imaging

Fixed cultures, trafficking assays and PLA experiment were imaged with a Zeiss Axio Observer 7 epifluorescence microscope. A Zeiss Plan‐Neofluar 40× oil‐immersion objective (NA 1.3) and Zeiss Axiocam 506 mono microscope camera were used to capture 14‐bit images with 2 × 2 binning resulting in pixels 0.227 μm in size. Alexa Fluor 488, α‐synuclein‐GFP and pHluorin‐tagged synaptobrevin‐2 or synaptotagmin‐1 were visualised using a 475 nm LED and a single bandpass 38 GFP filter (excitation 450–490 nm; emission 500–550 nm, beam splitter 495 nm). For the fixed cultures DyLight 550, Alexa Fluor 647 and PLA puncta were visualised with 567 nm and 630 nm LEDs respectively using a 90 HE DAPI/GFP/Cy3/Cy5 quadruple bandpass filter set (excitation 375–395, 455–483, 545–565, 620–643 nm; emission 410–440, 499–529, 579–604, 659–759 nm; beam splitters 405, 493, 575, 653 nm). For the live trafficking assays coverslips were mounted in a Warner imaging chamber with embedded parallel platinum wires (RC‐21BRFS), and mCherry was visualised using a 567 nm LED and a single bandpass 43 HE DsRed filter (excitation 538–562 nm, emission 570–640 nm, beam splitter 570 nm). Electrical field stimulation was applied using a Digitimer D330MultiStim System from SDR Scientific (Chatswood, Australia). Standard saline buffer (supplemented with 10 μM CNQX and 50 μM AP5) was perfused through the chamber at 37°C via a Warner TC‐324C in‐line heater. To quantify the trafficking of syb2−/syt1‐pHluorin, cultures were continuously perfused with standard saline buffer (supplemented with 10 μM CNQX and 50 μM AP5) and stimulated with either a single train of 300 action potentials (10 Hz, 35 mA), or four trains of 300 action potentials (1 train every 5 min, i.e., 10 Hz for 30 s, with a 4 min 30 s gap between trains). This was followed by perfusion with alkalising imaging buffer (86 mM NaCl, 2.5 mM KCl, 2 mM CaCl_2_, 1.3 mM MgCl_2_, 10 mM glucose, 10 mM HEPES, 50 mM NH_4_Cl, pH 7.4) to reveal total syb2‐/syt1‐pHluorin fluorescence. To quantify the movement of α‐synuclein‐GFP, cultures were continuously perfused with standard saline buffer (supplemented with 10 μM CNQX and 50 μM AP5) and stimulated with a train of 300 action potentials (10 Hz, 35 mA). Images were acquired at 1 Hz during baseline measurements, and then 0.5 Hz during and after stimulation as well as following perfusion with alkalising imaging buffer. For the 4× stimulation paradigm, the first 3 stimulation blocks were conducted in the absence of imaging, and images were captured during the final stimulation block of the experiment. Acid buffer was applied following the NH_4_Cl buffer for the 4× stimulation using syb2‐pHluoin to allow determination of the amount of syb2‐pHluorin on vesicles at the start of the 4th block.

The syb2‐pHluorin vesicular fraction assay was imaged using a Leica TCS SP8 inverted scanning confocal microscope inside an incubation chamber at 37°C and 5% CO_2_. A Leica HC PL APO CS2 40× (NA 1.3) oil‐immersion objective and hybrid photodetector were used to capture single‐channel, 16‐bit images of images with a pixel size of 0.284 μm. Syb2‐pHluorin was visualised with an Argon laser using an excitation wavelength of 496 nm. Confocal Z‐stacks including the entire volume of the nerve terminals of interest were acquired, and Nyquist sampling was used to calculate a *z*‐step size of 0.3 μm. Image analysis was performed on the summed projection of Z‐slices.

Determination of the vesicular fraction of syb2‐pHluorin was performed using a previously established protocol (Melland et al. 2023). Standard saline buffer was perfused over cultures to capture the surface population of syb2‐pHluorin (surface fluorescence), followed by acidic imaging buffer (136 mM NaCl, 2.5 mM KCl, 2 mM CaCl_2_, 1.3 mM MgCl_2_, 10 mM glucose, 20 mM MES, pH 5.5) to quench surface syb2‐pHluorin (background fluorescence), followed by a wash with standard saline imaging buffer. Alkalising NH_4_Cl imaging buffer was then perfused over cultures to reveal the total syb2‐pHluorin pool (total fluorescence).

### 2.8 Fluorescence Data Quantification

All images were processed using Image J 1.52 software (Schneider et al. 2012). Circular regions of interest (ROIs) with a diameter of 10 pixels (or 2 pixels for α‐synuclein‐GFP movement assay) were placed over nerve terminals using the Time Series Analyser V3 plugin (developed by Jayaprakash Balaji, Los Angeles, USA; https://imagej.nih.gov/ij/plugins/time‐series.html), and the total fluorescence intensity within each ROI was measured across conditions, buffers and/or time depending on the assay. XY‐drift was corrected in timeseries images for trafficking and vesicular fraction assays using rigid alignment via the MGBReg ImageJ plugin (developed by Donal Stewart, Edinburgh, Scotland).

Quantification of presynaptic protein expression in immunolabelled nerve terminals was performed as previously described (Baker et al. 2018). In brief, ROIs were placed over transfected (identified by anti‐GFP fluorescence) and untransfected boutons, as well as background regions (to measure autofluorescence) in sparsely populated fields of view, and relative protein levels at synapses calculated as a fluorescence ratio of transfected/non‐transfected protein expression levels (with background fluorescence subtracted). The diffuseness of fluorescence along axons was determined by calculating the coefficient of variation (CV) as previously described (Baker et al. 2018; Gordon et al. 2011). Briefly, single pixel‐wide ROI lines of at least 40 μm in length were drawn freehand along segments of transfected axon, and the fluorescence intensity of each pixel along these ROIs was measured. CV was calculated as the ratio between the standard deviation in fluorescence and the mean fluorescence along a ROI line. The vesicular fraction of syb2‐pHluorin as a percentage of total pHluorin was calculated using the equation: (total−surface fluorescence)/(total−background fluorescence) × 100.

For the syb2‐/syt1‐pHluorin trafficking assays, background autofluorescence decay was corrected (where possible) with the ImageJ Bleach Correction plugin (v2.0.3‐SNAPSHOT) (Schindelin et al. 2016; Miura 2020), using ≥ 20 ROIs overlaying areas in the field of view containing no cells, the “exponential decay” setting and all image frames prior to alkalising imaging buffer perfusion. ROIs were then laid over transfected boutons and synaptic fluorescence intensities measured. Each ROI subsequently underwent a rigorous screening process with strict inclusion/exclusion criteria. Only ROIs that increased fluorescence from baseline upon both electrical stimulation and NH_4_Cl buffer perfusion were included in subsequent analysis, and coverslips with greater than 20 responsive ROIs were included in the final data. Fluorescence responses of each ROI were calculated in Microsoft Excel as ΔF/F0 (change in fluorescence over baseline where baseline is the average fluorescence of the 5 frames before stimulation). For each frame of the time series, ΔF/F0 fluorescence values were averaged for all ROIs in a field of view, then baseline (i.e., 1) subtracted. The average fluorescence intensity traces were subsequently normalised to the peak fluorescence at the end of stimulation, or to the total fluorescence exhibited in response to the NH_4_Cl alkaline buffer.

For α‐syn‐GFP movement assay, ROIs were placed at the centre of transfected boutons and synaptic fluorescence intensities measured. Only ROIs that changed fluorescence from baseline upon electrical stimulation were included in analysis and coverslips with greater than 60 representative ROIs were included in the final data. Fluorescence responses of each ROI were calculated in Microsoft Excel as ΔF/F0 (change in fluorescence over baseline where baseline is the average fluorescence of the 5 frames before stimulation). For each frame of the time series, ΔF/F0 fluorescence values were averaged for all ROIs in a field of view, then baseline (i.e., 1) subtracted. The average fluorescence intensity trace was subsequently normalised to the peak fluorescence at the end of stimulation.

For quantification of PLA puncta, PLA images were background subtracted in Image J (rolling ball radius = 30 pixels) and thresholded using identical parameters across all conditions. Puncta were detected using the Particle Analysis tool in Image J (size = 0.02–2, circularity = 0.3–1.0). α‐synuclein‐GFP images were analysed separately. Images were thresholded and the % α‐synuclein‐GFP+ area was calculated. PLA puncta count was normalised to α‐synuclein‐GFP+ area (puncta count/mm^2^).

Chemiluminescent bands and Stain‐Free Total Protein blots were quantified using Image Lab software (v6.1; Bio‐Rad). The signal intensity of syb2‐FLAG and α‐synuclein‐GFP bands was normalised to total protein abundance from the stain‐free blot.

### 2.9 Statistical Analyses

Experimenters were blinded to the experimental condition (presence/absence of exogenous α‐synuclein) for image acquisition and analysis for fixed assays, and for image analysis for live assays. Statistical analyses were performed using GraphPad Prism Version 10.2.0 and Microsoft Excel software. Each data set was tested for normality using the Shapiro–Wilk test and statistical analyses were performed accordingly (unpaired two‐tailed Student's *t*‐tests or one‐way ANOVA with Dunnett's multiple comparisons post hoc test comparing to the control condition). Time courses of change in fluorescence were analysed via repeated measures two‐way repeated measures ANOVA with Sidak's multiple comparisons post hoc test. For all analyses, *p* < 0.05 was considered statistically significant. For live neuronal experiments (syt1‐pHluorin and syb2‐pHluorin trafficking, α‐synuclein‐GFP movement and vesicular fraction assays), ‘*n*’ refers to an individual field of view from an independent coverslip, with each experiment repeated across at least 3 independent cultures (at least 2 independent cultures for α‐syn‐GFP movement), and each culture comprising at least three embryos. For expression and CV analyses ‘*n*’ refers to an individual field of view from an independent coverslip; each experiment was repeated across two (α‐synuclein) or three (synaptobrevin‐2 and synaptotagmin‐1) independent cultures. For HEK293T cell PLA assay, ‘*n*’ is the average of five fields of view from an independent coverslip. Sample numbers are indicated in the graphs as single datapoints, and in the figure legends. No tests for outliers were conducted, and no data that met inclusion criterion has been excluded from this study. No strategy for randomisation of samples has been followed, and sample sizes were not chosen based on pre‐specified effect sizes. Instead, several independent experiments were performed across multiple independent cultures as indicated above and in figure legends, with sample size based on previous studies (Park et al. 2024; Melland et al. 2023; Melland et al. 2022; Marland et al. 2015). Full statistical results are available in Table S1.

## 3 Results

α‐Synuclein binds the SNARE protein synaptobrevin‐2 (Burre et al. 2012), and increased levels of α‐synuclein enhance SNARE complex assembly (Burre et al. 2010) without evident impact on exocytic rate. Therefore, we decided to investigate whether increased expression of α‐synuclein has direct impacts on synaptobrevin‐2 trafficking.

### 3.1 Moderate Overexpression of α‐Synuclein Does Not Alter Synaptobrevin‐2 or Synaptotagmin‐1 Protein Expression or Localisation

To explore whether the interaction between α‐synuclein and synaptobrevin‐2 might have functions beyond synaptic vesicle fusion, we created a model system in which the expression of α‐synuclein is moderately increased and examined how this influences synaptobrevin‐2 expression and distribution. Wild‐type primary hippocampal neurons were co‐transfected with human α‐synuclein (+α‐syn) or an empty vector (EV, pcDNA) and either synaptobrevin‐2‐pHluorin (syb2‐pHluorin) or synaptotagmin‐1‐pHuorin (syt1‐pHluorin) as alternative markers of synaptic vesicles. Regions of interest were placed over puncta to measure protein levels in nerve terminals. In this system, we moderately increased synaptic expression of α‐synuclein (Figure 1A,D; pcDNA 0.98 ± 0.02‐fold endogenous levels, +α‐syn 1.94 ± 0.08‐fold endogenous levels; see also Figure S1), with synaptic targeting of α‐synuclein at least as effective in neurons transfected with exogenous α‐synuclein as that in WT neurons (Figure S1). Increased synaptic expression of α‐synuclein did not impact the expression level of either synaptobrevin‐2 (Figure 1B,E; pcDNA 1.76 ± 0.10, +α‐syn 1.58 ± 0.05) or synaptotagmin‐1 (Figure 1C,F; pcDNA 2.03 ± 0.12, +α‐syn 1.82 ± 0.07). We then examined the enrichment of these synaptic vesicle proteins at synaptic terminals (Figure 1G–J). To quantify this, we determined the coefficient of variation (CV), calculated as the ratio between the variability in fluorescence and the mean fluorescence along a length of axon. Enrichment of proteins at nerve terminals is indicated by a high CV, while de‐enrichment from terminals combined with a relative increase in axonal expression decreases CV. Increased expression of α‐synuclein had no impact on the CV of syb2‐pHluorin (Figure 1G,H; pcDNA 42.20 ± 2.54, +α‐syn 42.71 ± 1.02) or syt1‐pHluorin (Figure 1I,J; pcDNA 43.28 ± 2.50, +α‐syn 45.62 ± 2.69). This means that increasing the expression of α‐synuclein did not impact the ability of either synaptobrevin‐2 or another vesicle cargo protein, synaptotagmin‐1, to be expressed or targeted to nerve terminals.

**FIGURE 1:**
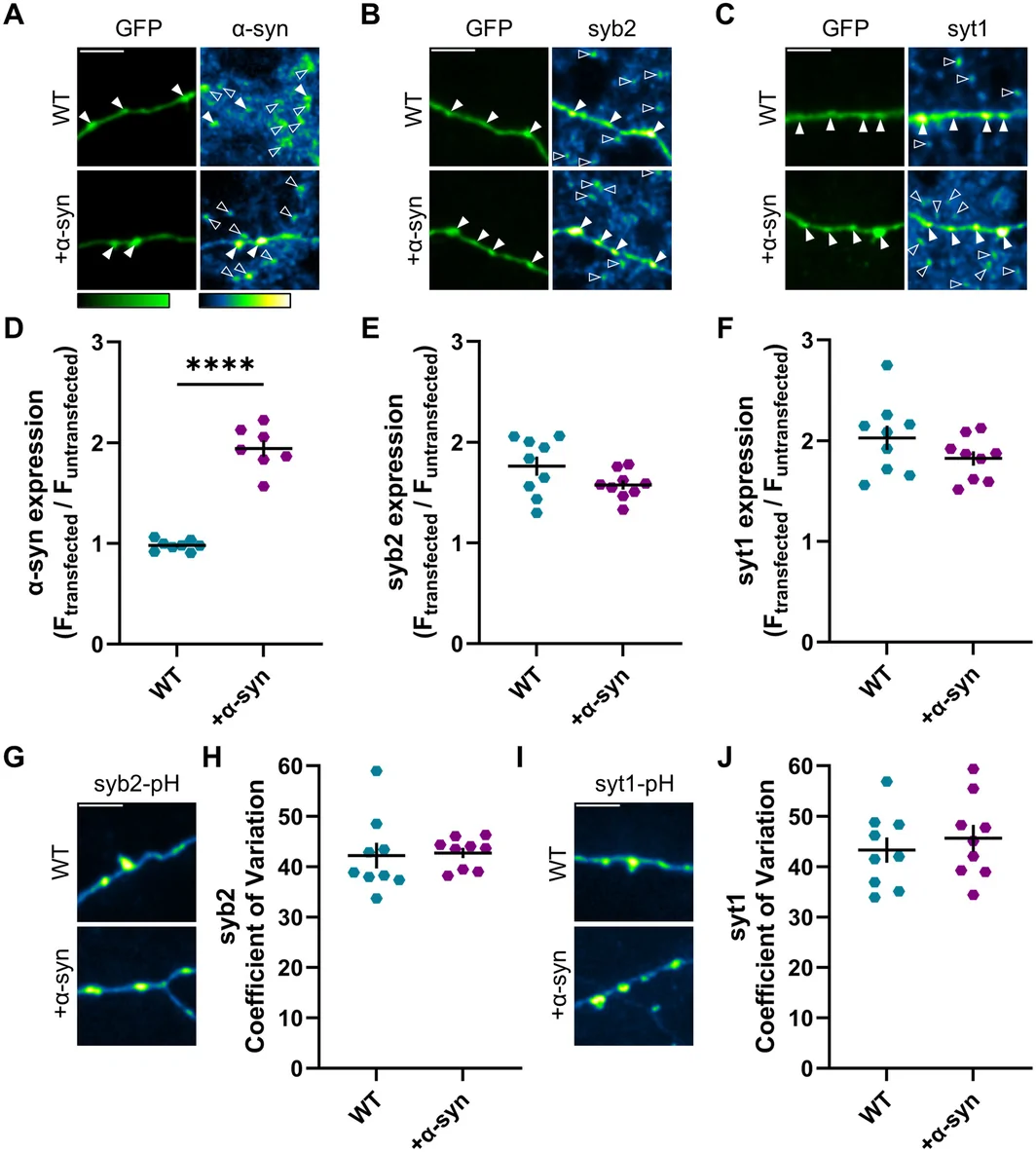
Expression and localisation of specific synaptic vesicle proteins is independent of α‐synuclein expression. (A–C) Representative images of hippocampal neurons that were transfected with either an empty vector (pcDNA) or α‐synuclein (+α‐syn), syt1‐pHluorin or syb2‐pHluorin, and mCerulean, then fixed and immunolabelled with anti‐GFP antibody (which recognises GFP derivatives pHluorin and mCerulean) to identify transfected neurites (left panels) and α‐synuclein (α‐syn, A), synaptobrevin‐2 (syb2, B) or synaptotagmin‐1 (syt1, C) (right panels). Filled arrows indicate transfected nerve terminals while empty arrows indicate un‐transfected nerve terminals. Scale bar is 5 μm. (D–F) Relative expression of α‐syn (D), syb2 (E) or syt1 (F) calculated as a mean ratio of the fluorescence intensity (F) of transfected to un‐transfected nerve terminals with background subtracted. ****p < 0.0001 for α‐syn (D), *p* = 0.098 for syb2 (E) and *p* = 0.168 for syt1 (F) comparing pcDNA and + α‐syn, unpaired two‐tailed Student's *t*‐tests. (G,I) Representative images of hippocampal neurons co‐transfected with syb2‐pHluorin or syt1‐pHluorin and either an empty vector (pcDNA) or α‐synuclein (+α‐syn) that were fixed and immunolabelled with anti‐GFP antibody (which recognises pHluorin). Scale bars are 5 μm. (H, J) Coefficient of variation (CV) analysis was used to determine the distribution of syb2 and syt1 along axonal segments within cultures shown in G and I respectively. *p* = 0.853 for syb2 (H) and *p* = 0.535 for syt1 comparing pcDNA and + α‐syn, unpaired two‐tailed Student's *t*‐tests. (D–F, H, J) Plots show individual *n* and mean ± SEM, *n* = 7 (+α‐syn in D), 8 (pcDNA in D), or 9 (+α‐syn and pcDNA in E, F, H, J) individual coverslips from 2 (D) or 3 (E, F, H, J) independent cultures. See Table S1 for full statistical tests.

### 3.2 Increased Expression of α‐Synuclein Impairs Retrieval of Synaptobrevin‐2

We next wanted to explore whether modestly increased expression of α‐synuclein alters synaptobrevin‐2 trafficking in an activity‐dependent manner. Wild‐type neurons were co‐transfected with syb2‐pHluorin and either an empty vector (pcDNA) or α‐synuclein (+α‐syn). pHluorin is a pH‐sensitive GFP, which is tagged to the lumenal domain of a synaptic vesicle protein of interest. pHluorin fluorescence is quenched by the acidic environment within the synaptic vesicle and increases when exposed to the neutral extracellular environment. pHluorin fluorescence thus increases with synaptic vesicle exocytosis and then decreases upon retrieval of the pHluorin‐tagged protein to endocytosing vesicles and vesicle re‐acidification (Figure 2A). Perfusion of neurons with NH_4_Cl buffer deacidifies vesicles and allows visualisation and measurement of the entire pHluorin pool. We stimulated neurons with 300 action potentials (AP; 10 Hz, 35 mA) which triggers exo‐ and endocytosis of the recycling pool of vesicles and allows us to follow the trafficking of synaptobrevin‐2 throughout the synaptic vesicle cycle (Figure 2B). Upon stimulation, the peak amplitude of syb2‐pHluorin fluorescence induced by the stimulus train (as a proportion of total pHluorin fluorescence revealed with NH_4_Cl) was not impacted by increased expression of α‐synuclein (Figure 2C; WT 0.35 ± 0.03, +α‐syn 0.37 ± 0.02). Traces were thus normalised to stimulus‐induced peak amplitude to examine whether there were any changes to the rate of trafficking of syb2‐pHluorin (Figure 2D). The rate of fluorescence change over the first 10 s of stimulation was measured, revealing that the rate of increase in syb2‐pHluorin fluorescence was similar regardless of α‐synuclein levels (Figure 2E; WT 0.0174 ΔF/F_0_ s^−1^ ± 0.0018, +α‐syn 0.0167 ΔF/F_0_ s^−1^ ± 0.0015), suggesting no change to exocytosis. However, increased expression of α‐synuclein significantly impaired the rate at which syb2‐pHluorin fluorescence returned to baseline following the cessation of stimulation (Figure 2D, *p* < 0.05 between WT and + α‐syn from 78 s onwards). To confirm this, we determined the percent recovery to baseline 180 s after the initiation of the stimulus pulse, revealing that increased expression of α‐synuclein caused a significant decrease in recovery (Figure 2F; WT 83.51% ± 1.61%, +α‐syn 68.59% ± 1.76%). This could be due to either a defect in endocytosis or a specific impairment in synaptobrevin‐2 retrieval to endocytosing vesicles.

**FIGURE 2:**
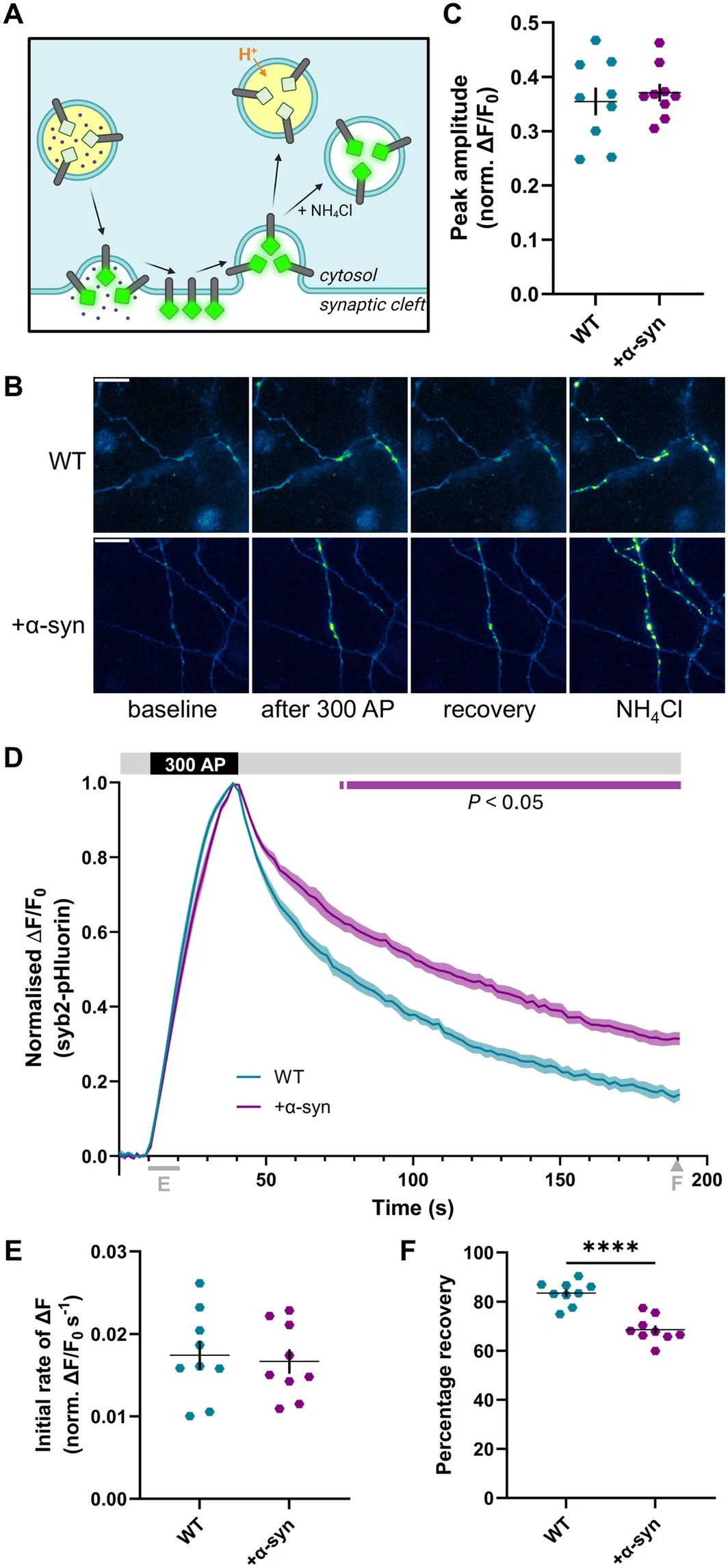
Modestly increased expression of α‐synuclein slows the endocytic retrieval of synaptobrevin‐2. Hippocampal neurons co‐transfected with syb2‐pHluorin, mCherry (transfection marker), and either an empty vector (WT) or α‐synuclein (+α‐syn) were stimulated with a train of 300 action potentials (300 AP, 10 Hz, 35 mA, indicated by labelled black bar) and the change in syb2‐pHluorin fluorescence over time was measured. Neurons were then perfused with NH_4_Cl to reveal the total syb2‐pHluorin fluorescence. (A) Schematic of pHluorin fluorescence in different environments. (B) Representative images of syb2‐pHluorin fluorescence at baseline, after 300 AP, at 180 s (recovery, as calculated in F), and under NH_4_Cl perfusion. (C) Peak amplitude (mean maximum evoked ΔF/F_0_) of syb2‐pHluorin fluorescence during stimulation, normalised to total fluorescence. *p* = 0.6070, unpaired two‐tailed Student's *t*‐test. (D) Time course of mean ΔF/F_0_ ± SEM of syb2‐pHluorin fluorescence normalised to peak amplitude during stimulation. Shading indicates SEM, *p < 0.05 for WT versus +α‐syn over time indicated by labelled purple line at 74 s and from 78 s onwards, two‐way repeated measures ANOVA with Sidak's multiple comparisons post hoc test. Time frames used for calculating data in D and E are indicated. (E) Mean initial rate of fluorescence change calculated from linear fit of ΔF/F_0_ per second over the first 10 s of stimulation, normalised to total fluorescence. *p* = 0.7504, unpaired two‐tailed Student's *t*‐test. (F) Mean percent recovery to baseline. ****p < 0.0001, unpaired two‐tailed Student's *t*‐test. (C–F) Plots show individual *n* and mean ± SEM, *n* = 9 individual coverslips from at least 3 independent cultures. See Table S1 for full statistical tests.

To differentiate between these two possibilities, wild‐type neurons were co‐transfected with α‐synuclein or empty vector as well as syt1‐pHluorin, as an independent marker of synaptic vesicle dynamics. Neurons were stimulated with 300 AP (10 Hz, 35 mA) as before and the change in syt1‐pHluorin fluorescence was measured as an indicator of vesicle trafficking (Figure 3A,B). Unlike what was observed for syb2‐pHluorin, increased expression of α‐synuclein did not alter the trafficking of syt1‐pHluorin fluorescence over the time course of the experiment (Figure 3B). Overexpression of α‐synuclein did not impact either the peak amplitude of syt1‐pHluorin fluorescence change (Figure 3C; WT 0.28 ± 0.01, +α‐syn 0.26 ± 0.01) or rate of increase in syt1‐pHluorin fluorescence in the first 10 s of stimulation (Figure 3D; WT 0.0201 ± 0.0010, +α‐syn 0.0178 ± 0.0011), nor did it alter the percent of fluorescence recovery after 180 s (Figure 3E, WT 87.03% ± 1.581%, +α‐syn 87.59% ± 1.684%). There was also no change in the overall rate of re‐quenching of syt1‐pHluorin fluorescence following the end of the stimulus train (measured as tau; Figure 3F, WT 19.43 s ± 2.59, +α‐syn 17.94 s ± 1.57). Collectively, this indicates that increased expression of α‐synuclein does not impact the rate of endocytosis nor the rate of vesicle reacidification, but instead specifically slows the retrieval of synaptobrevin‐2 during endocytosis.

**FIGURE 3:**
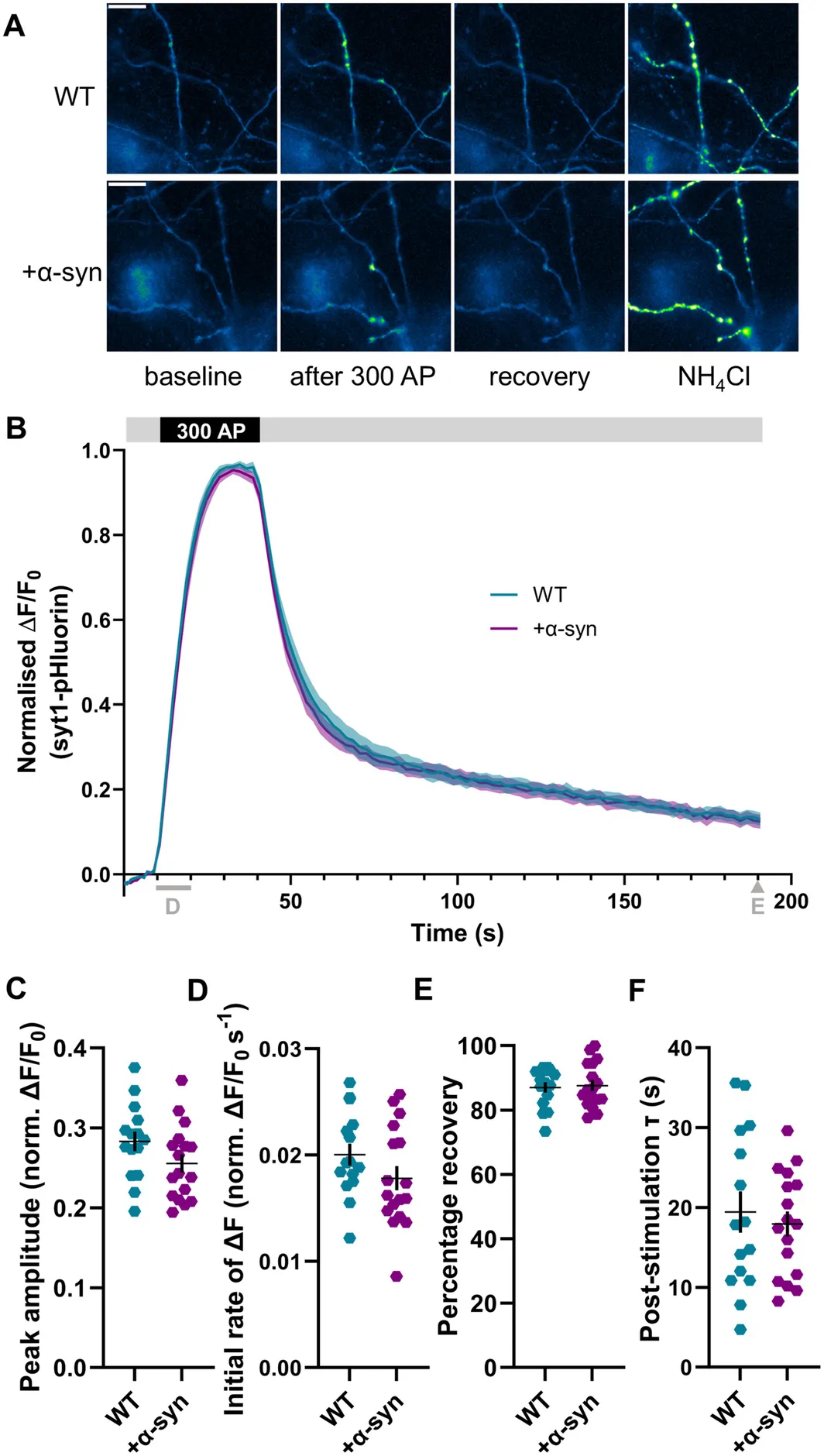
Modestly increased expression of α‐synuclein does not impact synaptic vesicle cycling. Hippocampal neurons co‐transfected with syt1‐pHluorin, mCherry and either an empty vector (WT) or α‐synuclein (+α‐syn) were stimulated with a train of 300 AP (10 Hz, 35 mA, indicated by labelled black bar) and the change in syt1‐pHluorin fluorescence over time was measured. Neurons were then perfused with NH_4_Cl to reveal the total syt1‐pHluorin fluorescence. (A) Representative images of syt1‐pHluorin fluorescence at baseline, after 300 AP, at 180 s (recovery, as calculated in E), and under NH_4_Cl perfusion. (B) Time course of mean ΔF/F_0_ ± SEM of syt1‐pHluorin fluorescence normalised to peak amplitude during stimulation. Shading indicates SEM, *p* > 0.05 at all timepoint according to a two‐way repeated measures ANOVA with Sidak's multiple comparisons post hoc test. Time frames used for calculating data in (D) and (E) are indicated. (C) Peak amplitude (mean maximum evoked ΔF/F_0_) of syt1‐pHluorin fluorescence during stimulation, normalised to total fluorescence. *p* = 0.1078, unpaired two‐tailed Student's *t*‐test. (D) Mean initial rate of fluorescence change calculated from linear fit of ΔF/F_0_ per second over the first 10 s of stimulation, normalised to total fluorescence. *p* = 0.1632, unpaired two‐tailed Student's *t*‐test. (E) Mean percent recovery to baseline. *p* = 0.8117, unpaired two‐tailed Student's *t*‐test. (F) Time constant (tau) of post‐stimulus re‐quenching of syt1‐pHluorin. *p* = 0.6143, unpaired two‐tailed Student's *t*‐test. (B–F) Plots show individual *n* and mean ± SEM, *n* = 15 (WT) or 17 (+α‐syn) individual coverslips from at least 3 independent cultures. See Table S1 for full statistical tests.

### 3.3 α‐Synuclein Does Not Work Antagonistically to Synaptophysin

We and others have previously demonstrated that synaptophysin is important for synaptobrevin‐2 retrieval during endocytosis (Gordon et al. 2011; Bonanomi et al. 2007; Gordon et al. 2016), such that exogenous synaptophysin increases the vesicular fraction of synaptobrevin‐2 (Bonanomi et al. 2007; Gordon et al. 2016). To investigate whether α‐synuclein may be acting in competition with synaptophysin, we co‐transfected wild‐type neurons with syb2‐pHluorin and α‐synuclein or synaptophysin, alone or in combination, and investigated the partitioning of syb2‐pHluorin to vesicles. Neurons were perfused with acidic buffer to quench all surface‐localised syb2‐pHluorin and then NH_4_Cl buffer to reveal total pHluorin fluorescence; this allowed us to determine the proportion of plasma membrane‐ versus vesicular‐localised syb2‐pHluorin (Figure 4A). In comparison to the control condition (Figure 4B; pcDNA + mCer 64.48% ± 1.95%), the addition of synaptophysin significantly increased the amount of vesicular synaptobrevin‐2 (Figure 4B; pcDNA + syp 71.48% ± 1.75%). However, increasing the expression of α‐synuclein had no impact on the proportion of vesicular synaptobrevin‐2 (Figure 4B; α‐syn + mCer 63.48% ± 2.20%). Moreover, α‐synuclein did not work antagonistically to synaptophysin, as exogenous delivery of synaptophysin increased the amount of vesicular synaptobrevin‐2 in the absence or presence of excess α‐synuclein (Figure 4B; α‐syn + syp 74.28% ± 1.28%). This suggests that α‐synuclein acts in a distinct manner to synaptophysin and does not compete with its function.

**FIGURE 4:**
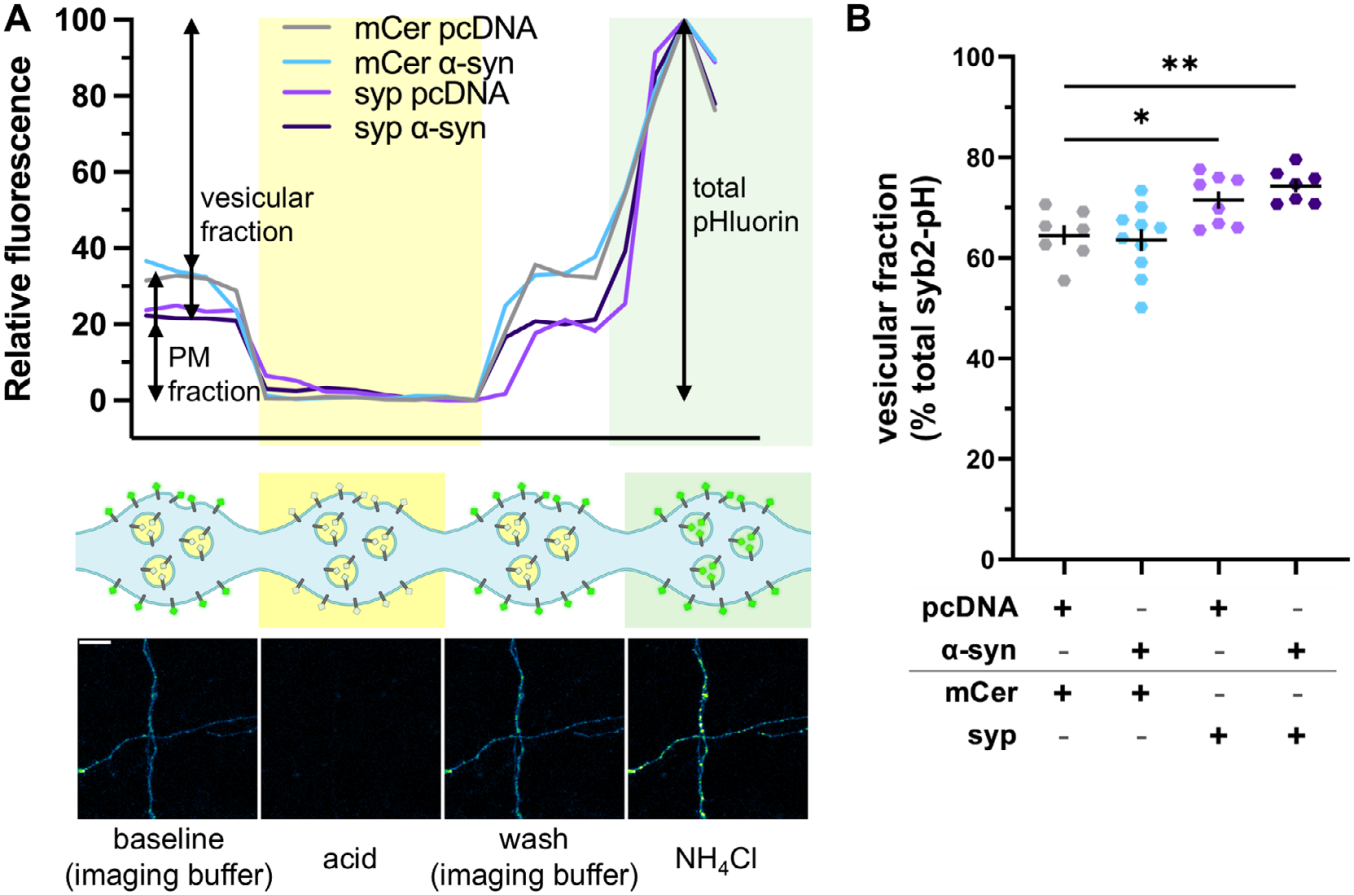
Slowed retrieval of synaptobrevin‐2 with modest α‐synuclein overexpression is not caused by competition between α‐synuclein and synaptophysin. Wild‐type hippocampal neurons were co‐transfected with syb2‐pH, and either an empty vector (pcDNA) or α‐synuclein (α‐syn), and either mCerulean (mCer) or synaptophysin‐mCerulean (syp). Neurons were then perfused with neutral imaging buffer, acid buffer, or alkalising NH_4_Cl buffer and changes in pHluorin fluorescence monitored. (A) Representative traces (top) and images (bottom) of changes in pHluorin fluorescence (traces normalised to 0% in acidic buffer and 100% in NH_4_Cl buffer). Schematic (middle) demonstrates the pools of pHluorin in presynaptic boutons that are fluorescing (green) or quenched (grey) in the presence of different buffers. Yellow denotes an acidic environment, pale green is NH_4_Cl buffer, and white denotes a neutral environment. (B) Vesicular fraction of syb2‐pHluorin. Plots show individual *n* and mean ± SEM internal (vesicular) syb2‐pHluorin as a percentage of total pHluorin fluorescence, *p* = 0.9643 for α‐syn + mCer, *p = 0.0495 for pcDNA + syp, **p = 0.0062 for α‐syn + syp, one‐way ANOVA with Dunnett's multiple comparisons post hoc test compared to the control condition (pcDNA + mCer). *n* = 7 (pcDNA + mCer, α‐syn + syp), 8 (pcDNA + syp), or 10 (α‐syn + mCer) individual coverslips from at least 3 independent cultures. See Table S1 for full statistical tests.

### 3.4 Direct Binding of α‐Synuclein to Synaptobrevin‐2 Underlies Slowed Synaptobrevin‐2 Trafficking

α‐Synuclein binds directly with synaptobrevin‐2 through a region C‐terminal to the membrane‐binding domain (Burre et al. 2010; Burre et al. 2012) (Figure 5A), and mutation of amino acids 96–98 to alanine (KKD > AAA) reduces binding to synaptobrevin‐2 (Sun et al. 2019). We confirmed via proximity ligation assay that KKD>AAA mutant α‐synuclein (α‐syn^AAA^) has a reduced interaction with synaptobrevin‐2 (Figures S2 and S3). When expressed in neurons, KKD>AAA mutant α‐synuclein is expressed at a similar level as the exogenously introduced WT α‐synuclein (Figure S4), displays similar steady‐state protein levels (Figure S5), and targets as effectively to nerve terminals as the WT protein (Figure S5). To test whether increased expression of α‐synuclein impairs synaptobrevin‐2 trafficking through a direct interaction, neurons were co‐transfected with syb2‐pHluorin in the presence of empty vector (WT), α‐synuclein (+α‐syn), or with KKD>AAA mutant α‐synuclein (+α‐syn^AAA^). Neurons were then stimulated with 300 AP, and changes in syb2‐pHluorin fluorescence monitored (Figure 5B). We found that the peak amplitude of syb2‐pHluorin fluorescence change induced by 300 AP stimulation (as a proportion of total pHluorin fluorescence revealed with NH_4_Cl) was not impacted by either increased expression of α‐synuclein (consistent with Figure 2B) or the presence of the KKD > AAA mutant (Figure 5C; WT 0.37 ± 0.03, +α‐syn^WT^ 0.32 ± 0.035, +α‐syn^AAA^ 0.35 ± 0.03), nor was the initial rate of fluorescence change (Figure 5D; WT 0.0590 ± 0.0028, +α‐syn^WT^ 0.0528 ± 0.0025, +α‐syn^AAA^ 0.0565 ± 0.0023), supporting that there was no impact to exocytosis. Increased expression of α‐synuclein once again impaired the retrieval of synaptobrevin‐2 (Figure 5B,E; WT 74.80% ± 3.41%, +α‐syn^WT^ 56.50% ± 3.46%). However, this effect was strongly attenuated by the KKD>AAA mutation. Synaptobrevin‐2 retrieval was only mildly impaired in the presence of α‐synuclein mutant with reduced synaptobrevin‐2 binding affinity (Figure 5B, *p* < 0.05 between WT and + asyn^WT^ from 45 s onwards compared to between 144 and 148 s only for +α‐syn^AAA^). Additionally, unlike what was observed with overexpression of WT α‐synuclein, expression of the α‐synuclein KKD mutant did not significantly impact the recovery of syb2‐pHluorin fluorescence (Figure 5E, +α‐syn^AAA^ 67.07% ± 2.03%), suggesting that excess α‐synuclein impairs retrieval of synaptobrevin‐2 via a direct interaction between the two proteins.

**FIGURE 5:**
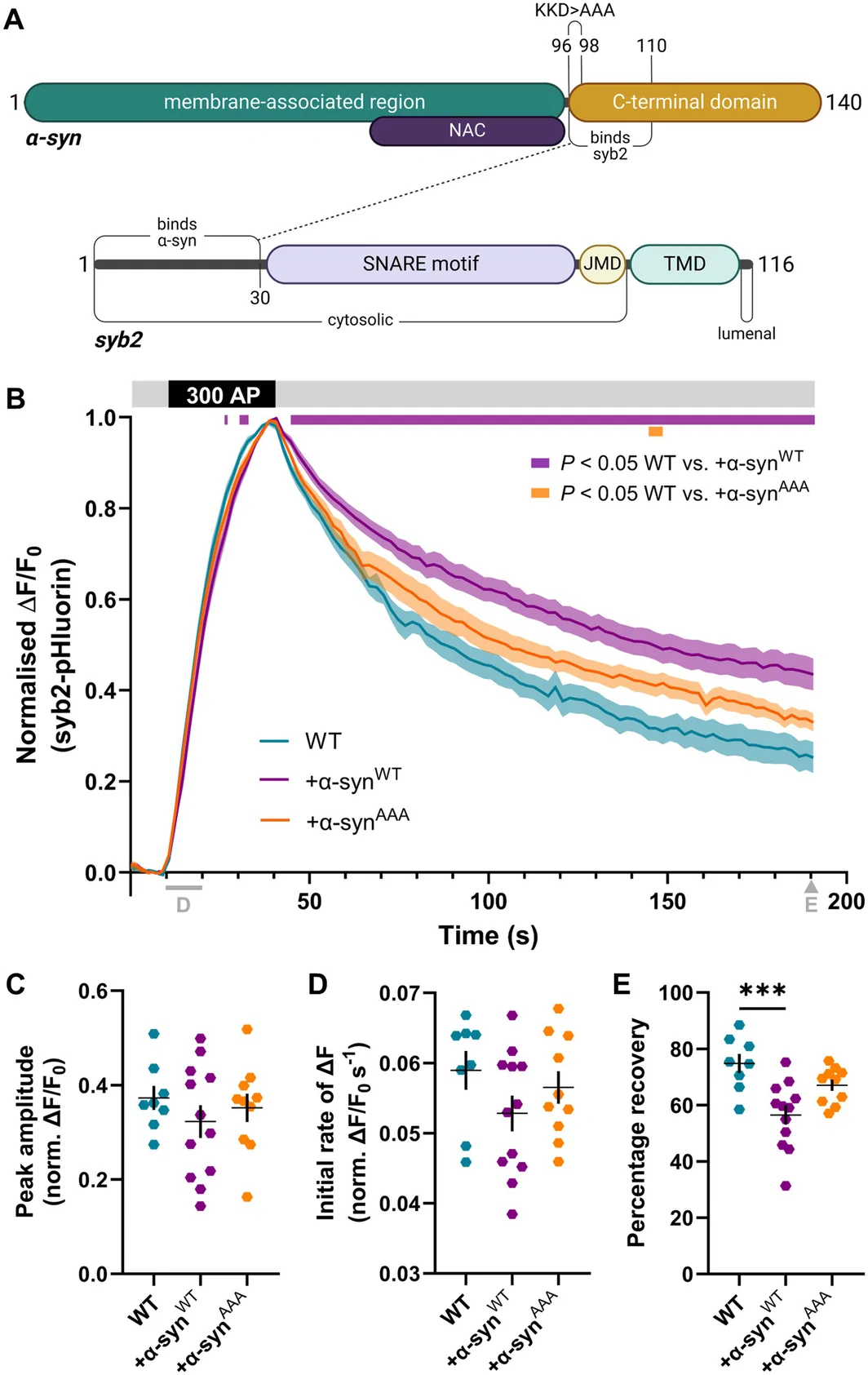
Slowed retrieval of synaptobrevin‐2 with modest α‐synuclein overexpression is a result of direct interaction between synaptobrevin‐2 and α‐synuclein. (A) Schematic of binding sites between α‐synuclein (α‐syn) and synaptobrevin‐2 (syb2). Mutation of amino acids 96–98 (KKD) in α‐synuclein to alanine (AAA) strongly reduces binding to synaptobrevin‐2. JMD = juxtamembrane domain, NAC = non‐amyloid‐β component domain, TMD = transmembrane domain. (B–E) Hippocampal neurons co‐transfected with syb2‐pHluorin, mCherry (transfection marker), and either an empty vector (WT), α‐synuclein (+α‐syn) or α‐synuclein with reduced synaptobrevin‐2 binding affinity (+α‐syn^AAA^) were stimulated with a train of 300 AP (10 Hz, 35 mA, indicated by labelled black bar) and the change in syb2‐pHluorin fluorescence over time was measured. Neurons were then perfused with NH_4_Cl to reveal the total syb2‐pHluorin fluorescence. See Figure S6 for representative images of syb2‐pHluorin fluorescence. (B) Time course of mean ΔF/F_0_ ± SEM of syb2‐pHluorin fluorescence normalised to peak amplitude during stimulation. Shading indicates SEM, *p < 0.05 for WT versus + α‐syn (purple) or + α‐syn^AAA^ (orange) over time indicated by coloured lines (purple +α‐syn: 26, 30, 32 and 44 s onwards; orange +α‐syn^AAA^: 144–148 s), two‐way repeated measures ANOVA with Sidak's multiple comparisons post hoc test. Time frames used for calculating data in (D) and (E) are indicated. (C) Peak amplitude (mean maximum evoked ΔF/F_0_) of syb2‐pHluorin fluorescence during stimulation, normalised to total fluorescence. *p* = 0.4476 for WT versus +asyn, *p* = 0.8667 for WT versus + α‐syn^AAA^, one‐way ANOVA with Dunnett's multiple comparisons test. (D) Mean initial rate of fluorescence change calculated from linear fit of ΔF/F_0_ per second over the first 10 s of stimulation, normalised to total fluorescence. *p* = 0.1815 for WT versus + asyn, *p* = 0.7395 for WT versus + α‐syn^AAA^, one‐way ANOVA with Dunnett's multiple comparisons test. (E) Mean percent recovery to baseline. *p* = 0.0007 for WT versus + asyn, *p* = 0.1827 for WT versus + α‐syn^AAA^, one‐way ANOVA with Dunnett's multiple comparisons test. (C–E) Plots show individual *n* and mean ± SEM, *n* = 8 (WT), 12 (+asyn^WT^) or 10 (+α‐syn^AAA^) individual coverslips from at least 3 independent cultures. See Table S1 for full statistical tests.

### 3.5 Modest Overexpression of α‐Synuclein Induces Presynaptic Depression Over Time

We next sought to determine the consequences of impaired synaptobrevin‐2 trafficking on synaptic function. Impaired synaptobrevin‐2 retrieval causes a reduction in the copy number of vesicular synaptobrevin‐2 over time (Kokotos et al. 2019). This would be predicted to cause a decrease in presynaptic strength with repeated stimulation. To examine this, wild‐type neurons were subjected to repeated stimulus trains (4 trains of 300 AP, 10 Hz, 1 train every 5 min), and the change in syt1‐pHluorin fluorescence monitored over the final (fourth) stimulus acquisition as a measure of vesicle cycling (Figure 6A). Upon a single stimulus train, the peak amplitude of syt1‐pHluorin fluorescence evoked by 300 AP is unaffected by moderate overexpression of α‐synuclein (Figure 3A). However, upon the fourth stimulus train there is a pronounced decrease in syt1‐pHluorin cycling in the presence of increased levels of α‐synuclein compared to WT neurons transfected with empty vector (Figure 6B), resulting in a decreased peak amplitude of syt1‐pHluorin fluorescence (Figure 6C, WT 0.27 ± 0.03, +α‐syn 0.20 ± 0.02) and a trend towards reduced linear rate of fluorescence increase (Figure 6D, WT 0.0173 ± 0.0029, +α‐syn 0.0109 ± 0.0021). Notably, the introduction of an α‐synuclein mutant with reduced synaptobrevin‐2 binding (KKD > AAA) did not cause the same decrease in syt1‐pHluorin cycling with repeated stimulation (Figure 6B, *p* > 0.05) compared to WT across all time points, resulting in a similar peak amplitude of stimulus‐evoked syt1‐pHluorin fluorescence (Figure 6C, +asyn^AAA^ 0.27 ± 0.02) and rate of fluorescence increase (Figure 6D, +asyn^AAA^ 0.0182 ± 0.0020) as observed in WT neurons. We observe the same trend in synaptic depression when using the syb2‐pHluorin reporter (Figure S7). This suggests that modest overexpression of α‐synuclein induces synaptic depression over time, in a manner dependent on direct binding to synaptobrevin‐2.

**FIGURE 6:**
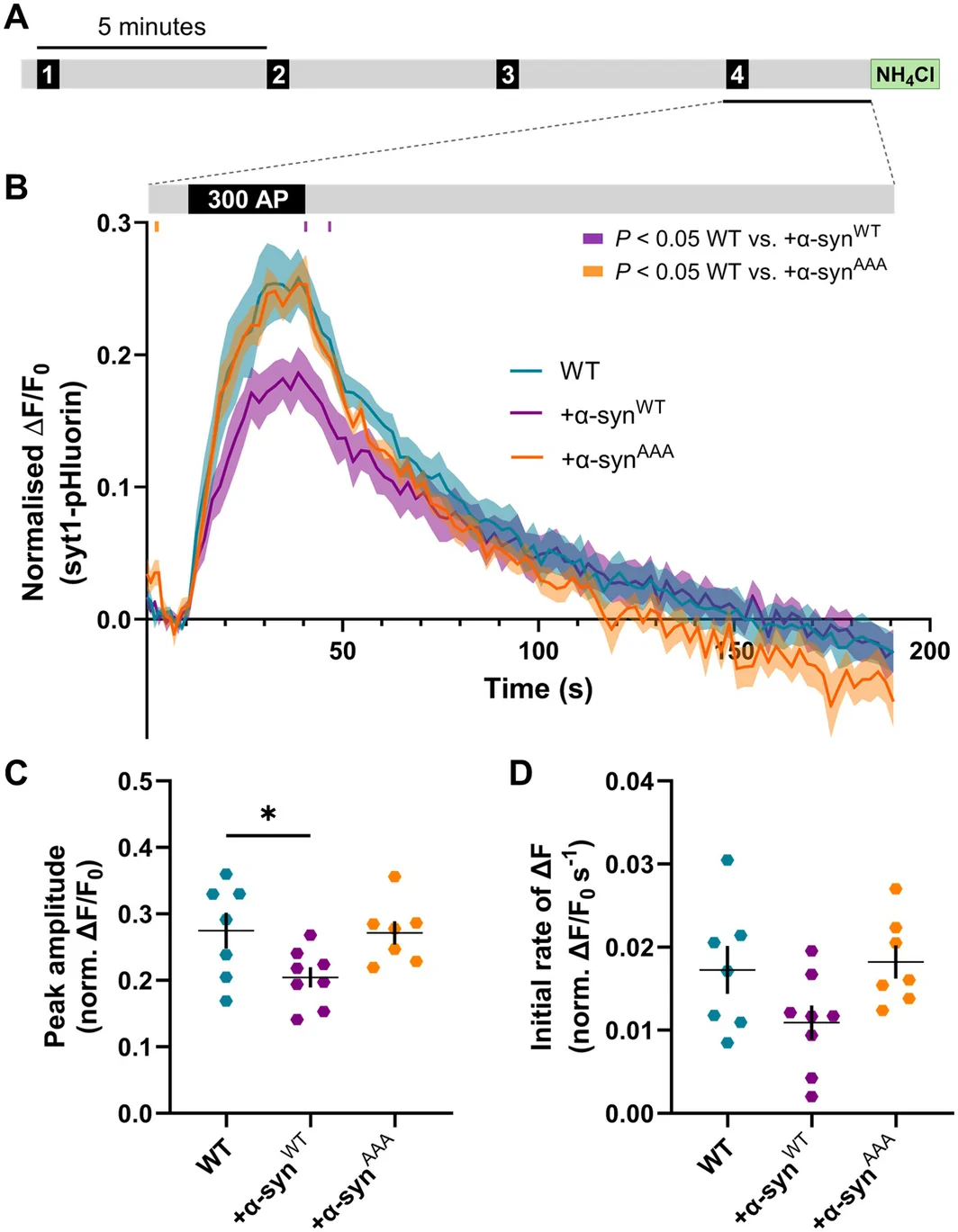
Modest α‐synuclein overexpression induces synaptic depression with repeated stimulation. (A) Experimental paradigm. Hippocampal neurons co‐transfected with syt1‐pHluorin, mCherry (transfection marker), and either an empty vector (WT), α‐synuclein (+α‐syn) or α‐synuclein with reduced synaptobrevin‐2 binding affinity (+α‐syn^AAA^) were stimulated with trains of 300 action potentials (10 Hz, 35 mA, indicated by labelled black bar) every 5 min, and the change in syt1‐pHluorin fluorescence over time was measured across the final (fourth) stimulus train. Neurons were then perfused with NH_4_Cl to reveal the total syt1‐pHluorin fluorescence. See Figure S6 for representative images of syt1‐pHluorin fluorescence. (B) Time course of mean ΔF/F_0_ ± SEM of syt1‐pHluorin fluorescence, across the fourth stimulus train, normalised to total pHluorin fluorescence upon NH_4_Cl. Shading indicates SEM. *p < 0.05 for WT versus either +asyn (purple) or +α‐syn^AAA^ (orange) over time indicated by coloured bars, two‐way repeated measures ANOVA with Dunnett's multiple comparisons post hoc test. (C) Peak amplitude (mean maximum evoked ΔF/F_0_) of syt1‐pHluorin fluorescence during stimulation, normalised to total fluorescence. *p* = 0.0407 for WT versus + asyn, *p* = 0.9905 for WT versus +α‐syn^AAA^, one‐way ANOVA with Dunnett's multiple comparisons test. (D) Mean initial rate of fluorescence change calculated from linear fit of ΔF/F_0_ per second over the first 10 s of stimulation, normalised to total fluorescence. *p* = 0.1177 for WT versus + asyn, *p* = 0.9424 for WT versus +α‐syn^AAA^, one‐way ANOVA with Dunnett's multiple comparisons test. (C, D) Plots show individual *n* and mean ± SEM, *n* = 7 (WT and + α‐syn^AAA^) or 8 (+asyn^WT^) individual coverslips from at least 3 independent cultures. See Table S1 for full statistical tests.

## 4 Discussion and Conclusion

We have demonstrated that modestly increasing the expression of α‐synuclein in neurons specifically slows the endocytic retrieval of synaptobrevin‐2, likely via a direct interaction between α‐synuclein and synaptobrevin‐2. Impaired endocytosis has previously been observed in triple αβγ‐synuclein knockout synapses (Vargas et al. 2014). However, the slowing of synaptobrevin‐2 retrieval observed in the present study upon α‐synuclein overexpression was not due to a change in global endocytosis; this is consistent with observations by Nemani et al. (2010) who also noted a similar lack of effect on global endocytic rate in hippocampal cultures with mild α‐synuclein overexpression, as measured with the synaptic vesicle marker VGLUT1‐pHluorin (Nemani et al. 2010). Furthermore, the impaired retrieval was not driven by a relative difference in synaptobrevin‐2 expression, nor was altered trafficking at rest observed through a change in either nerve terminal targeting or the level of vesicular synaptobrevin‐2. Notably, presynaptic depression is observed with repeated stimulation when α‐synuclein is modestly overexpressed, in a manner that is likely dependent on direct binding to synaptobrevin‐2.

In this model system we have approximately doubled the amount of α‐synuclein in nerve terminals of primary hippocampal cultures (Figures 1A,D and S1). This level reflects what has been observed in individuals with triplication of the *SNCA* gene encoding α‐synuclein (Farrer et al. 2004; Miller et al. 2004), which is a known genetic cause of Parkinson's disease (PD) (Olgiati et al. 2015; Singleton et al. 2003). As such, the perturbation in synaptobrevin‐2 retrieval may contribute to the underlying synaptic dysfunction observed in neurons derived from PD patients with *SNCA* triplication (Maor et al. 2023).

It is important to note that we have used exogenously expressed syb2‐pHluorin as a monitor of synaptobrevin‐2 trafficking. Syb2‐pHluorin has been used extensively to monitor both synaptic vesicle (Granseth et al. 2006; Gandhi and Stevens 2003; Burrone et al. 2006) and synaptobrevin‐2 trafficking (Gordon and Cousin 2013; Gordon et al. 2016; Gordon et al. 2011; Kokotos et al. 2019; Koo et al. 2015; Rajappa et al. 2016). The reporter itself does not alter synaptic vesicle recycling (Granseth et al. 2006), though different retrieval kinetics have been observed when the pHluorin reporter is attached to distinct synaptic vesicle proteins (Voglmaier et al. 2006; Pan et al. 2015), likely reflecting differences in trafficking mechanisms and targeting to diverse endocytic pathways (Mori and Takamori 2017; Gan and Watanabe 2018). While we (Kokotos et al. 2019) and others (Koo et al. 2015) have shown that this reporter faithfully reflects endogenous synaptobrevin‐2 trafficking, this caveat should be considered when interpreting these observations. What adds weight to our findings is that we observe downstream physiological consequences consistent with impaired synaptobrevin‐2 trafficking in the form of synaptic depression, only when wild‐type (and not KKD > AAA) α‐synuclein is overexpressed.

Our data suggest that a direct interaction between α‐synuclein and synaptobrevin‐2 underlies both the impaired trafficking of synaptobrevin‐2 and knock‐on synaptic depression; these effects are reduced in the presence of KKD > AAA α‐synuclein, which is known to have reduced affinity for synaptobrevin‐2 (Sun et al. 2019) (further validated here via proximity ligation assay, Figure S2). However, we cannot rule out that the observed impacts to synaptobrevin‐2 trafficking may be indirect, and that the KKD > AAA mutation doesn't interfere with other protein–protein interactions. α‐Synuclein is known to be a highly dynamic protein, displaying activity‐dependent translocation out of the centre of synaptic boutons (Fortin et al. 2010). Interestingly, despite being targeted to synapses as effectively as the WT protein, this mutant form of α‐synuclein disperses more readily from the nerve terminal upon neuronal depolarisation and displays reduced post‐stimulus retrieval to synaptic boutons (Figure S5), raising the intriguing possibility that there is a bidirectional relationship between α‐synuclein and synaptobrevin‐2 trafficking.

What is the mechanism by which moderate overexpression of α‐synuclein specifically slows synaptobrevin‐2 retrieval? Synaptophysin has been proposed to capture synaptobrevin‐2 in clusters following the disassembly of the SNARE complex and increase its trafficking to endocytosing vesicles (Gordon et al. 2011; Rajappa et al. 2016). However, α‐synuclein is not acting in direct competition with synaptophysin, as co‐transfection with synaptophysin increased vesicular synaptobrevin‐2 to a similar level irrespective of the presence of excess α‐synuclein (Figure 4B). Additionally, the binding domains of these proteins do not overlap; α‐synuclein binds to the first 30 N‐terminal residues of synaptobrevin‐2 (Burre et al. 2012), while synaptophysin binds to the C‐terminal portion of the SNARE domain of synaptobrevin‐2 (Harper et al. 2021). Therefore, these data suggest that α‐synuclein likely impacts the retrieval of synaptobrevin‐2 in a manner distinct to that of synaptophysin.

We have recently demonstrated that phosphorylation of α‐synuclein at Thr81 controls the retrieval of synaptobrevin‐2 in an activity‐dependent manner, perhaps via controlling the trafficking of synaptobrevin‐2 to different endocytic pathways (Arvell et al. 2026). It may be that synaptophysin controls the recovery of synaptobrevin‐2 from the plasma membrane, while α‐synuclein regulates the trafficking of the protein to different endocytic pathways, which operate under diverse timescales and conditions (Chanaday et al. 2019; Chanaday and Kavalali 2018a; Soykan et al. 2017). In the current work, we show that increasing α‐synuclein expression causes a dramatic slowing in the retrieval of synaptobrevin‐2. However, there is not a complete stalling in retrieval, as by the start of the 4th stimulus train the level of surface synaptobrevin‐2 pHluorin is similar between conditions (Figure S7). Indeed, the fate of the synaptobrevin‐2 remains unknown. It may be that increasing presynaptic α‐synuclein causes synaptobrevin‐2 to be trafficked to endosomes or vesicles that do not replenish the recycling pool, progressively depleting synaptobrevin‐2 on actively cycling vesicles and resulting in presynaptic depression following repeated stimuli.

α‐Synuclein expression levels have been predicted to impact the orientation of membrane‐bound α‐synuclein upon the lipid interface. At low and physiological concentrations of α‐synuclein, the N‐terminal membrane‐associated region (residues 1–95), which assumes a helical conformation upon lipid‐binding, lies flat on the lipid interface with the dynamic C‐terminal tail protruding into the cytoplasm (Fusco et al. 2014; Roeters et al. 2023). However, at a higher concentration, α‐synuclein has been suggested to adopt a more upright conformation wherein a significant portion of the helical N‐terminus in addition to the C‐terminus protrude away from the lipid interface (Roeters et al. 2023). As such, the expression level of α‐synuclein may influence membrane‐bound conformations as well as interactions with binding partners including synaptobrevin‐2.

The scaffold protein intersectin‐1 specifically binds to SNARE complex‐associated synaptobrevin‐2 to facilitate post‐fusion complex clearance from the active zone, allowing for complex dissociation and protein retrieval (Jäpel et al. 2020). Intersectin‐1 binds to the same 30 N‐terminal residues of synaptobrevin‐2 as α‐synuclein (Burre et al. 2010; Jäpel et al. 2020). Knockout (KO) of intersectin‐1 strands SNARE complexes on the plasma membrane, delaying complex dissociation (Jäpel et al. 2020), though it should be noted that this did not lead to a slowing in synaptobrevin‐2 retrieval. Syb2‐pHluorin missing the N‐terminal 30 amino acids displays increased stranding on the plasma membrane (Jäpel et al. 2020), something that we do not observe with increased α‐synuclein, suggesting that this region may indeed host multiple interactions. Alternatively, α‐synuclein may interfere with binding of the adaptor protein AP180/CALM to synaptobrevin‐2; AP180/CALM binds to the N‐terminal region of the SNARE domain around residue M46 (Koo et al. 2011), and knockout of AP180 slows synaptobrevin‐2 retrieval (Koo et al. 2015). The overexpression of α‐synuclein may thus competitively displace intersectin‐1 or AP180, preventing these proteins from effectively performing their functions, slowing synaptobrevin‐2 retrieval.

Indeed, perhaps the concomitant increase in SNARE complex levels observed with increasing α‐synuclein levels (Burre et al. 2010) may have been the result of this form of competitive inhibition, leading to the aberrant accumulation of post‐fusion *cis*‐SNARE complexes. An analysis of the protein content of post‐mortem human frontal cortex tissue showed that PD cortices had lower levels of assembled SNARE complexes compared to control brains, despite having comparable overall levels of the t‐SNARES SNAP‐25 and syntaxin‐1 (Sharma et al. 2012). This was accompanied by a ~30% reduction in soluble α‐synuclein protein content in Parkinson's disease cortical tissue compared to age‐matched controls, which may be the result of either neuronal and synaptic loss (as supported by the reduction of other synaptic proteins such as synaptophysin) or alternatively due to the sequestration of α‐synuclein into aggregates (Sharma et al. 2012). Thus, in addition to overall α‐synuclein expression levels, the ratio of free, functional α‐synuclein to aggregated α‐synuclein may be an important influence on the efficiency of SNARE complex chaperoning and synaptobrevin‐2 trafficking. Indeed, it would be interesting to explore the impact of increasing α‐synuclein in aged neurons, as well as overexpressing mouse α‐synuclein compared to human α‐synuclein.

Interestingly, we found that α‐synuclein overexpression had no effect on exocytosis rate measured using both syb2‐pHluorin and syt1‐pHluorin upon a single stimulus train, contrasting with some previous studies. Reduced peak amplitude and synaptic vesicle exocytosis have been observed in primary hippocampal neurons overexpressing α‐synuclein using other pHluorin reporters (Nemani et al. 2010; Atias et al. 2019; Sun et al. 2019; Wang et al. 2014). Instead, we observed a reduced peak amplitude of stimulus‐evoked syt1‐pHluorin fluorescence accompanied by a trend towards a decrease in exocytic rate only upon application of repetitive stimulus trains. A likely reason for this disparity is that this study was conducted at physiological temperature whilst previous studies have largely been undertaken at room temperature (Atias et al. 2019; Sun et al. 2019; Wang et al. 2014). Temperature is known to strongly influence both the modes and kinetics of endocytosis (reviewed by Gan and Watanabe (2018) and Chanaday and Kavalali (2018b)) as well as the molecular mediators of these pathways (Harper et al. 2021). Here, we show that, at physiological temperature, increased expression of α‐synuclein specifically leads to slowed synaptobrevin‐2 retrieval. Alternatively, mild differences in the electrical field stimulation conditions used between studies (Nemani et al. 2010; Sun et al. 2019; Wang et al. 2014; Atias et al. 2019) may account for the impact to exocytosis only becoming apparent upon repetitive stimulation in this study.

There are approximately 70 copies of synaptobrevin‐2 per vesicle (Takamori et al. 2006). However, only 2–5 SNARE complexes—and thus synaptobrevin‐2 molecules—are required for vesicle fusion (Domanska et al. 2009; Domanska et al. 2010; Hua and Scheller 2001; Sinha et al. 2011); this high copy number is thought to protect against a failure of exocytosis caused by mild perturbation in synaptobrevin‐2 trafficking (Gordon and Cousin 2016; Gordon and Cousin 2014). It is thus unsurprising that impacts to exocytosis are only apparent following repeated stimulus trains, as has been observed in other systems where synaptobrevin‐2 retrieval is impaired, such as in neurons lacking synaptophysin (Gordon et al. 2011; Kokotos et al. 2019). Impaired synaptobrevin‐2 trafficking would thus be expected to lead to changes in synaptic function over time. Young adult (12–16 weeks) synaptobrevin‐2 heterozygous KO (synaptobrevin‐2^+/−^) mice have normal cue‐and context‐dependent fear memory (Monteggia et al. 2019), but 1 year‐old mice have impaired long‐term potentiation and reduced synaptic vesicle fusion rates, with concomitant impairment of learning and memory in the radial arm water maze which further worsens by 2 years of age (Orock et al. 2020). Notably, synaptobrevin‐2 expression decreased with age in these mice, suggesting age‐dependent decline in synaptobrevin‐2 is linked to cognitive impairment (Orock et al. 2020). Future work should focus on how impaired synaptobrevin‐2 retrieval that occurs as a result of increased α‐synuclein expression could affect synaptic function and neuronal health over the lifespan. This has the potential to inform our understanding of the synaptic dysfunction that arises during PD.

## Author Contributions

**Elyas H. Arvell:** conceptualization, data curation, formal analysis, visualization, investigation, writing – original draft, writing – review and editing. **James P. Carroll:** investigation, writing – review and editing, formal analysis, writing – original draft, data curation. **Joshua Saito:** investigation, formal analysis, writing – review and editing. **Holly Melland:** investigation, writing – review and editing, formal analysis. **Ranjith R. Unnithan:** writing – review and editing, funding acquisition, resources. **Chris French:** funding acquisition, resources, writing – review and editing. **Danny M. Hatters:** writing – review and editing, supervision, resources, funding acquisition. **Sarah L. Gordon:** conceptualization, funding acquisition, writing – original draft, writing – review and editing, supervision, data curation, project administration.

## Funding

This work was supported by National Health and Medical Research Council (NHMRC) Ideas Grant (2003710) and a Florey Fellowship to S.L.G. E.H.A., J.P.C. and H.M. were supported by an Australian Government Research Training Program Scholarship. The Florey Institute of Neuroscience and Mental Health acknowledges the strong support from the Victorian Government and in particular the funding from the Operational Infrastructure Support Grant.

## Conflicts of Interest

The authors declare no conflicts of interest.

## Supporting information


**Figure S1:** Expression and synaptic localisation of α‐synuclein in neurons transfected with 2 or 3 constructs. Hippocampal neurons were co‐transfected with either an empty vector (WT, denoted by –) or α synuclein (+α‐syn, denoted by +) and syb2‐pHluorin for the 2‐construct transfection, and an additional mCerulean construct for the 3‐construct transfection, then fixed and immunolabelled for GFP to identify transfected neurites and α‐syn. (A) Relative expression of α‐syn calculated as a mean ratio of the fluorescence intensity (F) of transfected to un‐transfected nerve terminals with background subtracted. ****p < 0.0001 for WT/2 construct versus +α‐syn/2‐constructs, and WT/3‐construct versus +α‐syn/3‐construct, *p* = 0.3743 for +α‐syn/2 construct versus +α‐syn/3‐construct, *p* > 0.9999 for WT/2‐construct versus WT/3‐construct, one‐way ANOVA with Sidak's multiple comparisons post hoc test comparing selected conditions. (B, C) α‐synuclein synaptic localisation calculated as a mean ratio of the fluorescence intensity (F) of synaptic ROIs (identified by staining for endogenous syb2) over axonal ROIs for 2‐construct (B) and 3‐construct (C) transfection. *p* = 0.0066 for WT () versus +α‐syn (2‐constructs, B), and *p* = 0.0044 WT (−) versus +α‐syn (3‐construct, C), Unpaired *t*‐tests with Welch's correction. Plots show individual *n* and mean ± SEM, *n* = 7 (WT/2‐construct, α‐syn/3‐construct) or 8 (α‐syn/2 construct, WT/3‐construct) individual coverslips. Note that (A) 3‐construct data is also displayed in Figure 1.
**Figure S2:** α‐synucleinAAA displays reduced interaction with synaptobrevin‐2 via proximity ligation assay. HEK293T cells were co‐transfected with syb2‐FLAG and either WT α‐synuclein‐GFP (α‐synWT) or α‐synuclein‐GFP where amino acids 96–96 (KKD) are mutated to alanine (AAA; α‐synAAA). (A) Representative images of α‐syn‐GFP and syb2‐FLAG expression in HEK293 cells. Scale bar = 20 μm. (B) Representative anti FLAG, anti‐GFP and anti‐β‐actin immunoblots. See Figure S5 for uncropped immunoblots. (C) syb2‐FLAG expression. syb2‐FLAG band intensity normalised to stain free total protein blot. *p* = 0.7276 paired *t*‐test. (D) α syn‐GFP expression. α‐syn‐GFP band intensity normalised to stain free total protein blot. *p* = 0.6877 paired *t*‐test. (E) Cells were fixed and proximity ligation assay (PLA) was performed using mouse anti‐FLAG and rabbit anti‐GFP primary antibodies. Single‐primary antibody controls (no FLAG ab or no GFP ab) produced minimal PLA signal. Representative PLA images. Scale bar = 20 μm. (F) Average α‐syn‐GFP expression (arbitrary units). One‐way ANOVA with Tukey's multiple‐comparisons test; no significant differences were detected between groups (*p* > 0.05). (G) Quantification of PLA puncta normalised to α‐synuclein‐GFP‐positive cell area (PLA puncta/mm2). Each data point represents the mean of five fields of view from a single coverslip (*n* = 3 independent coverslips per condition). *p* = 0.0201 for α‐synWT versus α‐synAAA unpaired two‐tailed Student's *t*‐test. Plot shows individual *n* and mean ± SEM. * indicate *p* < 0.05.
**Figure S3:** Uncropped Blots of α‐syn‐GFP and syb2‐FLAG expression in HEK293 cells. HEK293T cells were co‐transfected with syb2‐FLAG and either WT α‐synuclein‐GFP (α‐synWT) or α‐synuclein‐GFP where amino acids 96–96 (KKD) are mutated to alanine (AAA; α‐synAAA). (A, C) Stain‐free total protein blots. (B) α syn‐GFP was detected using an anti‐GFP antibody and (D) syb2‐FLAG was detected using an anti‐FLAG antibody. (C) Following detection of α‐synuclein‐GFP, the membrane was stripped and reprobed for β‐actin as a loading control. Uncropped blots B, D and E correspond to.
**Figure S4:** Relative expression of α‐synucleinAAA. Hippocampal neurons were co‐transfected with syb2‐pHluorin and either an empty vector (WT), WT α‐synuclein (+α‐synWT) or α‐synuclein where amino acids 96–96 (KKD) are mutated to alanine (AAA; +α‐synAAA), then fixed and immunolabelled for GFP to identify transfected neurites and α‐synuclein to examine protein levels. Note that the anti‐α‐synuclein antibody used in Figure S1 did not recognise α‐synAAA, and so instead an in‐house antibody (polyclonal rabbit antibody raised against residues 1–96 of α‐synuclein) was used. Plot shows relative expression of α‐syn calculated as a mean ratio of the fluorescence intensity (F) of transfected to un‐transfected nerve terminals with background subtracted. *p* = 0.0285 for WT versus + α‐synWT, *p* = 0.0123 for WT versus + α‐synAAA, *p* = 0.894 for +α‐synWT versus + α‐synAAA, one‐way ANOVA with Tukey's multiple comparisons post hoc test. Plot shows individual *n* and mean ± SEM α syn expression level, *n* = 4 individual coverslips, * indicate *p* < 0.05.
**Figure S5:** α‐synucleinAAA displays similar steady state levels at synapses and localises to synapses with similar efficacy as WT but displays altered activity‐dependent movement out of synapses. α‐synKO hippocampal neurons co‐transfected with mCherry and α‐syn‐GFP (either α‐synWT or α‐synAAA) were stimulated with a train of 300 action potentials (10 Hz, 35 mA, indicated by labelled black bar) and the change in α‐synGFP fluorescence over time was measured. (A) Representative images of α‐synGFP (either α‐synWT or α‐synAAA). Scale bar = 20 μm. (B) Steady state levels of KKD>AAA and WT α‐synuclein are similar, as revealed by α‐synGFP fluorescence (arbitrary units). *p* = 0.1490 for α‐synWT versus α‐synAAA, unpaired two‐tailed Student's *t*‐test. (C) Coefficient of variation (CV) analysis was used to determine the distribution of α‐synGFP along axonal segments. *p* = 0.9307 for α‐synWT versus α‐synAAA, Mann Whitney test. (D) Time course of mean ΔF/F0 ± SEM of α‐synGFP fluorescence. (E) ΔF/F0 after 300 action potentials (AP). *p* = 0.0150 for α‐synWT versus α‐synAAA, unpaired two‐tailed t‐test. (F) Time course of mean ΔF/F0 ± SEM of α‐synGFP fluorescence normalised to peak amplitude during stimulation. (G) Mean % recovery of α‐synGFP fluorescence to baseline fluorescence. *p* = 0.0043 for α‐synWT versus α synAAA, Mann Whitney test. Plots show individual *n* and mean ± SEM, *n* = 6 (α‐synWT) or 5 (α‐synAAA) individual coverslips from at least 2 independent cultures.
**Figure S6:** Representative pHluorin images for Figures 5 and 6. (A) Representative images relative to Figure 4 of syb2‐pHluorin fluorescence in neutral saline imaging buffer, acid buffer, or alkalising NH4Cl buffer. (B) Representative images relative to Figure 5 of syb2‐pHluorin fluorescence at baseline, after 300AP, at 180 s recovery, and under NH4Cl perfusion. (C) Representative images relative to Figure 6 of syt1‐pHluorin fluorescence from fourth stimulus block at baseline, after 300AP, at 180 s, and under NH4Cl perfusion. Scale bar = 10 μm.
**Figure S7:** Modest α‐synuclein overexpression may induce synaptic depression of syb2 pHluorin retrieval with repeated stimulation. (A) Top: Experimental paradigm. Hippocampal neurons co transfected with syb2‐pHluorin, mCherry (transfection marker), and either an empty vector (WT), α‐synuclein (+α‐synWT) or α‐synuclein with reduced synaptobrevin‐2 binding affinity (+α‐synAAA) were stimulated with trains of 7 ((Arvell et al. 2026)). 300 action potentials (300AP, 10 Hz, 35 mA, indicated by labelled black bar) every 5 min, and the change in syb2‐pHluorin fluorescence over time was measured across the final (fourth) stimulus train (indicated by bar). Neurons were then perfused with NH4Cl to reveal the total syb2‐pHluorin fluorescence, then acid buffer to allow calculation of surface syb2‐pHluorin. Below: Representative images of syb2‐pHluorin fluorescence from fourth stimulus block at baseline, after 300AP, at 180 s, and under NH4Cl perfusion. (B) Time course of mean ΔF/F0 ± SEM of syb2‐pHluorin fluorescence, across the fourth stimulus train, normalised to total pHluorin fluorescence upon NH4Cl. Shading indicates SEM. * *p* < 0.05 for WT versus + asynWT (purple) over time indicated by coloured bars, two‐way repeated measures ANOVA with Dunnet's multiple comparisons post hoc test. (C) Peak amplitude (mean maximum evoked ΔF/F0) of syb2‐pHluorin fluorescence during stimulation, normalised to total fluorescence. *p* = 0.1074 for WT versus + asynWT, *p* = 0.9968 for WT versus +α‐synAAA, one‐way ANOVA with Dunnett's multiple comparisons test. (D) Mean initial rate of fluorescence change calculated from linear fit of ΔF/F0 per second over the first 10 s of stimulation, normalised to total fluorescence. *p* = 0.0955 Kruskal–Wallis test comparing group medians (no distribution assumption) (E) Vesicular fraction of syb2‐pHluorin fluorescence (F) prior to stimulation calculated as: (total−surface fluorescence)/(total−background fluorescence) × 100 F. *p* = 0.9345 for WT versus α‐synWT, *p* = 0.9947 for WT versus α‐synAAA, Ordinary one‐way ANOVA with Dunnett's multiple comparisons test. (F) Coefficient of variation (CV) analysis of total syb2‐pHluorin fluorescence (revealed with NH4 perfusion). *p* = 0.4757 for WT versus α‐synWT, *p* = 0.8093 for WT versus α‐synAAA, Ordinary one‐way ANOVA with Dunnett's multiple comparisons test. Plots show individual *n* and mean ± SEM, *n* = 7 (WT), 8 (α‐synWT) or 7 (α synAAA) individual coverslips from at least 3 independent cultures.
**Table S1:** Summary of statistics.

## Data Availability

Data and custom‐made materials are available upon reasonable request to the corresponding author, with a signed data access agreement.
