# Supplementary material for: Increased Expression of α‐Synuclein Impairs Trafficking of the Essential SNARE Protein Synaptobrevin‐2
Source: J Neurochem. 2026 Oct 8;170(10):e70568. doi: 10.1111/jnc.70568 (PMC13648009; doi:10.1111/jnc.70568)
Supplement: Supplementary file 1 [file JNC-170-e70568-s001.pdf]

Increased expression of α-synuclein impairs trafficking of the essential SNARE protein synaptobrevin-2

Elyas H. Arvell#, James P. Carroll#, Joshua Saito, Holly Melland, Ranjith R. Unnithan, Chris French, Danny M. Hatters, Sarah L. Gordon*

SUPPLEMENTARY MATERIAL

Supplementary Figure 1

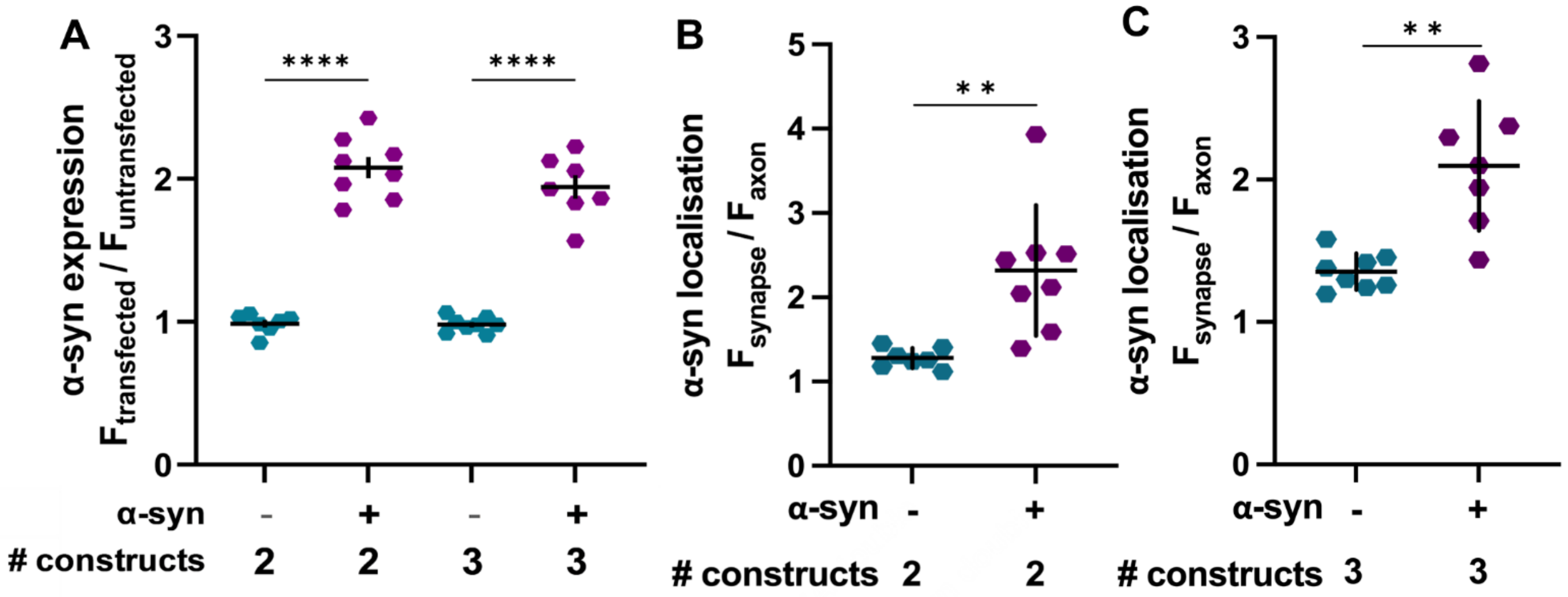

Supplementary Figure 1. Expression and synaptic localisation of α-synuclein in neurons transfected with 2 or 3 constructs. Hippocampal neurons were co-transfected with either an empty vector (WT, denoted by –) or α-synuclein (+α-syn, denoted by +) and syb2-pHluorin for the 2-construct transfection, and an additional mCerulean construct for the 3-construct transfection, then fixed and immunolabelled for GFP to identify transfected neurites and α-syn. A) Relative expression of α-syn calculated as a mean ratio of the fluorescence intensity (F) of transfected to un-transfected nerve terminals with background subtracted. **** $P < 0.0001$ for WT/2 construct vs. +α-syn/2-constructs, and WT/3-construct vs. +α-syn/3-construct, $P = 0.3743$ for +α-syn/2-construct vs. +α-syn/3-construct, $P > 0.9999$ for WT/2-construct vs. WT/3-construct, one-way ANOVA with Sidak's multiple comparisons post-hoc test comparing selected conditions. B,C) α-synuclein synaptic localisation calculated as a mean ratio of the fluorescence intensity (F) of synaptic ROIs (identified by staining for endogenous syb2) over axonal ROIs for 2-construct (B) and 3-construct (C) transfection. $P = 0.0066$ for WT (-) vs. +α-syn (2-constructs, B), and $P = 0.0044$ WT (-) vs. +α-syn (3-construct, C), Unpaired t tests with Welch's correction. Plots show individual *n* and mean ± SEM, $n = 7$ (WT/2-construct, α-syn/3-construct) or 8 (α-syn/2-construct, WT/3-construct) individual coverslips. Note that A) 3-construct data is also displayed in Figure 1.

Supplementary Figure 2

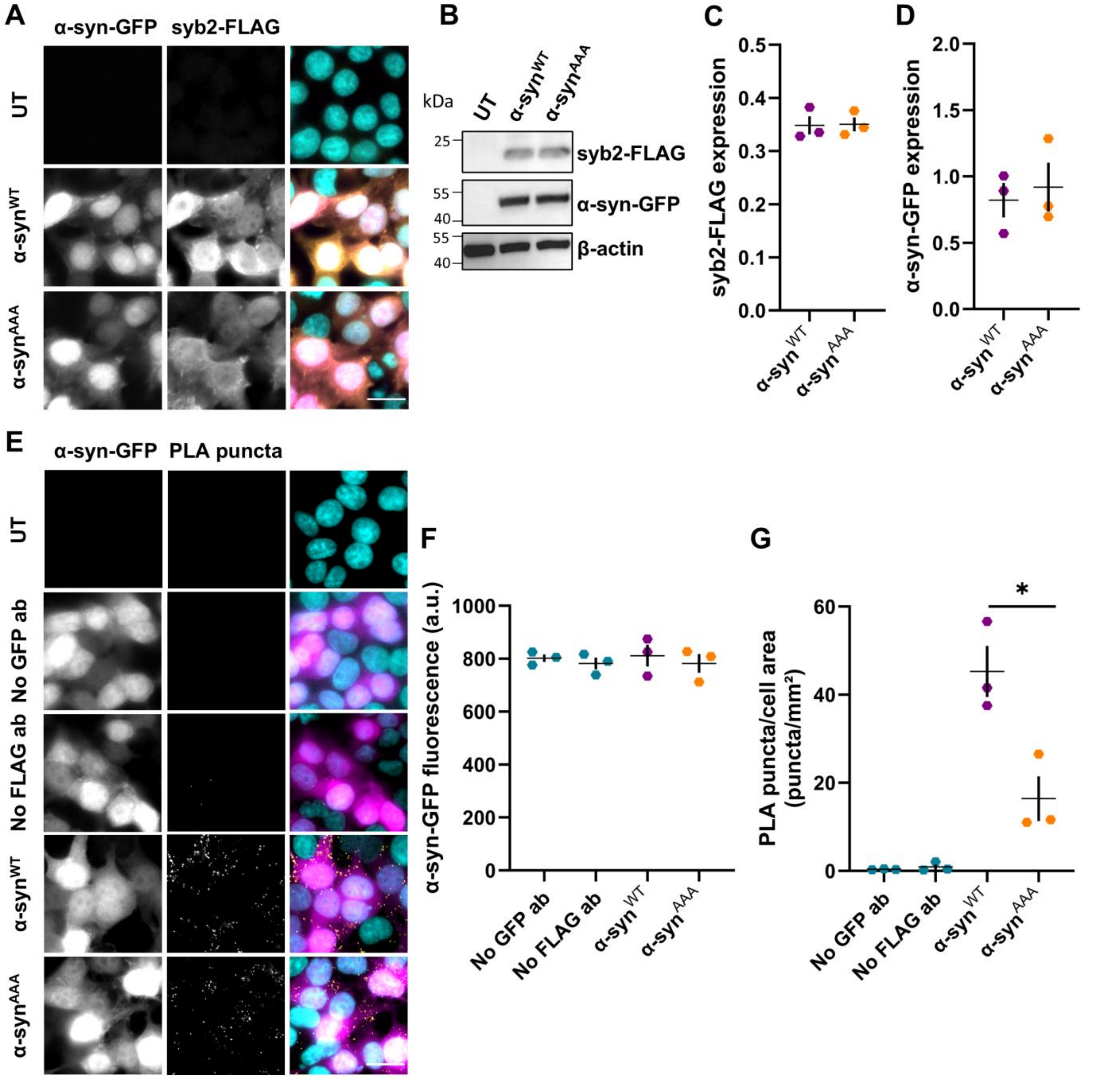

Supplementary Figure 2. α-synuclein^{AAA} displays reduced interaction with synaptobrevin-2 via proximity ligation assay. HEK293T cells were co-transfected with syb2-FLAG and either WT α-synuclein-GFP (α-syn^{WT}) or α-synuclein-GFP where amino acids 96-96 (KKD) are mutated to alanine (AAA; α-syn^{AAA}). (A) Representative images of α-syn-GFP and syb2-FLAG expression in HEK293 cells. Scale bar= 20 µm. (B) Representative anti-FLAG, anti-GFP, and anti-β-actin immunoblots. See Supp Fig 5 for uncropped immunoblots. (C) syb2-FLAG expression. syb2-FLAG band intensity normalised to stain free total protein blot. *P* = 0.7276 paired t test. (D) α-syn-GFP expression. α-syn-GFP band intensity normalised to stain free total protein blot. *P* = 0.6877 paired t test. (E) Cells were fixed and proximity ligation assay (PLA) was performed using mouse anti-FLAG and rabbit anti-GFP primary antibodies. Single-primary antibody controls (no FLAG ab or no GFP ab) produced minimal PLA signal. Representative PLA images. Scale bar= 20 µm. (F) Average α-syn-GFP expression (arbitrary units). One-way ANOVA with Tukey's multiple-comparisons test; no significant differences were detected between groups (P > 0.05). (G) Quantification of PLA puncta normalised to α-synuclein-GFP-positive cell area (PLA puncta/mm^2). Each data point represents the mean of five fields of view from a single coverslip (n = 3 independent coverslips per condition). *P* = 0.0201 for α-syn^{WT} vs α-syn^{AAA} unpaired two-tailed Student's t-test. Plot shows individual *n* and mean ± SEM. * indicate *P* <0.05.

Supplementary Figure 3

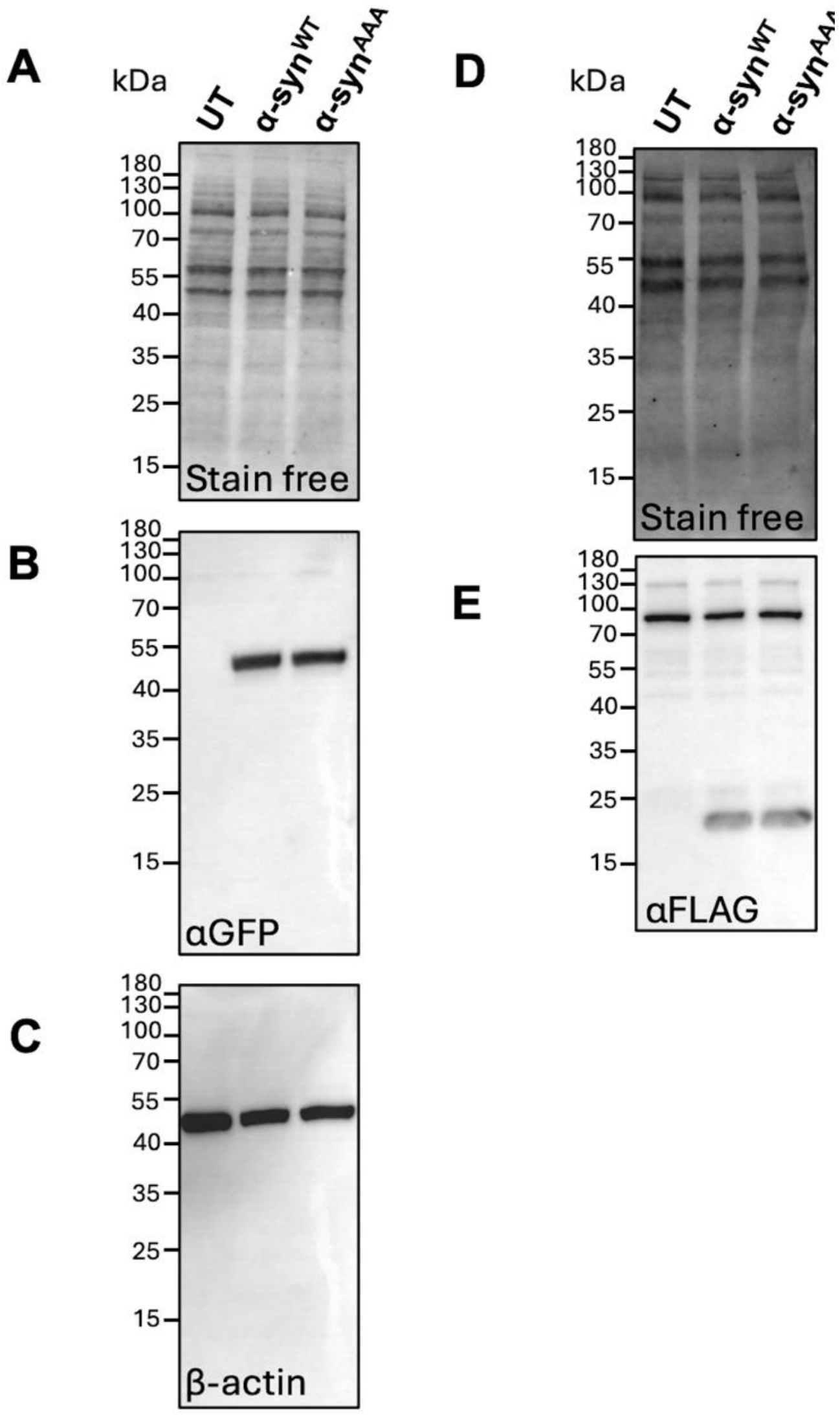

Supplementary Figure 3. Uncropped Blots of α-syn-GFP and syb2-FLAG expression in HEK293 cells. HEK293T cells were co-transfected with syb2-FLAG and either WT α-synuclein-GFP (α-syn^{WT}) or α-synuclein-GFP where amino acids 96-96 (KKD) are mutated to alanine (AAA; α-syn^{AAA}). (A, C) Stain-free total protein blots. (B) α-syn-GFP was detected using an anti-GFP antibody and (D) syb2-FLAG was detected using an anti-FLAG antibody. (C) Following detection of α-synuclein-GFP, the membrane was stripped and reprobed for β-actin as a loading control. Uncropped blots B, D and E correspond to Supplementary Figure 3B.

Supplementary Figure 4

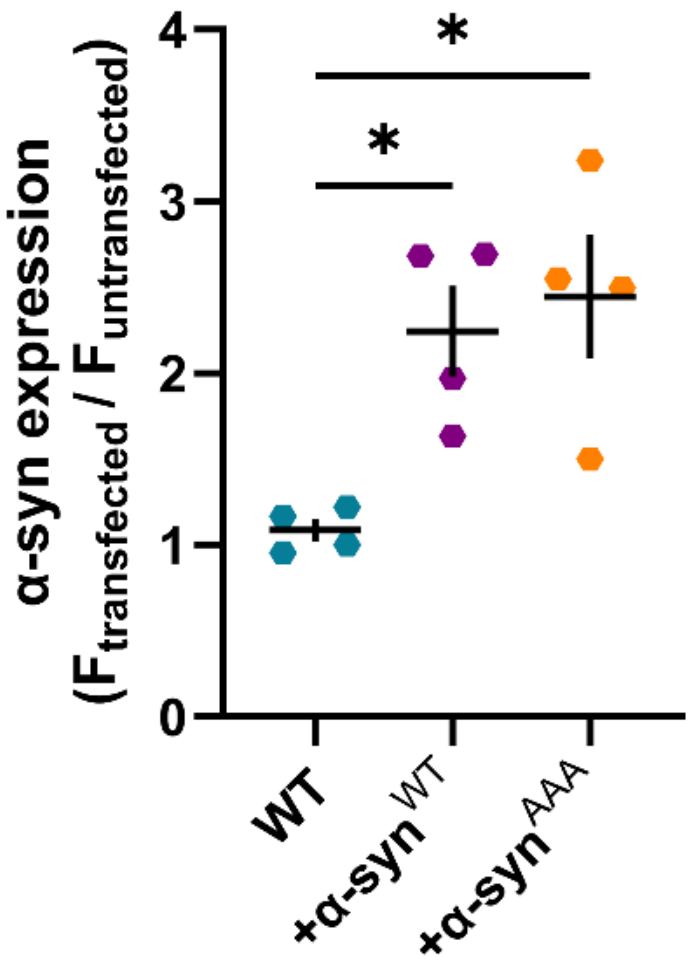

Supplementary Figure 4. Relative expression of α-synuclein^{AAA}. Hippocampal neurons were co-transfected with syb2-pHluorin and either an empty vector (WT), WT α-synuclein (+α-syn^{WT}) or α-synuclein where amino acids 96-96 (KKD) are mutated to alanine (AAA; +α-syn^{AAA}), then fixed and immunolabelled for GFP to identify transfected neurites and α-synuclein to examine protein levels. Note that the anti-α-synuclein antibody used in Supp Fig1. did not recognise α-syn^{AAA}, and so instead an in-house antibody (polyclonal rabbit antibody raised against residues 1-96 of α-synuclein) was used. Plot shows relative expression of α-syn calculated as a mean ratio of the fluorescence intensity (F) of transfected to un-transfected nerve terminals with background subtracted. *P* = 0.0285 for WT vs + α-syn^{WT}, *P* = 0.0123 for WT vs +α-syn^{AAA}, *P* = 0.894 for +α-syn^{WT} vs +α-syn^{AAA}, one-way ANOVA with Tukey's multiple comparisons post-hoc test. Plot shows individual *n* and mean ± SEM α-syn expression level, *n* = 4 individual coverslips, * indicate *P* <0.05.

Supplementary Figure 5

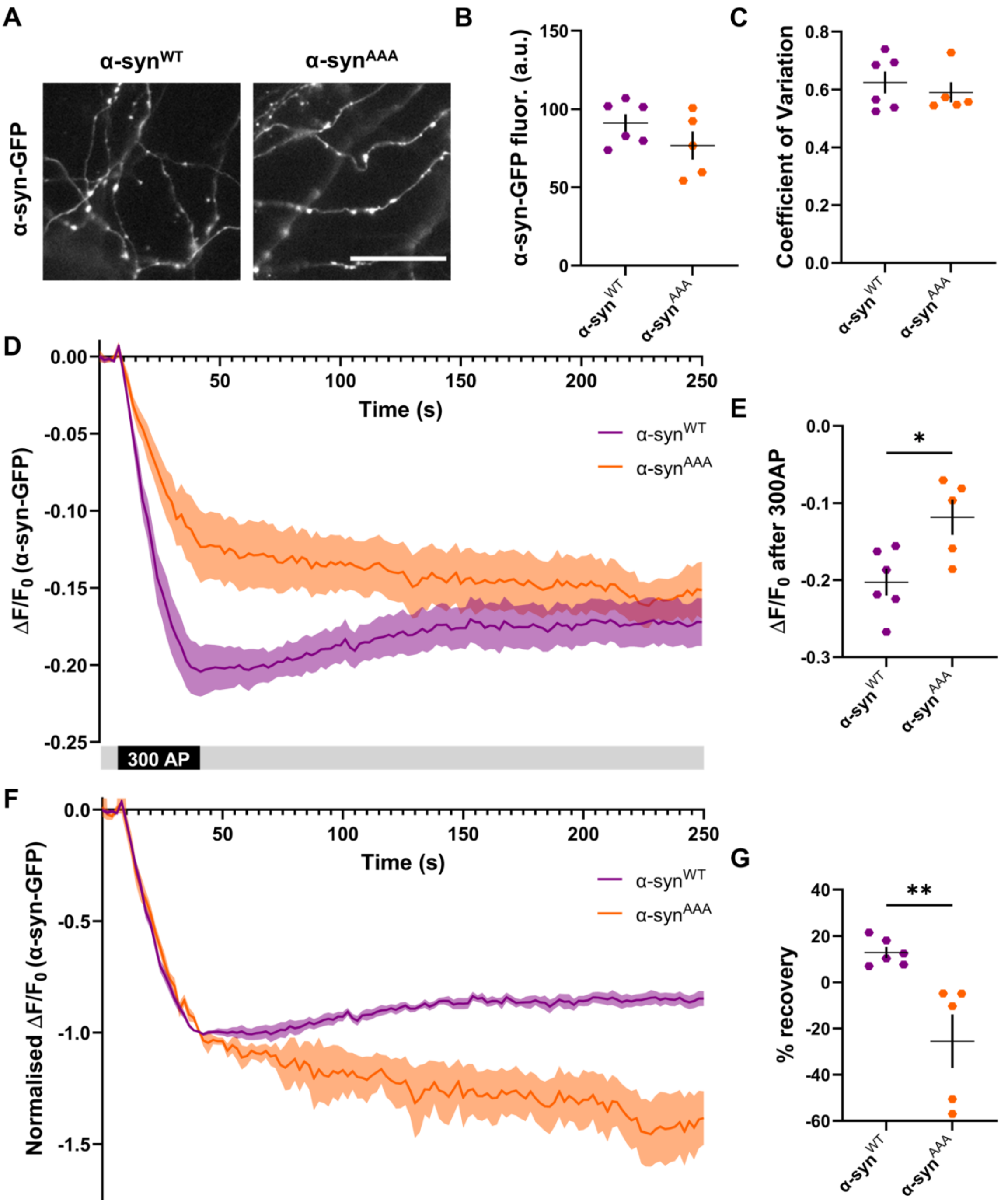

Supplementary Figure 5. α-synuclein^AAA displays similar steady state levels at synapses and localises to synapses with similar efficacy as WT but displays altered activity-dependent movement out of synapses. α-syn^KO hippocampal neurons co-transfected with mCherry and α-syn-GFP (either α-syn^WT or α-syn^AAA) were stimulated with a train of 300 action potentials (10 Hz, 35 mA, indicated by labelled black bar) and the change in α-syn^GFP fluorescence over time was measured. (A) Representative images of α-syn^GFP (either α-syn^WT or α-syn^AAA). Scale bar= 20 µm. (B) Steady state levels of KKD>AAA and WT α-synuclein are similar, as revealed by α-syn^GFP fluorescence (arbitrary units). *P* = 0.1490 for α-syn^WT vs α-syn^AAA, unpaired two-tailed Student's t-test. (C) Coefficient of variation (CV) analysis was used to determine the distribution of α-syn^GFP along axonal segments. *P* = 0.9307 for α-syn^WT vs α-syn^AAA, Mann Whitney test. (D) Time course of mean $\Delta F/F_0$ ± SEM of α-syn^GFP fluorescence. (E) $\Delta F/F_0$ after 300 action potentials (AP). *P* = 0.0150 for α-syn^WT vs α-syn^AAA, unpaired two-tailed t test. (F) Time course of mean $\Delta F/F_0$ ± SEM of α-syn^GFP fluorescence normalised to peak amplitude during stimulation. (G) Mean % recovery of α-syn^GFP fluorescence to baseline fluorescence. *P* = 0.0043 for α-syn^WT vs α-syn^AAA, Mann Whitney test. Plots show individual *n* and mean ± SEM, *n* = 6 (α-syn^WT) or 5 (α-syn^AAA) individual coverslips from at least 2 independent cultures.

Supplementary Figure 6

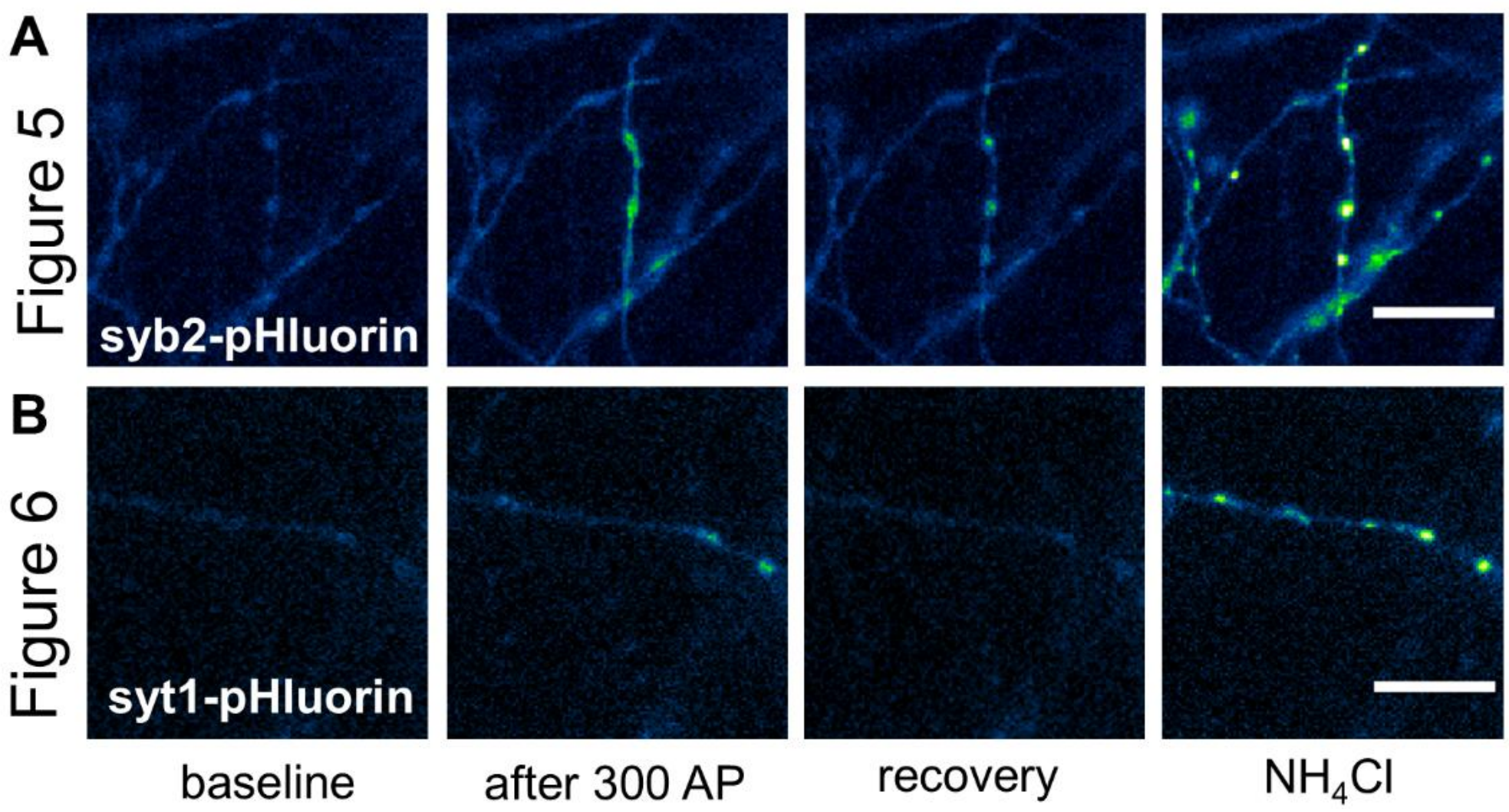

Supplementary Figure 6. Representative pHluorin images for Figures 5 and 6. A) Representative images relative to Figure 4 of syb2-pHluorin fluorescence in neutral saline imaging buffer, acid buffer, or alkalising NH_4Cl buffer. B) Representative images relative to Figure 5 of syb2-pHluorin fluorescence at baseline, after 300AP, at 180s (recovery, and under NH_4Cl perfusion. C) Representative images relative to Figure 6 of syt1-pHluorin fluorescence from fourth stimulus block at baseline, after 300AP, at 180s, and under NH_4Cl perfusion. Scale bar = 10µm.

Supplementary Figure 7

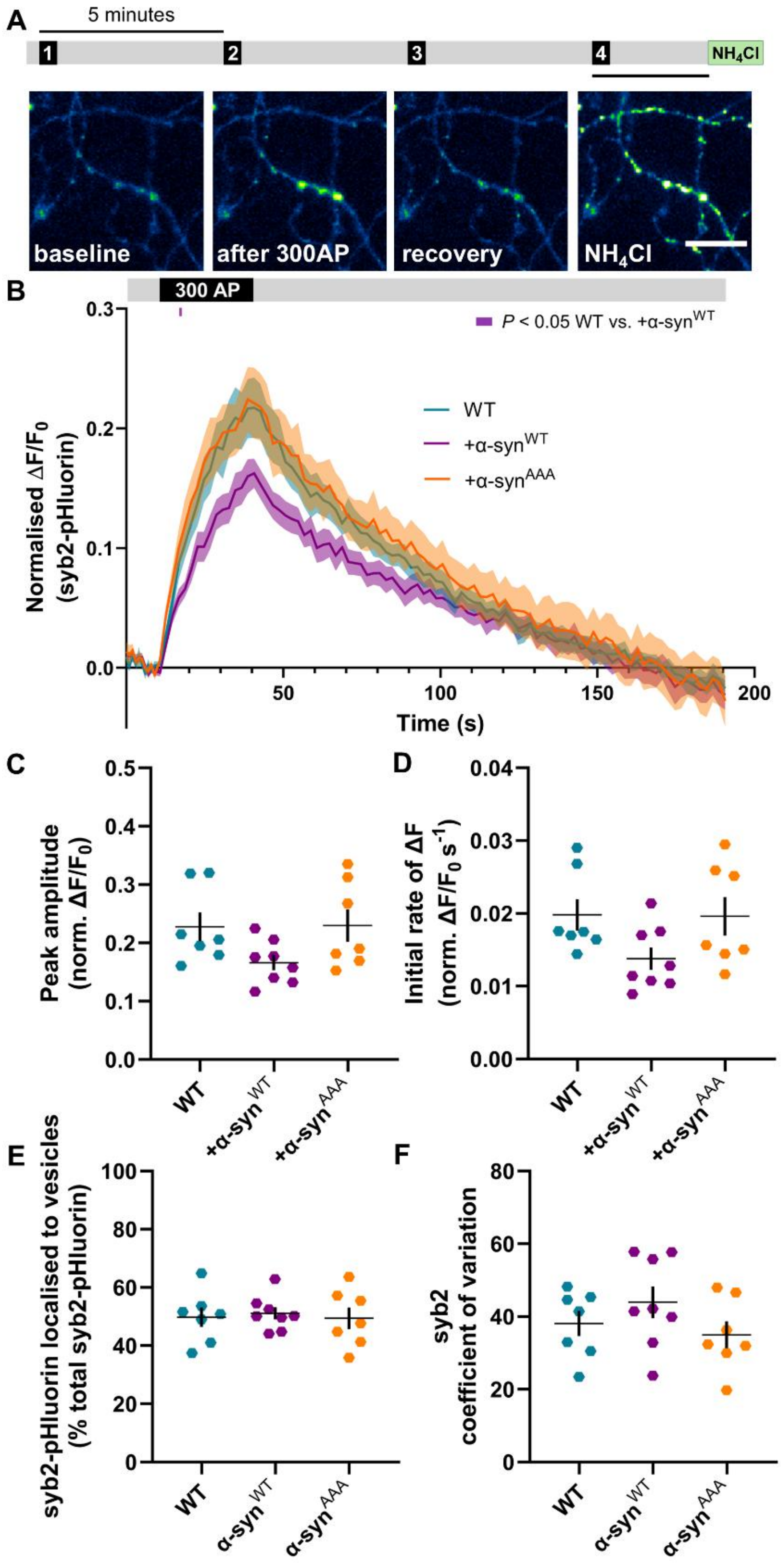

Supplementary Figure 7. Modest α-synuclein overexpression may induce synaptic depression of syb2-pHluorin retrieval with repeated stimulation. (A) Top: Experimental paradigm. Hippocampal neurons co-transfected with syb2-pHluorin, mCherry (transfection marker), and either an empty vector (WT), α-synuclein (+α-syn^{WT}) or α-synuclein with reduced synaptobrevin-2 binding affinity (+α-syn^{AAA}) were stimulated with trains of

300 action potentials (300AP, 10 Hz, 35 mA, indicated by labelled black bar) every 5 minutes, and the change in syb2-pHluorin fluorescence over time was measured across the final (fourth) stimulus train (indicated by bar). Neurons were then perfused with NH_4Cl to reveal the total syb2-pHluorin fluorescence, then acid buffer to allow calculation of surface syb2-pHluorin. Below: Representative images of syb2-pHluorin fluorescence from fourth stimulus block at baseline, after 300AP, at 180s, and under NH_4Cl perfusion. (B) Time course of mean $\Delta F/F_0$ ± SEM of syb2-pHluorin fluorescence, across the fourth stimulus train, normalised to total pHluorin fluorescence upon NH_4Cl. Shading indicates SEM. * $P < 0.05$ for WT vs +asyn^{WT} (purple) over time indicated by coloured bars, two-way repeated measures ANOVA with Dunnet's multiple comparisons post-hoc test. (C) Peak amplitude (mean maximum evoked $\Delta F/F_0$) of syb2-pHluorin fluorescence during stimulation, normalised to total fluorescence. $P = 0.1074$ for WT vs +asyn^{WT}, $P = 0.9968$ for WT versus +α-syn^{AAA}, one-way ANOVA with Dunnett's multiple comparisons test. (D) Mean initial rate of fluorescence change calculated from linear fit of $\Delta F/F_0$ per second over the first 10 s of stimulation, normalised to total fluorescence. $P = 0.0955$ Kruskal-Wallis test comparing group medians (no distribution assumption) (E) Vesicular fraction of syb2-pHluorin fluorescence (F) prior to stimulation calculated as: (total – surface fluorescence)/(total – background fluorescence) × 100 F). $P = 0.9345$ for WT vs α-syn^{WT}, $P = 0.9947$ for WT vs α-syn^{AAA}, Ordinary one-way ANOVA with Dunnett's multiple comparisons test. (F) Coefficient of variation (CV) analysis of total syb2-pHluorin fluorescence (revealed with NH4 perfusion). $P = 0.4757$ for WT vs α-syn^{WT}, $P = 0.8093$ for WT vs α-syn^{AAA}, Ordinary one-way ANOVA with Dunnett's multiple comparisons test. Plots show individual n and mean ± SEM, $n = 7$ (WT), 8 (α-syn^{WT}) or 7 (α-syn^{AAA}) individual coverslips from at least 3 independent cultures.

SUPPLEMENTARY TABLE 1: SUMMARY OF STATISTICS

Fig.	Normal by Shapiro-Wilk test?	Test	Post-test comparisons
1D	Yes	Unpaired two-tailed Student's t-test	NA
1E	Yes	Unpaired two-tailed Student's t-test	NA
1F	Yes	Unpaired two-tailed Student's t-test	NA
1H	Yes	Unpaired two-tailed Student's t-test	NA
1J	Yes	Unpaired two-tailed Student's t-test	NA
2C	Yes	Unpaired two-tailed Student's t-test	NA
2D	NA	Two-way repeated measures ANOVA	Sidak's
2E	Yes	Unpaired two-tailed Student's t-test	NA
2F	Yes	Unpaired two-tailed Student's t-test	NA
3B	NA	Two-way repeated measures ANOVA	Sidak's
3C	Yes	Unpaired two-tailed Student's t-test	NA
3D	Yes	Unpaired two-tailed Student's t-test	NA
3E	Yes	Unpaired two-tailed Student's t-test	NA
3F	Yes	Unpaired two-tailed Student's t-test	NA
4B	Yes	One-way ANOVA	Dunnett's
5B	NA	Mixed effects analysis	Sidak's
5C	Yes	One-way ANOVA	Dunnett's
5D	Yes	One-way ANOVA	Dunnett's
5E	Yes	One-way ANOVA	Dunnett's
6B	NA	Two-way repeated measures ANOVA	Dunnett's
6C	Yes	One-way ANOVA	Dunnett's
6D	Yes	One-way ANOVA	Dunnett's
SF1A	Yes	One-way ANOVA	Sidak's
SF1B	Yes	Unpaired two-tailed Student's t-test	NA
SF1C	Yes	Unpaired two-tailed Student's t-test	NA
SF2C	Yes	Paired two-tailed t-test	NA
SF2D	Yes	Paired two-tailed t-test	NA
SF2F	Yes	One-way ANOVA	Tukey's
SF2G	Yes	Unpaired two-tailed Student's t-test	NA
SF4	Yes	One-way ANOVA	Tukey's
SF5B	Yes	Unpaired t test	NA
SF5C	No	Mann Whitney test	NA
SF5E	Yes	Unpaired t test	NA
SF5G	No	Mann Whitney test	NA
SF7B	NA	Two-way repeated measures ANOVA	Dunnett's
SF7C	Yes	One-way ANOVA	Dunnett's
SF7D	No	Kruskal-Wallis test	NA
SF7E	Yes	One-way ANOVA	Dunnett's
SF7F	Yes	One-way ANOVA	Dunnett's

**FIGURE 1D**	WT	+α-syn
Number of values	8	7
Mean	0.9803	1.943
Std. Deviation	0.05306	0.2187
Std. Error of Mean	0.01876	0.08267
	WT	+α-syn
;		
Shapiro-Wilk test		
W	0.9651	0.9683
P value	0.8568	0.8861
Passed normality test (alpha=0.05)?	Yes	Yes
P value summary	ns	ns
Column B	+α-syn	
vs.	vs.	
Column A	WT	
Unpaired t test		
P value	<0.0001	
P value summary	****	
Significantly different (P < 0.05)?	Yes	
One- or two-tailed P value?	Two-tailed	
t, df	t=12.11, df=13	
How big is the difference?		
Mean of column A	0.9803	
Mean of column B	1.943	
Difference between means (B - A) ± SEM	0.9625 ± 0.07950	
95% confidence interval	0.7908 to 1.134	
R squared (eta squared)	0.9185	
F test to compare variances		
F, DFn, Dfd	16.99, 6, 7	
P value	0.0015	
P value summary	**	
Significantly different (P < 0.05)?	Yes	
Data analyzed		
Sample size, column A	8	
Sample size, column B	7	

**FIGURE 1E**	WT	+α-syn
Number of values	9	9
Mean	1.761	1.576
Std. Deviation	0.2853	0.1387
Std. Error of Mean	0.09511	0.04624
	WT	+α-syn
Test for normal distribution		
Shapiro-Wilk test		
W	0.9048	0.9614
P value	0.2813	0.8127
Passed normality test (alpha=0.05)?	Yes	Yes
P value summary	ns	ns
Column B	+α-syn	
vs.	vs.	
Column A	WT	
Unpaired t test		
P value	0.098	
P value summary	ns	
Significantly different (P < 0.05)?	No	
One- or two-tailed P value?	Two-tailed	
t, df	t=1.757, df=16	
How big is the difference?		
Mean of column A	1.761	
Mean of column B	1.576	
Difference between means (B - A) ± SEM	-0.1858 ± 0.1058	
95% confidence interval	-0.4100 to 0.03839	
R squared (eta squared)	0.1617	
F test to compare variances		
F, DFn, Dfd	4.230, 8, 8	
P value	0.0571	
P value summary	ns	
Significantly different (P < 0.05)?	No	
Data analyzed		
Sample size, column A	9	
Sample size, column B	9	

**FIGURE 1F**	WT	+α-syn
Number of values	9	9
Mean	2.028	1.824
Std. Deviation	0.3663	0.2138
Std. Error of Mean	0.1221	0.07128
	WT	+α-syn
Test for normal distribution		
Shapiro-Wilk test		
W	0.9403	0.9351
P value	0.5849	0.5314
Passed normality test (alpha=0.05)?	Yes	Yes
P value summary	ns	ns
Column B	+α-syn	
vs.	vs.	
Column A	WT	
Unpaired t test		
P value	0.1678	
P value summary	ns	
Significantly different (P < 0.05)?	No	
One- or two-tailed P value?	Two-tailed	
t, df	t=1.445, df=16	
How big is the difference?		
Mean of column A	2.028	
Mean of column B	1.824	
Difference between means (B - A) ± SEM	-0.2043 ± 0.1414	
95% confidence interval	-0.5040 to 0.09545	
R squared (eta squared)	0.1154	
F test to compare variances		
F, DFn, Dfd	2.934, 8, 8	
P value	0.1489	
P value summary	ns	
Significantly different (P < 0.05)?	No	
Data analyzed		
Sample size, column A	9	
Sample size, column B	9	

**FIGURE 1H**	WT	+α-syn
Number of values	9	9
Mean	42.2	42.71
Std. Deviation	7.616	3.053
Std. Error of Mean	2.539	1.018
	WT	+α-syn
Test for normal distribution		
Shapiro-Wilk test		
W	0.8623	0.8725
P value	0.1016	0.1307
Passed normality test (alpha=0.05)?	Yes	Yes
P value summary	ns	ns
Column B	+α-syn	
vs.	vs.	
Column A	WT	
Unpaired t test		
P value	0.8533	
P value summary	ns	
Significantly different (P < 0.05)?	No	
One- or two-tailed P value?	Two-tailed	
t, df	t=0.1880, df=16	
How big is the difference?		
Mean of column A	42.2	
Mean of column B	42.71	
Difference between means (B - A) ± SEM	0.5141 ± 2.735	
95% confidence interval	-5.284 to 6.312	
R squared (eta squared)	0.002203	
F test to compare variances		
F, DFn, Dfd	6.223, 8, 8	
P value	0.0181	
P value summary	*	
Significantly different (P < 0.05)?	Yes	
Data analyzed		
Sample size, column A	9	
Sample size, column B	9	

**FIGURE 1J**		
	WT	+α-syn
Test for normal distribution		
Shapiro-Wilk test		
W	0.951	0.9599
P value	0.701	0.7974
Passed normality test (alpha=0.05)?	Yes	Yes
P value summary	ns	ns
Column B	+α-syn	
vs.	vs.	
Column A	WT	
Unpaired t test		
P value	0.5347	
P value summary	ns	
Significantly different (P < 0.05)?	No	
One- or two-tailed P value?	Two-tailed	
t, df	t=0.6345, df=16	
How big is the difference?		
Mean of column A	43.28	
Mean of column B	45.62	
Difference between means (B - A) ± SEM	2.331 ± 3.674	
95% confidence interval	-5.458 to 10.12	
R squared (eta squared)	0.02454	
F test to compare variances		
F, DFn, Dfd	1.159, 8, 8	
P value	0.8396	
P value summary	ns	
Significantly different (P < 0.05)?	No	
Data analyzed		
Sample size, column A	9	
Sample size, column B	9	

**FIGURE 2C**	WT	+α-syn
Number of values	9	9
Mean	0.3547	0.3707
Std. Deviation	0.07734	0.04838
Std. Error of Mean	0.02578	0.01613
	WT	+α-syn
Test for normal distribution		
Shapiro-Wilk test		
W	0.9444	0.9221
P value	0.6289	0.4099
Passed normality test (alpha=0.05)?	Yes	Yes
P value summary	ns	ns
Column B	+α-syn	
vs.	vs.	
Column A	WT	
Unpaired t test		
P value	0.607	
P value summary	ns	
Significantly different (P < 0.05)?	No	
One- or two-tailed P value?	Two-tailed	
t, df	t=0.5247, df=16	
How big is the difference?		
Mean of column A	0.3547	
Mean of column B	0.3707	
Difference between means (B - A) ± SEM	0.01596 ± 0.03041	
95% confidence interval	-0.04851 to 0.08042	
R squared (eta squared)	0.01692	
F test to compare variances		
F, DFn, Dfd	2.556, 8, 8	
P value	0.206	
P value summary	ns	
Significantly different (P < 0.05)?	No	
Data analyzed		
Sample size, column A	9	
Sample size, column B	9	

**FIGURE 2D**					
Two-way RM ANOVA	Matching: Stacked				
Assume sphericity?	No				
Alpha	0.05				
Source of Variation	% of total variation	P value	P value summary	Significant?	Geisser-Greenhouse's epsilon
Time x Column Factor	2.167	<0.0001	****	Yes	
Time	90.44	<0.0001	****	Yes	0.04396
Column Factor	3.578	0.0001	***	Yes	
Subject	2.183	<0.0001	****	Yes	
ANOVA table	SS	DF	MS	F (DFn, DFd)	P value
Time x Column Factor	2.715	100	0.02715	F (100, 1600) = 21.28	P<0.0001
Time	113.3	100	1.133	F (4.396, 70.34) = 888.0	P<0.0001
Column Factor	4.482	1	4.482	F (1, 16) = 26.22	P=0.0001
Subject	2.734	16	0.1709	F (16, 1600) = 133.9	P<0.0001
Residual	2.041	1600	0.001276		
Difference between column means					
Mean of WT	0.3762				
Mean of α-syn OVX	0.4755				
Difference between means	-0.0993				
SE of difference	0.01939				
95% CI of difference	-0.1404 to -0.05819				
Data summary					
Number of columns (Column Factor)	2				
Number of rows (Time)	101				
Number of subjects (Subject)	18				
Number of missing values	0				

Compare each cell mean with the other cell mean in that row	
Number of families	1
Number of comparisons per family	101
Alpha	0.05

**FIGURE 2D**					
Šídák's multiple comparisons test	Mean Diff.	95.00% CI of diff.	Below threshold?	Summary	Adjusted P Value
WT - α-syn OVX					
Row 1	0.006001	-0.04163 to 0.05363	No	ns	>0.9999
Row 2	0.001155	-0.06132 to 0.06363	No	ns	>0.9999
Row 3	0.007535	-0.03168 to 0.04675	No	ns	>0.9999
Row 4	0.003456	-0.03022 to 0.03713	No	ns	>0.9999
Row 5	0.008288	-0.02393 to 0.04051	No	ns	>0.9999
Row 6	0.006244	-0.02571 to 0.03820	No	ns	>0.9999
Row 7	-0.00617	-0.03443 to 0.02209	No	ns	>0.9999
Row 8	-0.002948	-0.02444 to 0.01854	No	ns	>0.9999
Row 9	0.003594	-0.01306 to 0.02025	No	ns	>0.9999
Row 10	-0.0007195	-0.03120 to 0.02976	No	ns	>0.9999
Row 11	0.01201	-0.02612 to 0.05014	No	ns	>0.9999
Row 12	0.008293	-0.05939 to 0.07598	No	ns	>0.9999
Row 13	0.02097	-0.08019 to 0.1221	No	ns	>0.9999
Row 14	0.02658	-0.1111 to 0.1643	No	ns	>0.9999
Row 15	0.04101	-0.1194 to 0.2014	No	ns	>0.9999
Row 16	0.04986	-0.1335 to 0.2332	No	ns	>0.9999
Row 17	0.05509	-0.1326 to 0.2427	No	ns	>0.9999
Row 18	0.06311	-0.1115 to 0.2377	No	ns	>0.9999
Row 19	0.06927	-0.09347 to 0.2320	No	ns	0.9998
Row 20	0.07865	-0.08084 to 0.2381	No	ns	0.9907
Row 21	0.06927	-0.04996 to 0.1885	No	ns	0.8925
Row 22	0.05032	-0.03611 to 0.1368	No	ns	0.8883
Row 23	0.02308	-0.04242 to 0.08858	No	ns	>0.9999
Row 24	0.02254	-0.02461 to 0.06969	No	ns	0.9955
Row 25	0.002396	-0.01043 to 0.01522	No	ns	>0.9999
Row 26	-0.0168	-0.05404 to 0.02044	No	ns	0.9956
Row 27	-0.04178	-0.09078 to 0.007222	No	ns	0.1722
Row 28	-0.04871	-0.1050 to 0.007549	No	ns	0.1598
Row 29	-0.05911	-0.1354 to 0.01719	No	ns	0.3265
Row 30	-0.06744	-0.1598 to 0.02497	No	ns	0.4356
Row 31	-0.08082	-0.1840 to 0.02236	No	ns	0.2974
Row 32	-0.09677	-0.2113 to 0.01780	No	ns	0.1899
Row 33	-0.0951	-0.2029 to 0.01271	No	ns	0.1375
Row 34	-0.1025	-0.2271 to 0.02208	No	ns	0.2269
Row 35	-0.1052	-0.2276 to 0.01721	No	ns	0.1686
Row 36	-0.1134	-0.2557 to 0.02894	No	ns	0.2799
Row 37	-0.1244	-0.2544 to 0.005676	No	ns	0.0731
Row 38	-0.1249	-0.2596 to 0.009888	No	ns	0.0944
Row 39	-0.1329	-0.2910 to 0.02519	No	ns	0.1899
Row 40	-0.1304	-0.2938 to 0.03299	No	ns	0.2664
Row 41	-0.1203	-0.2687 to 0.02809	No	ns	0.2495
Row 42	-0.1367	-0.2841 to 0.01060	No	ns	0.0939
Row 43	-0.1357	-0.2712 to -0.0002356	Yes	*	0.0492
Row 44	-0.1341	-0.2740 to 0.005738	No	ns	0.0717
Row 45	-0.1379	-0.2738 to -0.001899	Yes	*	0.0442
Row 46	-0.1404	-0.2695 to -0.01132	Yes	*	0.0231

Row 47	-0.1412	-0.2686 to -0.01383	Yes	*	0.0193
Row 48	-0.1422	-0.2647 to -0.01961	Yes	*	0.0123
Row 49	-0.1413	-0.2583 to -0.02438	Yes	**	0.0081
Row 50	-0.1478	-0.2613 to -0.03436	Yes	**	0.0037
Row 51	-0.1634	-0.2764 to -0.05036	Yes	**	0.0012
Row 52	-0.1458	-0.2585 to -0.03306	Yes	**	0.0039
Row 53	-0.1486	-0.2719 to -0.02537	Yes	**	0.0083
Row 54	-0.1596	-0.2751 to -0.04419	Yes	**	0.0019
Row 55	-0.1609	-0.2765 to -0.04529	Yes	**	0.0018
Row 56	-0.1467	-0.2556 to -0.03775	Yes	**	0.0034
Row 57	-0.1523	-0.2614 to -0.04317	Yes	**	0.0017
Row 58	-0.1482	-0.2551 to -0.04121	Yes	**	0.0021
Row 59	-0.1512	-0.2501 to -0.05234	Yes	***	0.0006
Row 60	-0.1432	-0.2557 to -0.03065	Yes	**	0.0049
Row 61	-0.1639	-0.2875 to -0.04035	Yes	**	0.0034
Row 62	-0.1644	-0.2881 to -0.04069	Yes	**	0.0031
Row 63	-0.1644	-0.2833 to -0.04561	Yes	**	0.002
Row 64	-0.1682	-0.2859 to -0.05056	Yes	**	0.0013
Row 65	-0.1722	-0.2912 to -0.05321	Yes	**	0.0012
Row 66	-0.1746	-0.2975 to -0.05178	Yes	**	0.0015
Row 67	-0.1787	-0.2895 to -0.06784	Yes	***	0.0004
Row 68	-0.1754	-0.2890 to -0.06180	Yes	***	0.0005
Row 69	-0.1754	-0.2842 to -0.06664	Yes	***	0.0004
Row 70	-0.1677	-0.2804 to -0.05508	Yes	***	0.0008
Row 71	-0.1662	-0.2842 to -0.04821	Yes	**	0.0016
Row 72	-0.1672	-0.2892 to -0.04514	Yes	**	0.0021
Row 73	-0.1661	-0.2850 to -0.04718	Yes	**	0.0017
Row 74	-0.1692	-0.2808 to -0.05764	Yes	***	0.0006
Row 75	-0.1582	-0.2730 to -0.04342	Yes	**	0.002
Row 76	-0.1609	-0.2760 to -0.04577	Yes	**	0.0017
Row 77	-0.153	-0.2721 to -0.03398	Yes	**	0.0042
Row 78	-0.1648	-0.2861 to -0.04356	Yes	**	0.0023
Row 79	-0.15	-0.2688 to -0.03120	Yes	**	0.0051
Row 80	-0.1535	-0.2652 to -0.04191	Yes	**	0.0021
Row 81	-0.1634	-0.2730 to -0.05382	Yes	**	0.001
Row 82	-0.1469	-0.2571 to -0.03668	Yes	**	0.0028
Row 83	-0.157	-0.2582 to -0.05586	Yes	***	0.0005
Row 84	-0.1556	-0.2537 to -0.05739	Yes	***	0.0004
Row 85	-0.141	-0.2404 to -0.04166	Yes	**	0.0014
Row 86	-0.1437	-0.2406 to -0.04677	Yes	***	0.0009
Row 87	-0.1467	-0.2503 to -0.04305	Yes	**	0.0014
Row 88	-0.1436	-0.2496 to -0.03768	Yes	**	0.0024
Row 89	-0.1558	-0.2640 to -0.04752	Yes	**	0.0012
Row 90	-0.1448	-0.2539 to -0.03578	Yes	**	0.003
Row 91	-0.1509	-0.2606 to -0.04127	Yes	**	0.002
Row 92	-0.1455	-0.2578 to -0.03309	Yes	**	0.0039
Row 93	-0.1416	-0.2548 to -0.02832	Yes	**	0.0057
Row 94	-0.1409	-0.2533 to -0.02843	Yes	**	0.0058
Row 95	-0.1412	-0.2568 to -0.02564	Yes	**	0.0073
Row 96	-0.1422	-0.2639 to -0.02059	Yes	*	0.0114

Row 97	-0.1463	-0.2660 to -0.02666	Yes	**	0.0073
Row 98	-0.1364	-0.2452 to -0.02769	Yes	**	0.0056
Row 99	-0.1471	-0.2423 to -0.05198	Yes	***	0.0005
Row 100	-0.1566	-0.2635 to -0.04963	Yes	***	0.001
Row 101	-0.1492	-0.2528 to -0.04563	Yes	**	0.0012

**FIGURE 2D**								
Test details	Mean 1	Mean 2	Mean Diff.	SE of diff.	N1	N2	t	DF
WT - α-syn OVX								
Row 1	0.008927	0.002926	0.006001	0.01096	9	9	0.5477	15.88
Row 2	0.003068	0.001913	0.001155	0.01371	9	9	0.08428	13.3
Row 3	0.003257	-0.004278	0.007535	0.008758	9	9	0.8603	14.16
Row 4	0.006738	0.003282	0.003456	0.007602	9	9	0.4547	14.73
Row 5	0.004503	-0.003785	0.008288	0.006797	9	9	1.22	11.75
Row 6	0.00003714	-0.006207	0.006244	0.00728	9	9	0.8577	15.27
Row 7	-0.0002558	0.005914	-0.00617	0.00617	9	9	0.9999	13.08
Row 8	-0.003436	-0.0004888	-0.002948	0.004952	9	9	0.5953	15.98
Row 9	0.002427	-0.001167	0.003594	0.003815	9	9	0.942	15.61
Row 10	0.001229	0.001948	-0.0007195	0.006677	9	9	0.1078	13.23
Row 11	0.03601	0.024	0.01201	0.008781	9	9	1.368	15.95
Row 12	0.1202	0.1119	0.008293	0.01529	9	9	0.5423	14.78
Row 13	0.2179	0.1969	0.02097	0.0231	9	9	0.9075	15.42
Row 14	0.3168	0.2903	0.02658	0.03146	9	9	0.8449	15.43
Row 15	0.4215	0.3805	0.04101	0.03677	9	9	1.115	15.66
Row 16	0.5171	0.4672	0.04986	0.04189	9	9	1.19	15.44
Row 17	0.6054	0.5503	0.05509	0.04305	9	9	1.279	15.71
Row 18	0.6909	0.6278	0.06311	0.04024	9	9	1.568	16
Row 19	0.7694	0.7001	0.06927	0.0374	9	9	1.852	15.81
Row 20	0.8368	0.7582	0.07865	0.03593	9	9	2.189	14.62
Row 21	0.8904	0.8211	0.06927	0.02711	9	9	2.556	15.14
Row 22	0.9284	0.8781	0.05032	0.0196	9	9	2.567	15
Row 23	0.9536	0.9306	0.02308	0.0151	9	9	1.529	16
Row 24	0.9789	0.9563	0.02254	0.01067	9	9	2.112	14.89
Row 25	0.9978	0.9954	0.002396	0.002838	9	9	0.8441	13.69
Row 26	0.977	0.9938	-0.0168	0.007747	9	9	2.168	11.3
Row 27	0.9061	0.9479	-0.04178	0.01102	9	9	3.791	14.52
Row 28	0.853	0.9017	-0.04871	0.01297	9	9	3.757	16
Row 29	0.8022	0.8613	-0.05911	0.01746	9	9	3.386	15.52
Row 30	0.7621	0.8295	-0.06744	0.0208	9	9	3.243	14.56
Row 31	0.728	0.8088	-0.08082	0.02322	9	9	3.48	14.57
Row 32	0.6972	0.7939	-0.09677	0.02634	9	9	3.674	15.83
Row 33	0.6702	0.7653	-0.0951	0.02458	9	9	3.87	15.3
Row 34	0.6517	0.7543	-0.1025	0.02866	9	9	3.578	15.85
Row 35	0.6346	0.7398	-0.1052	0.0282	9	9	3.73	15.98
Row 36	0.6151	0.7285	-0.1134	0.03279	9	9	3.458	15.98
Row 37	0.5897	0.7141	-0.1244	0.02976	9	9	4.179	15.53
Row 38	0.5726	0.6975	-0.1249	0.0308	9	9	4.054	15.47
Row 39	0.5584	0.6913	-0.1329	0.03556	9	9	3.737	14.54
Row 40	0.5438	0.6743	-0.1304	0.03683	9	9	3.541	14.65
Row 41	0.5365	0.6568	-0.1203	0.03409	9	9	3.528	15.8

Row 42	0.5072	0.644	-0.1367	0.03395	9	9	4.028	15.98
Row 43	0.4986	0.6343	-0.1357	0.03109	9	9	4.365	15.72
Row 44	0.4885	0.6227	-0.1341	0.03222	9	9	4.163	15.96
Row 45	0.4805	0.6184	-0.1379	0.03129	9	9	4.406	15.9
Row 46	0.4662	0.6066	-0.1404	0.02975	9	9	4.72	15.99
Row 47	0.4566	0.5978	-0.1412	0.02933	9	9	4.815	15.93
Row 48	0.4481	0.5903	-0.1422	0.02824	9	9	5.034	15.99
Row 49	0.4416	0.5829	-0.1413	0.02691	9	9	5.251	15.91
Row 50	0.4296	0.5774	-0.1478	0.02607	9	9	5.67	15.81
Row 51	0.4147	0.5781	-0.1634	0.02595	9	9	6.296	15.75
Row 52	0.4146	0.5604	-0.1458	0.02596	9	9	5.616	15.94
Row 53	0.4027	0.5514	-0.1486	0.02835	9	9	5.243	15.86
Row 54	0.3861	0.5457	-0.1596	0.02657	9	9	6.008	15.91
Row 55	0.3772	0.5381	-0.1609	0.02659	9	9	6.051	15.86
Row 56	0.3782	0.5249	-0.1467	0.02363	9	9	6.208	12.81
Row 57	0.3678	0.52	-0.1523	0.02507	9	9	6.074	15.8
Row 58	0.3619	0.5101	-0.1482	0.02415	9	9	6.135	14.75
Row 59	0.3519	0.5031	-0.1512	0.02279	9	9	6.637	15.98
Row 60	0.354	0.4971	-0.1432	0.02561	9	9	5.59	15.21
Row 61	0.3306	0.4945	-0.1639	0.02779	9	9	5.898	14.53
Row 62	0.3256	0.4899	-0.1644	0.0281	9	9	5.85	15.09
Row 63	0.3164	0.4809	-0.1644	0.02701	9	9	6.088	15.14
Row 64	0.3093	0.4775	-0.1682	0.02694	9	9	6.246	15.55
Row 65	0.298	0.4702	-0.1722	0.0272	9	9	6.332	15.46
Row 66	0.2914	0.466	-0.1746	0.02801	9	9	6.234	15.32
Row 67	0.2893	0.468	-0.1787	0.02507	9	9	7.127	14.85
Row 68	0.2813	0.4567	-0.1754	0.02617	9	9	6.702	15.97
Row 69	0.2809	0.4563	-0.1754	0.02469	9	9	7.103	15.06
Row 70	0.2725	0.4403	-0.1677	0.02595	9	9	6.463	15.97
Row 71	0.2713	0.4376	-0.1662	0.02707	9	9	6.139	15.71
Row 72	0.2665	0.4337	-0.1672	0.02809	9	9	5.951	15.92
Row 73	0.2624	0.4285	-0.1661	0.02734	9	9	6.075	15.84
Row 74	0.254	0.4232	-0.1692	0.0257	9	9	6.584	15.96
Row 75	0.2528	0.411	-0.1582	0.02642	9	9	5.987	15.92
Row 76	0.2533	0.4142	-0.1609	0.02637	9	9	6.1	15.61
Row 77	0.246	0.399	-0.153	0.02743	9	9	5.579	15.98
Row 78	0.239	0.4038	-0.1648	0.02795	9	9	5.898	16
Row 79	0.2418	0.3918	-0.15	0.02734	9	9	5.487	15.9
Row 80	0.2369	0.3904	-0.1535	0.0255	9	9	6.022	15.43
Row 81	0.2243	0.3877	-0.1634	0.02445	9	9	6.682	14.11
Row 82	0.2243	0.3712	-0.1469	0.02538	9	9	5.786	15.97
Row 83	0.2143	0.3714	-0.157	0.02322	9	9	6.764	15.72
Row 84	0.2161	0.3717	-0.1556	0.02244	9	9	6.932	15.47
Row 85	0.2187	0.3597	-0.141	0.0229	9	9	6.159	16
Row 86	0.2078	0.3515	-0.1437	0.02225	9	9	6.455	15.77
Row 87	0.2046	0.3512	-0.1467	0.02386	9	9	6.147	15.94
Row 88	0.2046	0.3482	-0.1436	0.02438	9	9	5.892	15.89
Row 89	0.1928	0.3485	-0.1558	0.02494	9	9	6.244	16
Row 90	0.1982	0.3431	-0.1448	0.02502	9	9	5.788	15.71
Row 91	0.1881	0.3391	-0.1509	0.02527	9	9	5.973	15.99

Row 92	0.1893	0.3348	-0.1455	0.0259	9	9	5.617	16
Row 93	0.1917	0.3332	-0.1416	0.0261	9	9	5.424	16
Row 94	0.184	0.3249	-0.1409	0.02565	9	9	5.493	15.34
Row 95	0.1807	0.3219	-0.1412	0.02657	9	9	5.316	15.82
Row 96	0.1742	0.3164	-0.1422	0.02802	9	9	5.076	15.97
Row 97	0.1707	0.317	-0.1463	0.02736	9	9	5.349	15.47
Row 98	0.176	0.3125	-0.1364	0.02493	9	9	5.472	15.67
Row 99	0.1644	0.3115	-0.1471	0.02181	9	9	6.745	15.66
Row 100	0.1578	0.3144	-0.1566	0.02464	9	9	6.354	15.99
Row 101	0.1649	0.3141	-0.1492	0.02384	9	9	6.261	15.88

**FIGURE 2E**	WT	+α-syn
Number of values	9	9
Mean	0.01742	0.01666
Std. Deviation	0.005345	0.004476
Std. Error of Mean	0.001782	0.001492
	WT	+α-syn
Test for normal distribution		
Shapiro-Wilk test		
W	0.9535	0.9108
P value	0.7287	0.3217
Passed normality test (alpha=0.05)?	Yes	Yes
P value summary	ns	ns
Column B	+α-syn	
vs.	vs.	
Column A	WT	
Unpaired t test		
P value	0.7504	
P value summary	ns	
Significantly different (P < 0.05)?	No	
One- or two-tailed P value?	Two-tailed	
t, df	t=0.3237, df=16	
How big is the difference?		
Mean of column A	0.01742	
Mean of column B	0.01666	
Difference between means (B - A) ± SEM	-0.0007522 ± 0.002324	
95% confidence interval	-0.005679 to 0.004174	
R squared (eta squared)	0.006505	
F test to compare variances		
F, DFn, Dfd	1.426, 8, 8	
P value	0.6276	
P value summary	ns	
Significantly different (P < 0.05)?	No	
Data analyzed		
Sample size, column A	9	
Sample size, column B	9	

**FIGURE 2F**	WT	+α-syn
Number of values	9	9
Mean	83.51	68.59
Std. Deviation	4.83	5.273
Std. Error of Mean	1.61	1.758
	WT	+α-syn
Test for normal distribution		
Shapiro-Wilk test		
W	0.9417	0.9341
P value	0.6001	0.5215
Passed normality test (alpha=0.05)?	Yes	Yes
P value summary	ns	ns
Column B	+α-syn	
vs.	vs.	
Column A	WT	
Unpaired t test		
P value	<0.0001	
P value summary	****	
Significantly different (P < 0.05)?	Yes	
One- or two-tailed P value?	Two-tailed	
t, df	t=6.261, df=16	
How big is the difference?		
Mean of column A	83.51	
Mean of column B	68.59	
Difference between means (B - A) ± SEM	-14.92 ± 2.384	
95% confidence interval	-19.98 to -9.871	
R squared (eta squared)	0.7102	
F test to compare variances		
F, DFn, Dfd	1.192, 8, 8	
P value	0.8102	
P value summary	ns	
Significantly different (P < 0.05)?	No	
Data analyzed		
Sample size, column A	9	
Sample size, column B	9	

**FIGURE 3B**					
Two-way RM ANOVA	Matching: Stacked				
Assume sphericity?	No				
Alpha	0.05				
Source of Variation	% of total variation	P value	P value summary	Significant?	Geisser-Greenhouse's epsilon
Time x Column Factor	0.04152	>0.9999	ns	No	
Time	91.98	<0.0001	****	Yes	0.02372
Column Factor	0.02974	0.5958	ns	No	
Subject	3.104	<0.0001	****	Yes	
ANOVA table	SS	DF	MS	F (DFn, DFd)	P value
Time x Column Factor	0.09841	100	0.0009841	F (100, 3000) = 0.2687	P>0.9999
Time	218	100	2.18	F (2.372, 71.15) = 595.2	P<0.0001
Column Factor	0.07049	1	0.07049	F (1, 30) = 0.2874	P=0.5958
Subject	7.358	30	0.2453	F (30, 3000) = 66.96	P<0.0001
Residual	10.99	3000	0.003663		

**FIGURE 3B**	
Difference between column means	
Mean of WT	0.3042
Mean of +α-syn	0.2949
Difference between means	0.009359
SE of difference	0.01746
95% CI of difference	-0.02629 to 0.04501
Data summary	
Number of columns (Column Factor)	2
Number of rows (Time)	101
Number of subjects (Subject)	32
Number of missing values	0
Compare each cell mean with the other cell mean in that row	
Number of families	1
Number of comparisons per family	101
Alpha	0.05

**FIGURE 3B**					
Šídák's multiple comparisons test	Mean Diff.	95.00% CI of diff.	Below threshold?	Summary	Adjusted P Value
WT - +α-syn					
Row 1	0.00562	-0.02276 to 0.03400	No	ns	>0.9999
Row 2	-0.003464	-0.02867 to 0.02174	No	ns	>0.9999

Row 3	0.007004	-0.01706 to 0.03107	No	ns	>0.9999
Row 4	0.005397	-0.01726 to 0.02805	No	ns	>0.9999
Row 5	0.006064	-0.02041 to 0.03254	No	ns	>0.9999
Row 6	0.0002384	-0.01507 to 0.01555	No	ns	>0.9999
Row 7	-0.005274	-0.02195 to 0.01140	No	ns	>0.9999
Row 8	0.006197	-0.007791 to 0.02019	No	ns	>0.9999
Row 9	0.003232	-0.01412 to 0.02058	No	ns	>0.9999
Row 10	-0.004392	-0.02292 to 0.01413	No	ns	>0.9999
Row 11	0.008239	-0.02936 to 0.04584	No	ns	>0.9999
Row 12	0.01717	-0.07843 to 0.1128	No	ns	>0.9999
Row 13	0.03136	-0.1099 to 0.1726	No	ns	>0.9999
Row 14	0.02673	-0.1416 to 0.1951	No	ns	>0.9999
Row 15	0.02545	-0.1682 to 0.2191	No	ns	>0.9999
Row 16	0.02144	-0.1655 to 0.2084	No	ns	>0.9999
Row 17	0.01577	-0.1453 to 0.1768	No	ns	>0.9999
Row 18	0.01468	-0.1196 to 0.1489	No	ns	>0.9999
Row 19	0.01858	-0.08023 to 0.1174	No	ns	>0.9999
Row 20	0.01535	-0.05648 to 0.08717	No	ns	>0.9999
Row 21	0.01516	-0.03651 to 0.06682	No	ns	>0.9999
Row 22	0.008612	-0.03653 to 0.05375	No	ns	>0.9999
Row 23	0.01633	-0.04045 to 0.07311	No	ns	>0.9999
Row 24	0.01444	-0.05349 to 0.08238	No	ns	>0.9999
Row 25	0.02548	-0.05478 to 0.1057	No	ns	>0.9999
Row 26	0.02841	-0.06504 to 0.1219	No	ns	>0.9999
Row 27	0.03361	-0.08877 to 0.1560	No	ns	>0.9999
Row 28	0.0313	-0.1141 to 0.1767	No	ns	>0.9999
Row 29	0.02839	-0.1341 to 0.1909	No	ns	>0.9999
Row 30	0.03785	-0.1384 to 0.2141	No	ns	>0.9999
Row 31	0.03247	-0.1429 to 0.2078	No	ns	>0.9999
Row 32	0.02854	-0.1436 to 0.2006	No	ns	>0.9999
Row 33	0.03357	-0.1475 to 0.2147	No	ns	>0.9999
Row 34	0.02284	-0.1514 to 0.1971	No	ns	>0.9999
Row 35	0.02868	-0.1328 to 0.1901	No	ns	>0.9999
Row 36	0.03176	-0.1269 to 0.1904	No	ns	>0.9999
Row 37	0.02566	-0.1228 to 0.1741	No	ns	>0.9999
Row 38	0.02401	-0.1247 to 0.1728	No	ns	>0.9999
Row 39	0.01798	-0.1272 to 0.1632	No	ns	>0.9999
Row 40	0.01299	-0.1265 to 0.1525	No	ns	>0.9999
Row 41	0.01543	-0.1214 to 0.1523	No	ns	>0.9999
Row 42	0.009539	-0.1231 to 0.1422	No	ns	>0.9999
Row 43	0.009732	-0.1202 to 0.1396	No	ns	>0.9999
Row 44	0.01296	-0.1140 to 0.1399	No	ns	>0.9999
Row 45	0.01423	-0.1054 to 0.1338	No	ns	>0.9999
Row 46	0.01272	-0.1023 to 0.1277	No	ns	>0.9999
Row 47	0.0002415	-0.1180 to 0.1185	No	ns	>0.9999
Row 48	0.01464	-0.09685 to 0.1261	No	ns	>0.9999
Row 49	0.008574	-0.09933 to 0.1165	No	ns	>0.9999
Row 50	0.009094	-0.09596 to 0.1141	No	ns	>0.9999
Row 51	-0.005226	-0.1122 to 0.1017	No	ns	>0.9999
Row 52	-0.00625	-0.1175 to 0.1050	No	ns	>0.9999

Row 53	0.00258	-0.1069 to 0.1121	No	ns	>0.9999
Row 54	-0.004332	-0.1108 to 0.1021	No	ns	>0.9999
Row 55	0.002356	-0.1008 to 0.1055	No	ns	>0.9999
Row 56	-0.004058	-0.1024 to 0.09426	No	ns	>0.9999
Row 57	-0.006715	-0.09798 to 0.08455	No	ns	>0.9999
Row 58	0.002034	-0.09610 to 0.1002	No	ns	>0.9999
Row 59	0.00568	-0.08925 to 0.1006	No	ns	>0.9999
Row 60	0.005833	-0.09433 to 0.1060	No	ns	>0.9999
Row 61	0.006011	-0.08702 to 0.09904	No	ns	>0.9999
Row 62	0.008429	-0.09049 to 0.1073	No	ns	>0.9999
Row 63	-0.001245	-0.1049 to 0.1024	No	ns	>0.9999
Row 64	0.004567	-0.09712 to 0.1063	No	ns	>0.9999
Row 65	0.009706	-0.08998 to 0.1094	No	ns	>0.9999
Row 66	0.01158	-0.08872 to 0.1119	No	ns	>0.9999
Row 67	0.001124	-0.09977 to 0.1020	No	ns	>0.9999
Row 68	0.002546	-0.08479 to 0.08988	No	ns	>0.9999
Row 69	0.002075	-0.09130 to 0.09545	No	ns	>0.9999
Row 70	0.006046	-0.09233 to 0.1044	No	ns	>0.9999
Row 71	-0.002531	-0.09618 to 0.09112	No	ns	>0.9999
Row 72	0.009882	-0.09575 to 0.1155	No	ns	>0.9999
Row 73	0.008835	-0.08276 to 0.1004	No	ns	>0.9999
Row 74	0.002282	-0.08869 to 0.09325	No	ns	>0.9999
Row 75	0.002851	-0.08677 to 0.09247	No	ns	>0.9999
Row 76	0.005491	-0.08928 to 0.1003	No	ns	>0.9999
Row 77	-0.002639	-0.09683 to 0.09156	No	ns	>0.9999
Row 78	0.01288	-0.07910 to 0.1049	No	ns	>0.9999
Row 79	0.006856	-0.08644 to 0.1001	No	ns	>0.9999
Row 80	0.003989	-0.09391 to 0.1019	No	ns	>0.9999
Row 81	-0.001291	-0.09375 to 0.09117	No	ns	>0.9999
Row 82	-0.007411	-0.09746 to 0.08264	No	ns	>0.9999
Row 83	-0.001302	-0.09768 to 0.09508	No	ns	>0.9999
Row 84	-0.0009392	-0.09190 to 0.09002	No	ns	>0.9999
Row 85	-0.003156	-0.09021 to 0.08390	No	ns	>0.9999
Row 86	0.002182	-0.09037 to 0.09474	No	ns	>0.9999
Row 87	0.001324	-0.08681 to 0.08946	No	ns	>0.9999
Row 88	-0.007835	-0.09591 to 0.08024	No	ns	>0.9999
Row 89	0.00573	-0.08326 to 0.09472	No	ns	>0.9999
Row 90	-0.006871	-0.09979 to 0.08605	No	ns	>0.9999
Row 91	0.004625	-0.08192 to 0.09117	No	ns	>0.9999
Row 92	-0.0009069	-0.08604 to 0.08422	No	ns	>0.9999
Row 93	0.01463	-0.07496 to 0.1042	No	ns	>0.9999
Row 94	0.001344	-0.08797 to 0.09065	No	ns	>0.9999
Row 95	0.006179	-0.08753 to 0.09989	No	ns	>0.9999
Row 96	0.002647	-0.08206 to 0.08736	No	ns	>0.9999
Row 97	0.003897	-0.08432 to 0.09212	No	ns	>0.9999
Row 98	0.005281	-0.08387 to 0.09444	No	ns	>0.9999
Row 99	0.002142	-0.08811 to 0.09239	No	ns	>0.9999
Row 100	0.008072	-0.08172 to 0.09786	No	ns	>0.9999
Row 101	0.005599	-0.08441 to 0.09560	No	ns	>0.9999

**FIGURE 2B**								
Test details	Mean 1	Mean 2	Mean Diff.	SE of diff.	N1	N2	t	DF
WT - +α-syn								
Row 1	-0.01969	-0.02531	0.00562	0.007283	15	17	0.7716	29.94
Row 2	-0.02307	-0.0196	-0.003464	0.006417	15	17	0.5397	28.1
Row 3	-0.01362	-0.02062	0.007004	0.00606	15	17	1.156	25.87
Row 4	-0.01007	-0.01547	0.005397	0.005718	15	17	0.9438	26.29
Row 5	-0.007691	-0.01376	0.006064	0.006785	15	17	0.8937	29.58
Row 6	-0.006282	-0.00652	0.0002384	0.003896	15	17	0.06118	27.97
Row 7	-0.008496	-0.003222	-0.005274	0.00411	15	17	1.283	22.42
Row 8	0.005702	-0.0004951	0.006197	0.00359	15	17	1.726	30
Row 9	0.003989	0.000757	0.003232	0.004352	15	17	0.7426	25.18
Row 10	0.005088	0.00948	-0.004392	0.004668	15	17	0.941	26
Row 11	0.07736	0.06912	0.008239	0.009515	15	17	0.8659	26.8
Row 12	0.2476	0.2304	0.01717	0.02439	15	17	0.704	28.57
Row 13	0.4172	0.3858	0.03136	0.03624	15	17	0.8655	29.94
Row 14	0.5608	0.534	0.02673	0.04319	15	17	0.6188	29.86
Row 15	0.6894	0.6639	0.02545	0.04971	15	17	0.5121	29.99
Row 16	0.7792	0.7578	0.02144	0.04797	15	17	0.447	29.98
Row 17	0.8496	0.8338	0.01577	0.04131	15	17	0.3817	29.86
Row 18	0.896	0.8813	0.01468	0.03429	15	17	0.4281	28.83
Row 19	0.9317	0.9132	0.01858	0.02524	15	17	0.736	28.82
Row 20	0.9519	0.9365	0.01535	0.01836	15	17	0.836	28.95
Row 21	0.9597	0.9446	0.01516	0.01322	15	17	1.146	29.33
Row 22	0.9616	0.953	0.008612	0.01146	15	17	0.7514	27.51
Row 23	0.9659	0.9496	0.01633	0.01451	15	17	1.125	29
Row 24	0.9571	0.9426	0.01444	0.0173	15	17	0.8348	28.19
Row 25	0.9599	0.9344	0.02548	0.02059	15	17	1.237	29.93
Row 26	0.9184	0.8899	0.02841	0.02392	15	17	1.188	29.3
Row 27	0.8127	0.7791	0.03361	0.03134	15	17	1.072	29.41
Row 28	0.7121	0.6808	0.0313	0.03727	15	17	0.84	29.57
Row 29	0.6311	0.6027	0.02839	0.04157	15	17	0.6829	29.25
Row 30	0.5678	0.53	0.03785	0.04478	15	17	0.8453	27.7
Row 31	0.5198	0.4873	0.03247	0.04462	15	17	0.7277	27.97
Row 32	0.4753	0.4467	0.02854	0.04359	15	17	0.6547	26.99
Row 33	0.4393	0.4057	0.03357	0.04603	15	17	0.7294	27.71
Row 34	0.4103	0.3874	0.02284	0.04422	15	17	0.5164	27.43
Row 35	0.3859	0.3572	0.02868	0.04094	15	17	0.7005	27.23
Row 36	0.3714	0.3396	0.03176	0.04	15	17	0.7941	26.08
Row 37	0.3507	0.325	0.02566	0.03744	15	17	0.6852	26.19
Row 38	0.3384	0.3144	0.02401	0.03761	15	17	0.6384	26.64
Row 39	0.3217	0.3037	0.01798	0.03602	15	17	0.4992	23.36
Row 40	0.3137	0.3008	0.01299	0.03494	15	17	0.3717	24.9
Row 41	0.3005	0.2851	0.01543	0.03419	15	17	0.4514	24.48
Row 42	0.2943	0.2848	0.009539	0.03358	15	17	0.2841	26.92
Row 43	0.2819	0.2722	0.009732	0.03252	15	17	0.2993	24.81
Row 44	0.2785	0.2655	0.01296	0.03217	15	17	0.4028	27.05
Row 45	0.2763	0.2621	0.01423	0.03007	15	17	0.4733	25.57
Row 46	0.2715	0.2588	0.01272	0.02914	15	17	0.4366	27.1
Row 47	0.26	0.2597	0.0002415	0.0301	15	17	0.008023	28.01

Row 48	0.2615	0.2469	0.01464	0.02829	15	17	0.5173	27.41
Row 49	0.2554	0.2468	0.008574	0.0275	15	17	0.3118	28.28
Row 50	0.2521	0.243	0.009094	0.02683	15	17	0.339	28.78
Row 51	0.2411	0.2463	-0.005226	0.02737	15	17	0.1909	29.34
Row 52	0.2371	0.2434	-0.00625	0.02824	15	17	0.2213	27.42
Row 53	0.2416	0.239	0.00258	0.02805	15	17	0.092	29.49
Row 54	0.233	0.2373	-0.004332	0.0273	15	17	0.1587	29.73
Row 55	0.229	0.2267	0.002356	0.0264	15	17	0.08925	29.35
Row 56	0.2267	0.2308	-0.004058	0.02523	15	17	0.1608	29.96
Row 57	0.2178	0.2246	-0.006715	0.0234	15	17	0.2869	29.75
Row 58	0.2247	0.2227	0.002034	0.02519	15	17	0.08075	30
Row 59	0.2208	0.2151	0.00568	0.02436	15	17	0.2332	29.93
Row 60	0.2216	0.2157	0.005833	0.02571	15	17	0.2269	29.99
Row 61	0.216	0.21	0.006011	0.02386	15	17	0.2519	29.79
Row 62	0.2165	0.208	0.008429	0.02538	15	17	0.332	29.95
Row 63	0.2081	0.2093	-0.001245	0.02657	15	17	0.04685	29.72
Row 64	0.2102	0.2056	0.004567	0.0261	15	17	0.175	29.98
Row 65	0.2105	0.2008	0.009706	0.02555	15	17	0.3799	29.65
Row 66	0.2077	0.1961	0.01158	0.02573	15	17	0.4498	29.9
Row 67	0.2003	0.1992	0.001124	0.02587	15	17	0.04344	29.71
Row 68	0.2013	0.1987	0.002546	0.02235	15	17	0.1139	29.3
Row 69	0.1953	0.1932	0.002075	0.02396	15	17	0.08661	29.94
Row 70	0.2001	0.194	0.006046	0.02522	15	17	0.2397	29.71
Row 71	0.1909	0.1934	-0.002531	0.024	15	17	0.1055	29.6
Row 72	0.1972	0.1873	0.009882	0.02708	15	17	0.3649	29.73
Row 73	0.189	0.1802	0.008835	0.02351	15	17	0.3758	29.99
Row 74	0.1866	0.1843	0.002282	0.02335	15	17	0.09774	29.98
Row 75	0.1802	0.1773	0.002851	0.023	15	17	0.124	29.97
Row 76	0.1846	0.1792	0.005491	0.02432	15	17	0.2257	29.98
Row 77	0.1783	0.1809	-0.002639	0.02418	15	17	0.1091	30
Row 78	0.1796	0.1667	0.01288	0.02359	15	17	0.5459	29.85
Row 79	0.1759	0.1691	0.006856	0.02392	15	17	0.2866	29.71
Row 80	0.1738	0.1698	0.003989	0.02507	15	17	0.1591	29.46
Row 81	0.1678	0.1691	-0.001291	0.02372	15	17	0.05444	29.89
Row 82	0.1631	0.1705	-0.007411	0.0231	15	17	0.3209	29.83
Row 83	0.1666	0.1679	-0.001302	0.02472	15	17	0.05266	29.78
Row 84	0.1601	0.161	-0.0009392	0.02334	15	17	0.04023	29.97
Row 85	0.1572	0.1604	-0.003156	0.02227	15	17	0.1417	29.13
Row 86	0.1604	0.1582	0.002182	0.02375	15	17	0.09188	29.98
Row 87	0.1561	0.1548	0.001324	0.0226	15	17	0.05859	29.8
Row 88	0.1476	0.1554	-0.007835	0.0226	15	17	0.3467	29.91
Row 89	0.1533	0.1476	0.00573	0.0227	15	17	0.2524	28.54
Row 90	0.1442	0.1511	-0.006871	0.02384	15	17	0.2881	29.94
Row 91	0.1422	0.1376	0.004625	0.02221	15	17	0.2082	30
Row 92	0.1408	0.1417	-0.0009069	0.02171	15	17	0.04177	28.47
Row 93	0.1455	0.1308	0.01463	0.02297	15	17	0.6369	29.74
Row 94	0.1429	0.1416	0.001344	0.02292	15	17	0.05865	30
Row 95	0.1454	0.1392	0.006179	0.02405	15	17	0.2569	29.99
Row 96	0.1384	0.1358	0.002647	0.02174	15	17	0.1218	30
Row 97	0.138	0.1341	0.003897	0.02255	15	17	0.1728	28.97

Row 98	0.1307	0.1254	0.005281	0.02288	15	17	0.2308	30
Row 99	0.1353	0.1332	0.002142	0.02314	15	17	0.09257	29.77
Row 100	0.1346	0.1265	0.008072	0.02305	15	17	0.3503	30
Row 101	0.1297	0.1241	0.005599	0.0231	15	17	0.2423	30

**FIGURE 3C**	WT	+α-syn
Number of values	15	17
Mean	0.2831	0.2556
Std. Deviation	0.0472	0.04652
Std. Error of Mean	0.01219	0.01128
	WT	+α-syn
Test for normal distribution		
Shapiro-Wilk test		
W	0.981	0.9426
P value	0.976	0.3502
Passed normality test (alpha=0.05)?	Yes	Yes
P value summary	ns	ns
Column B	+α-syn	
vs.	vs.	
Column A	WT	
Unpaired t test		
P value	0.1078	
P value summary	ns	
Significantly different (P < 0.05)?	No	
One- or two-tailed P value?	Two-tailed	
t, df	t=1.658, df=30	
How big is the difference?		
Mean of column A	0.2831	
Mean of column B	0.2556	
Difference between means (B - A) ± SEM	-0.02750 ± 0.01659	
95% confidence interval	-0.06139 to 0.006383	
R squared (eta squared)	0.0839	
F test to compare variances		
F, DFn, Dfd	1.029, 14, 16	
P value	0.9468	
P value summary	ns	
Significantly different (P < 0.05)?	No	
Data analyzed		
Sample size, column A	15	
Sample size, column B	17	

**FIGURE 3D**	WT	+α-syn
Number of values	15	17
Mean	0.02005	0.01782
Std. Deviation	0.003996	0.004723
Std. Error of Mean	0.001032	0.001145
	WT	+α-syn
Test for normal distribution		
Shapiro-Wilk test		
W	0.9681	0.9419
P value	0.8295	0.3418
Passed normality test (alpha=0.05)?	Yes	Yes
P value summary	ns	ns
Column B	+α-syn	
vs.	vs.	
Column A	WT	
Unpaired t test		
P value	0.1632	
P value summary	ns	
Significantly different (P < 0.05)?	No	
One- or two-tailed P value?	Two-tailed	
t, df	t=1.430, df=30	
How big is the difference?		
Mean of column A	0.02005	
Mean of column B	0.01782	
Difference between means (B - A) ± SEM	-0.002227 ± 0.001558	
95% confidence interval	-0.005410 to 0.0009548	
R squared (eta squared)	0.06377	
F test to compare variances		
F, DFn, Dfd	1.396, 16, 14	
P value	0.5358	
P value summary	ns	
Significantly different (P < 0.05)?	No	
Data analyzed		
Sample size, column A	15	
Sample size, column B	17	

**FIGURE 3E**	WT	+α-syn
Number of values	15	17
Mean	87.03	87.59
Std. Deviation	6.125	6.944
Std. Error of Mean	1.581	1.684
	WT	+α-syn
Test for normal distribution		
Shapiro-Wilk test		
W	0.8864	0.9404
P value	0.0592	0.3229
Passed normality test (alpha=0.05)?	Yes	Yes
P value summary	ns	ns
Column B	+α-syn	
vs.	vs.	
Column A	WT	
Unpaired t test		
P value	0.8117	
P value summary	ns	
Significantly different (P < 0.05)?	No	
One- or two-tailed P value?	Two-tailed	
t, df	t=0.2404, df=30	
How big is the difference?		
Mean of column A	87.03	
Mean of column B	87.59	
Difference between means (B - A) ± SEM	0.5599 ± 2.329	
95% confidence interval	-4.196 to 5.316	
R squared (eta squared)	0.001923	
F test to compare variances		
F, DFn, Dfd	1.285, 16, 14	
P value	0.6431	
P value summary	ns	
Significantly different (P < 0.05)?	No	
Data analyzed		
Sample size, column A	15	
Sample size, column B	17	

**FIGURE 3F**	WT	+α-syn
Number of values	15	17
Mean	19.43	17.94
Std. Deviation	10.03	6.488
Std. Error of Mean	2.589	1.574
	WT	+α-syn
Test for normal distribution		
Shapiro-Wilk test		
W	0.9352	0.955
P value	0.3254	0.5406
Passed normality test (alpha=0.05)?	Yes	Yes
P value summary	ns	ns
Column B	+α-syn	
vs.	vs.	
Column A	WT	
Unpaired t test		
P value	0.6143	
P value summary	ns	
Significantly different (P < 0.05)?	No	
One- or two-tailed P value?	Two-tailed	
t, df	t=0.5093, df=30	
How big is the difference?		
Mean of column A	19.43	
Mean of column B	17.93	
Difference between means (B - A) ± SEM	--1.503 ± 2.951	
95% confidence interval	-7.529 to 4.523	
R squared (eta squared)	0.008571	
F test to compare variances		
F, DFn, Dfd	2.389, 14, 16	
P value	0.0975	
P value summary	ns	
Significantly different (P < 0.05)?	No	
Data analyzed		
Sample size, column A	15	
Sample size, column B	17	

**FIGURE 4B**	mCer asyn	syp pc	mCer pc	syp asyn
Number of values	10	8	7	7
Mean	63.48	71.48	64.48	74.28
Std. Deviation	6.947	4.956	5.146	3.398
Std. Error of Mean	2.197	1.752	1.945	1.284
	WT mCer asyn	WT syp pc	WT mCer pc	WT syp asyn
Test for normal distribution				
Shapiro-Wilk test				
W	0.9666	0.8674	0.9556	0.9172
P value	0.8575	0.1421	0.78	0.4477
Passed normality test (alpha=0.05)?	Yes	Yes	Yes	Yes
P value summary	ns	ns	ns	ns
ANOVA summary				
F	7.428			
P value	0.0008			
P value summary	***			
Significant diff. among means (P < 0.05)?	Yes			
R squared	0.4432			
Brown-Forsythe test				
F (DFn, DFd)	0.8805 (3, 28)			
P value	0.4631			
P value summary	ns			
Are SDs significantly different (P < 0.05)?	No			
Bartlett's test				
Bartlett's statistic (corrected)	3.217			
P value	0.3594			
P value summary	ns			
Are SDs significantly different (P < 0.05)?	No			

**FIGURE 4B**					
ANOVA table	SS	DF	MS	F (DFn, DFd)	P value
Treatment (between columns)	664.1	3	221.4	F (3, 28) = 7.428	P=0.0008
Residual (within columns)	834.4	28	29.8		
Total	1498	31			
Data summary					
Number of treatments (columns)	4				
Number of values (total)	32				
Number of families	1				
Number of comparisons per family	3				
Alpha	0.05				

**FIGURE 4B**								
Dunnett's multiple comparisons test	Mean Diff.	95.00% CI of diff.	Below threshold?	Summary	Adjusted P Value	C-?		
mCer pc vs. mCer asyn	0.9987	-5.656 to 7.654	No	ns	0.9643	A	mCer asyn	
mCer pc vs. syp pc	-7.002	-13.99 to -0.01313	Yes	*	0.0495	B	syp pc	
mCer pc vs. syp asyn	-9.802	-17.02 to -2.584	Yes	**	0.0062	D	syp asyn	
Test details	Mean 1	Mean 2	Mean Diff.	SE of diff.	n1	n2	q	DF
mCer pc vs. mCer asyn	64.48	63.48	0.9987	2.69	7	10	0.3712	28
mCer pc vs.syp pc	64.48	71.48	-7.002	2.825	7	8	2.478	28
mCer pc vs. syp asyn	64.48	74.28	-9.802	2.918	7	7	3.359	28

**FIGURE 5B**					
Mixed-effects model (REML)	Matching: Stacked				
Assume sphericity?	No				
Alpha	0.05				
Fixed effects (type III)	P value	P value summary	Statistically significant (P < 0.05)?	F (DFn, DFd)	Geisser-Greenhouse's epsilon
Time	<0.0001	****	Yes	F (2.752, 74.25) = 540.2	0.02752
Column Factor	0.0009	***	Yes	F (2, 27) = 9.181	
Time x Column Factor	<0.0001	****	Yes	F (200, 2698) = 5.606	
Random effects	SD	Variance			
Subject	0.05605	0.003142			
Residual	0.05829	0.003398			

**FIGURE 5B**	
Was the matching effective?	
Chi-square, df	1661, 1
P value	<0.0001
P value summary	****
Is there significant matching (P < 0.05)?	Yes
Data summary	
Number of columns (Column Factor)	3
Number of rows (Time)	101
Number of subjects (Subject)	30
Number of missing values	2
Within each row, compare columns (simple effects within rows)	
Number of families	101
Number of comparisons per family	2
Alpha	0.05

**FIGURE 5B**					
Šídák's multiple comparisons test	Mean Diff.	95.00% CI of diff.	Below threshold?	Summary	Adjusted P Value
Row 1					
WT vs. +α-syn^{WT}	0.00002767	-0.02408 to 0.02414	No	ns	>0.9999
WT vs. +α-syn^{AAA}	-0.001464	-0.02944 to 0.02651	No	ns	0.9896
Row 2					
WT vs. +α-syn^{WT}	-0.005245	-0.03088 to 0.02039	No	ns	0.8579
WT vs. +α-syn^{AAA}	-0.008437	-0.03427 to 0.01740	No	ns	0.6752

Row 3					
WT vs. +α-syn^{WT}	-0.00577	-0.03113 to 0.01959	No	ns	0.8249
WT vs. +α-syn^{AAA}	-0.002471	-0.03040 to 0.02546	No	ns	0.9711
Row 4					
WT vs. +α-syn^{WT}	-0.003188	-0.02094 to 0.01457	No	ns	0.8882
WT vs. +α-syn^{AAA}	-0.001296	-0.02283 to 0.02024	No	ns	0.9864
Row 5					
WT vs. +α-syn^{WT}	-0.0002318	-0.01245 to 0.01199	No	ns	0.9987
WT vs. +α-syn^{AAA}	-0.0004001	-0.01661 to 0.01581	No	ns	0.9976
Row 6					
WT vs. +α-syn^{WT}	0.00492	-0.004228 to 0.01407	No	ns	0.3661
WT vs. +α-syn^{AAA}	0.00549	-0.008446 to 0.01943	No	ns	0.565
Row 7					
WT vs. +α-syn^{WT}	0.001833	-0.007589 to 0.01125	No	ns	0.8696
WT vs. +α-syn^{AAA}	0.002658	-0.008069 to 0.01339	No	ns	0.7971
Row 8					
WT vs. +α-syn^{WT}	0.002057	-0.009804 to 0.01392	No	ns	0.8905
WT vs. +α-syn^{AAA}	0.002971	-0.008364 to 0.01431	No	ns	0.7594
Row 9					
WT vs. +α-syn^{WT}	-0.005515	-0.01786 to 0.006830	No	ns	0.4853
WT vs. +α-syn^{AAA}	-0.001647	-0.01514 to 0.01185	No	ns	0.9455
Row 10					
WT vs. +α-syn^{WT}	-0.003299	-0.01418 to 0.007585	No	ns	0.7114
WT vs. +α-syn^{AAA}	-0.009475	-0.02425 to 0.005300	No	ns	0.2346
Row 11					
WT vs. +α-syn^{WT}	0.00348	-0.01098 to 0.01794	No	ns	0.8097
WT vs. +α-syn^{AAA}	-0.00606	-0.02708 to 0.01496	No	ns	0.7312
Row 12					
WT vs. +α-syn^{WT}	0.02383	-0.01997 to 0.06764	No	ns	0.358
WT vs. +α-syn^{AAA}	0.002499	-0.03988 to 0.04487	No	ns	0.9868
Row 13					
WT vs. +α-syn^{WT}	0.06329	-0.02007 to 0.1466	No	ns	0.1546
WT vs. +α-syn^{AAA}	0.01198	-0.05696 to 0.08093	No	ns	0.8926
Row 14					
WT vs. +α-syn^{WT}	0.05966	-0.03143 to 0.1507	No	ns	0.2365
WT vs. +α-syn^{AAA}	0.01019	-0.07583 to 0.09620	No	ns	0.9477

Row 15					
WT vs. +α-syn^{WT}	0.06791	-0.03020 to 0.1660	No	ns	0.2038
WT vs. +α-syn^{AAA}	0.0146	-0.07838 to 0.1076	No	ns	0.9106
Row 16					
WT vs. +α-syn^{WT}	0.06385	-0.03187 to 0.1596	No	ns	0.2259
WT vs. +α-syn^{AAA}	0.02142	-0.07020 to 0.1130	No	ns	0.8148
Row 17					
WT vs. +α-syn^{WT}	0.07284	-0.01795 to 0.1636	No	ns	0.1273
WT vs. +α-syn^{AAA}	0.03195	-0.04893 to 0.1128	No	ns	0.5618
Row 18					
WT vs. +α-syn^{WT}	0.06993	-0.01480 to 0.1547	No	ns	0.1151
WT vs. +α-syn^{AAA}	0.02818	-0.04502 to 0.1014	No	ns	0.5816
Row 19					
WT vs. +α-syn^{WT}	0.07648	0.003976 to 0.1490	Yes	*	0.038
WT vs. +α-syn^{AAA}	0.03979	-0.02299 to 0.1026	No	ns	0.2543
Row 20					
WT vs. +α-syn^{WT}	0.05275	-0.008163 to 0.1137	No	ns	0.0954
WT vs. +α-syn^{AAA}	0.02947	-0.02098 to 0.07991	No	ns	0.3089
Row 21					
WT vs. +α-syn^{WT}	0.05321	0.001306 to 0.1051	Yes	*	0.0441
WT vs. +α-syn^{AAA}	0.0321	-0.01550 to 0.07970	No	ns	0.218
Row 22					
WT vs. +α-syn^{WT}	0.04734	0.008410 to 0.08626	Yes	*	0.0165
WT vs. +α-syn^{AAA}	0.03465	-0.004160 to 0.07347	No	ns	0.0835
Row 23					
WT vs. +α-syn^{WT}	0.01986	-0.006722 to 0.04644	No	ns	0.1621
WT vs. +α-syn^{AAA}	0.01522	-0.01092 to 0.04136	No	ns	0.3087
Row 24					
WT vs. +α-syn^{WT}	0.01315	-0.006317 to 0.03262	No	ns	0.2197
WT vs. +α-syn^{AAA}	0.01587	-0.005206 to 0.03695	No	ns	0.1555
Row 25					
WT vs. +α-syn^{WT}	-0.003932	-0.02247 to 0.01461	No	ns	0.829
WT vs. +α-syn^{AAA}	-0.004479	-0.02352 to 0.01456	No	ns	0.8027
Row 26					
WT vs. +α-syn^{WT}	-0.01771	-0.04228 to 0.006864	No	ns	0.1591
WT vs. +α-syn^{AAA}	-0.009312	-0.03529 to 0.01667	No	ns	0.6132
Row 27					

WT vs. +α-syn^{WT}	-0.03471	-0.07113 to 0.001718	No	ns	0.0621
WT vs. +α-syn^{AAA}	-0.02299	-0.05899 to 0.01301	No	ns	0.237
Row 28					
WT vs. +α-syn^{WT}	-0.05776	-0.1023 to -0.01323	Yes	*	0.0114
WT vs. +α-syn^{AAA}	-0.02789	-0.06929 to 0.01350	No	ns	0.2078
Row 29					
WT vs. +α-syn^{WT}	-0.06121	-0.1119 to -0.01056	Yes	*	0.0173
WT vs. +α-syn^{AAA}	-0.02091	-0.06894 to 0.02713	No	ns	0.5057
Row 30					
WT vs. +α-syn^{WT}	-0.06361	-0.1115 to -0.01570	Yes	**	0.0092
WT vs. +α-syn^{AAA}	-0.01809	-0.06491 to 0.02874	No	ns	0.5832
Row 31					
WT vs. +α-syn^{WT}	-0.06952	-0.1270 to -0.01203	Yes	*	0.0173
WT vs. +α-syn^{AAA}	-0.02643	-0.08151 to 0.02865	No	ns	0.44
Row 32					
WT vs. +α-syn^{WT}	-0.06565	-0.1229 to -0.008399	Yes	*	0.0242
WT vs. +α-syn^{AAA}	-0.02214	-0.08494 to 0.04067	No	ns	0.6366
Row 33					
WT vs. +α-syn^{WT}	-0.07523	-0.1416 to -0.008916	Yes	*	0.0256
WT vs. +α-syn^{AAA}	-0.02848	-0.1013 to 0.04433	No	ns	0.5759
Row 34					
WT vs. +α-syn^{WT}	-0.0856	-0.1537 to -0.01745	Yes	*	0.0138
WT vs. +α-syn^{AAA}	-0.02658	-0.1009 to 0.04775	No	ns	0.6285
Row 35					
WT vs. +α-syn^{WT}	-0.09438	-0.1672 to -0.02160	Yes	*	0.0116
WT vs. +α-syn^{AAA}	-0.03434	-0.1190 to 0.05029	No	ns	0.5534
Row 36					
WT vs. +α-syn^{WT}	-0.09425	-0.1746 to -0.01391	Yes	*	0.0218
WT vs. +α-syn^{AAA}	-0.01903	-0.1139 to 0.07587	No	ns	0.8612
Row 37					
WT vs. +α-syn^{WT}	-0.1	-0.1787 to -0.02135	Yes	*	0.013
WT vs. +α-syn^{AAA}	-0.02547	-0.1169 to 0.06591	No	ns	0.7516
Row 38					
WT vs. +α-syn^{WT}	-0.1116	-0.1865 to -0.03662	Yes	**	0.004
WT vs. +α-syn^{AAA}	-0.01631	-0.1125 to 0.07988	No	ns	0.8976
Row 39					
WT vs. +α-syn^{WT}	-0.1275	-0.1962 to -0.05873	Yes	***	0.0006

WT vs. +α-syn^{AAA}	-0.04629	-0.1363 to 0.04376	No	ns	0.3906
Row 40					
WT vs. +α-syn^{WT}	-0.1187	-0.2155 to -0.02201	Yes	*	0.0169
WT vs. +α-syn^{AAA}	-0.0401	-0.1505 to 0.07033	No	ns	0.6193
Row 41					
WT vs. +α-syn^{WT}	-0.1263	-0.1993 to -0.05340	Yes	**	0.001
WT vs. +α-syn^{AAA}	-0.04823	-0.1466 to 0.05009	No	ns	0.422
Row 42					
WT vs. +α-syn^{WT}	-0.1495	-0.2282 to -0.07080	Yes	***	0.0005
WT vs. +α-syn^{AAA}	-0.06953	-0.1693 to 0.03021	No	ns	0.1975
Row 43					
WT vs. +α-syn^{WT}	-0.1551	-0.2485 to -0.06178	Yes	**	0.0018
WT vs. +α-syn^{AAA}	-0.07883	-0.1925 to 0.03483	No	ns	0.2014
Row 44					
WT vs. +α-syn^{WT}	-0.1616	-0.2631 to -0.06004	Yes	**	0.0025
WT vs. +α-syn^{AAA}	-0.08085	-0.1953 to 0.03358	No	ns	0.191
Row 45					
WT vs. +α-syn^{WT}	-0.1416	-0.2316 to -0.05161	Yes	**	0.0024
WT vs. +α-syn^{AAA}	-0.06366	-0.1717 to 0.04436	No	ns	0.3014
Row 46					
WT vs. +α-syn^{WT}	-0.1464	-0.2351 to -0.05765	Yes	**	0.0016
WT vs. +α-syn^{AAA}	-0.05707	-0.1630 to 0.04885	No	ns	0.3594
Row 47					
WT vs. +α-syn^{WT}	-0.1495	-0.2412 to -0.05779	Yes	**	0.0018
WT vs. +α-syn^{AAA}	-0.07034	-0.1838 to 0.04311	No	ns	0.2683
Row 48					
WT vs. +α-syn^{WT}	-0.1612	-0.2600 to -0.06249	Yes	**	0.0018
WT vs. +α-syn^{AAA}	-0.06575	-0.1812 to 0.04972	No	ns	0.3256
Row 49					
WT vs. +α-syn^{WT}	-0.1678	-0.2691 to -0.06652	Yes	**	0.0016
WT vs. +α-syn^{AAA}	-0.06491	-0.1775 to 0.04772	No	ns	0.3183
Row 50					
WT vs. +α-syn^{WT}	-0.161	-0.2675 to -0.05459	Yes	**	0.0037
WT vs. +α-syn^{AAA}	-0.06442	-0.1803 to 0.05146	No	ns	0.3423
Row 51					
WT vs. +α-syn^{WT}	-0.1591	-0.2641 to -0.05421	Yes	**	0.0036
WT vs. +α-syn^{AAA}	-0.0606	-0.1757 to 0.05450	No	ns	0.3795

Row 52					
WT vs. +α-syn^{WT}	-0.1683	-0.2750 to -0.06165	Yes	**	0.0025
WT vs. +α-syn^{AAA}	-0.07092	-0.1807 to 0.03887	No	ns	0.2438
Row 53					
WT vs. +α-syn^{WT}	-0.1706	-0.2718 to -0.06949	Yes	**	0.0014
WT vs. +α-syn^{AAA}	-0.06863	-0.1775 to 0.04026	No	ns	0.2597
Row 54					
WT vs. +α-syn^{WT}	-0.1683	-0.2697 to -0.06699	Yes	**	0.0016
WT vs. +α-syn^{AAA}	-0.06562	-0.1737 to 0.04246	No	ns	0.2836
Row 55					
WT vs. +α-syn^{WT}	-0.1651	-0.2671 to -0.06316	Yes	**	0.0021
WT vs. +α-syn^{AAA}	-0.06119	-0.1726 to 0.05019	No	ns	0.3504
Row 56					
WT vs. +α-syn^{WT}	-0.1682	-0.2668 to -0.06965	Yes	**	0.0013
WT vs. +α-syn^{AAA}	-0.05916	-0.1672 to 0.04885	No	ns	0.3526
Row 57					
WT vs. +α-syn^{WT}	-0.171	-0.2784 to -0.06355	Yes	**	0.0025
WT vs. +α-syn^{AAA}	-0.06214	-0.1734 to 0.04911	No	ns	0.3373
Row 58					
WT vs. +α-syn^{WT}	-0.1776	-0.2792 to -0.07611	Yes	**	0.001
WT vs. +α-syn^{AAA}	-0.06639	-0.1699 to 0.03711	No	ns	0.248
Row 59					
WT vs. +α-syn^{WT}	-0.1708	-0.2657 to -0.07590	Yes	***	0.0007
WT vs. +α-syn^{AAA}	-0.06191	-0.1608 to 0.03700	No	ns	0.2641
Row 60					
WT vs. +α-syn^{WT}	-0.1803	-0.2847 to -0.07585	Yes	**	0.0012
WT vs. +α-syn^{AAA}	-0.07392	-0.1790 to 0.03117	No	ns	0.1924
Row 61					
WT vs. +α-syn^{WT}	-0.1742	-0.2656 to -0.08291	Yes	***	0.0005
WT vs. +α-syn^{AAA}	-0.07277	-0.1687 to 0.02312	No	ns	0.1527
Row 62					
WT vs. +α-syn^{WT}	-0.1772	-0.2744 to -0.08011	Yes	***	0.0006
WT vs. +α-syn^{AAA}	-0.07116	-0.1674 to 0.02511	No	ns	0.1661
Row 63					
WT vs. +α-syn^{WT}	-0.182	-0.2840 to -0.08002	Yes	***	0.0009
WT vs. +α-syn^{AAA}	-0.07666	-0.1755 to 0.02215	No	ns	0.1406

Row 64					
WT vs. +α-syn^{WT}	-0.1837	-0.2929 to -0.07457	Yes	**	0.0016
WT vs. +α-syn^{AAA}	-0.0855	-0.1906 to 0.01956	No	ns	0.1178
Row 65					
WT vs. +α-syn^{WT}	-0.1642	-0.2850 to -0.04337	Yes	**	0.009
WT vs. +α-syn^{AAA}	-0.05478	-0.1705 to 0.06097	No	ns	0.4287
Row 66					
WT vs. +α-syn^{WT}	-0.1812	-0.2938 to -0.06866	Yes	**	0.0023
WT vs. +α-syn^{AAA}	-0.0889	-0.1966 to 0.01885	No	ns	0.1113
Row 67					
WT vs. +α-syn^{WT}	-0.1719	-0.2797 to -0.06423	Yes	**	0.0023
WT vs. +α-syn^{AAA}	-0.0767	-0.1777 to 0.02426	No	ns	0.1496
Row 68					
WT vs. +α-syn^{WT}	-0.1687	-0.2744 to -0.06294	Yes	**	0.0023
WT vs. +α-syn^{AAA}	-0.07273	-0.1733 to 0.02782	No	ns	0.1752
Row 69					
WT vs. +α-syn^{WT}	-0.1641	-0.2687 to -0.05951	Yes	**	0.0026
WT vs. +α-syn^{AAA}	-0.06685	-0.1650 to 0.03134	No	ns	0.2093
Row 70					
WT vs. +α-syn^{WT}	-0.1625	-0.2638 to -0.06122	Yes	**	0.0021
WT vs. +α-syn^{AAA}	-0.07101	-0.1663 to 0.02433	No	ns	0.1606
Row 71					
WT vs. +α-syn^{WT}	-0.1744	-0.2786 to -0.07012	Yes	**	0.0015
WT vs. +α-syn^{AAA}	-0.07499	-0.1705 to 0.02051	No	ns	0.134
Row 72					
WT vs. +α-syn^{WT}	-0.1764	-0.2809 to -0.07177	Yes	**	0.0014
WT vs. +α-syn^{AAA}	-0.08155	-0.1793 to 0.01623	No	ns	0.1084
Row 73					
WT vs. +α-syn^{WT}	-0.1787	-0.2818 to -0.07567	Yes	**	0.0011
WT vs. +α-syn^{AAA}	-0.08222	-0.1782 to 0.01377	No	ns	0.098
Row 74					
WT vs. +α-syn^{WT}	-0.1816	-0.2891 to -0.07406	Yes	**	0.0014
WT vs. +α-syn^{AAA}	-0.09912	-0.1997 to 0.001471	No	ns	0.0536
Row 75					
WT vs. +α-syn^{WT}	-0.1732	-0.2736 to -0.07274	Yes	**	0.0011
WT vs. +α-syn^{AAA}	-0.08968	-0.1808 to 0.001434	No	ns	0.0539
Row 76					

WT vs. +α-syn^{WT}	-0.1709	-0.2719 to -0.06999	Yes	**	0.0013
WT vs. +α-syn^{AAA}	-0.08518	-0.1781 to 0.007782	No	ns	0.0745
Row 77					
WT vs. +α-syn^{WT}	-0.1772	-0.2812 to -0.07321	Yes	**	0.0012
WT vs. +α-syn^{AAA}	-0.0901	-0.1845 to 0.004311	No	ns	0.0622
Row 78					
WT vs. +α-syn^{WT}	-0.1867	-0.2906 to -0.08288	Yes	***	0.0007
WT vs. +α-syn^{AAA}	-0.09718	-0.1892 to -0.005179	Yes	*	0.0381
Row 79					
WT vs. +α-syn^{WT}	-0.1806	-0.2810 to -0.08015	Yes	***	0.0007
WT vs. +α-syn^{AAA}	-0.09357	-0.1857 to -0.001397	Yes	*	0.0465
Row 80					
WT vs. +α-syn^{WT}	-0.1799	-0.2859 to -0.07398	Yes	**	0.0012
WT vs. +α-syn^{AAA}	-0.1057	-0.1996 to -0.01183	Yes	*	0.0271
Row 81					
WT vs. +α-syn^{WT}	-0.1761	-0.2814 to -0.07076	Yes	**	0.0014
WT vs. +α-syn^{AAA}	-0.0901	-0.1843 to 0.004098	No	ns	0.0616
Row 82					
WT vs. +α-syn^{WT}	-0.171	-0.2830 to -0.05894	Yes	**	0.0033
WT vs. +α-syn^{AAA}	-0.0874	-0.1892 to 0.01439	No	ns	0.0963
Row 83					
WT vs. +α-syn^{WT}	-0.178	-0.2892 to -0.06681	Yes	**	0.0022
WT vs. +α-syn^{AAA}	-0.09808	-0.1967 to 0.0005445	No	ns	0.0513
Row 84					
WT vs. +α-syn^{WT}	-0.1676	-0.2843 to -0.05088	Yes	**	0.0052
WT vs. +α-syn^{AAA}	-0.08541	-0.1905 to 0.01965	No	ns	0.1182
Row 85					
WT vs. +α-syn^{WT}	-0.1758	-0.2934 to -0.05817	Yes	**	0.0039
WT vs. +α-syn^{AAA}	-0.09053	-0.1987 to 0.01768	No	ns	0.1062
Row 86					
WT vs. +α-syn^{WT}	-0.1744	-0.2937 to -0.05502	Yes	**	0.0047
WT vs. +α-syn^{AAA}	-0.06907	-0.1772 to 0.03910	No	ns	0.2437
Row 87					
WT vs. +α-syn^{WT}	-0.1668	-0.2829 to -0.05067	Yes	**	0.0053
WT vs. +α-syn^{AAA}	-0.0781	-0.1832 to 0.02698	No	ns	0.1595
Row 88					
WT vs. +α-syn^{WT}	-0.1719	-0.2882 to -0.05559	Yes	**	0.0042

WT vs. +α-syn^{AAA}	-0.09046	-0.1949 to 0.01392	No	ns	0.0926
Row 89					
WT vs. +α-syn^{WT}	-0.1798	-0.2996 to -0.05996	Yes	**	0.0037
WT vs. +α-syn^{AAA}	-0.08764	-0.1926 to 0.01735	No	ns	0.1067
Row 90					
WT vs. +α-syn^{WT}	-0.1747	-0.2979 to -0.05148	Yes	**	0.0057
WT vs. +α-syn^{AAA}	-0.08535	-0.1925 to 0.02177	No	ns	0.1263
Row 91					
WT vs. +α-syn^{WT}	-0.1682	-0.2895 to -0.04689	Yes	**	0.0069
WT vs. +α-syn^{AAA}	-0.07972	-0.1896 to 0.03016	No	ns	0.1705
Row 92					
WT vs. +α-syn^{WT}	-0.1807	-0.2977 to -0.06363	Yes	**	0.003
WT vs. +α-syn^{AAA}	-0.0892	-0.1934 to 0.01495	No	ns	0.0965
Row 93					
WT vs. +α-syn^{WT}	-0.181	-0.3018 to -0.06014	Yes	**	0.0038
WT vs. +α-syn^{AAA}	-0.08241	-0.1890 to 0.02415	No	ns	0.1387
Row 94					
WT vs. +α-syn^{WT}	-0.1739	-0.2954 to -0.05242	Yes	**	0.0055
WT vs. +α-syn^{AAA}	-0.0867	-0.1940 to 0.02064	No	ns	0.1182
Row 95					
WT vs. +α-syn^{WT}	-0.1806	-0.3038 to -0.05736	Yes	**	0.0045
WT vs. +α-syn^{AAA}	-0.08039	-0.1856 to 0.02486	No	ns	0.1418
Row 96					
WT vs. +α-syn^{WT}	-0.1845	-0.3028 to -0.06620	Yes	**	0.0027
WT vs. +α-syn^{AAA}	-0.07831	-0.1801 to 0.02346	No	ns	0.1407
Row 97					
WT vs. +α-syn^{WT}	-0.1723	-0.2911 to -0.05344	Yes	**	0.0049
WT vs. +α-syn^{AAA}	-0.06324	-0.1659 to 0.03946	No	ns	0.2615
Row 98					
WT vs. +α-syn^{WT}	-0.1785	-0.3001 to -0.05704	Yes	**	0.0043
WT vs. +α-syn^{AAA}	-0.07789	-0.1781 to 0.02231	No	ns	0.1341
Row 99					
WT vs. +α-syn^{WT}	-0.189	-0.3096 to -0.06836	Yes	**	0.0026
WT vs. +α-syn^{AAA}	-0.08247	-0.1853 to 0.02034	No	ns	0.1216
Row 100					
WT vs. +α-syn^{WT}	-0.1813	-0.3022 to -0.06043	Yes	**	0.0036
WT vs. +α-syn^{AAA}	-0.07939	-0.1781 to 0.01929	No	ns	0.1205

Row 101					
WT vs. +α-syn^{WT}	-0.1829	-0.3019 to -0.06392	Yes	**	0.003
WT vs. +α-syn^{AAA}	-0.07727	-0.1790 to 0.02443	No	ns	0.1461

**FIGURE 5B**								
Test details	Mean 1	Mean 2	Mean Diff.	SE of diff.	N1	N2	t	DF
Row 1								
WT vs. +α-syn^{WT}	0.0132	0.01317	0.00002767	0.009884	8	12	0.002799	17.98
WT vs. +α-syn^{AAA}	0.0132	0.01466	-0.001464	0.01124	8	10	0.1303	14.65
Row 2								
WT vs. +α-syn^{WT}	0.007853	0.0131	-0.005245	0.01048	8	12	0.5003	17.49
WT vs. +α-syn^{AAA}	0.007853	0.01629	-0.008437	0.0104	8	10	0.811	15.02
Row 3								
WT vs. +α-syn^{WT}	0.008366	0.01414	-0.00577	0.01025	8	12	0.5628	15.56
WT vs. +α-syn^{AAA}	0.008366	0.01084	-0.002471	0.01132	8	10	0.2184	15.9
Row 4								
WT vs. +α-syn^{WT}	0.004447	0.007635	-0.003188	0.007253	8	12	0.4396	17.3
WT vs. +α-syn^{AAA}	0.004447	0.005743	-0.001296	0.008696	8	10	0.149	15.39
Row 5								
WT vs. +α-syn^{WT}	0.00339	0.003622	-0.0002318	0.005001	8	12	0.04634	17.59
WT vs. +α-syn^{AAA}	0.00339	0.00379	-0.0004001	0.006408	8	10	0.06243	12.84
Row 6								
WT vs. +α-syn^{WT}	0.004552	-0.0003683	0.00492	0.003717	8	12	1.324	16.34
WT vs. +α-syn^{AAA}	0.004552	-0.0009384	0.00549	0.005562	8	10	0.9871	13.87
Row 7								
WT vs. +α-syn^{WT}	0.003651	0.001819	0.001833	0.003835	8	12	0.4779	16.63
WT vs. +α-syn^{AAA}	0.003651	0.0009934	0.002658	0.004348	8	10	0.6113	15.95
Row 8								
WT vs. +α-syn^{WT}	0.0008871	-0.00117	0.002057	0.004707	8	12	0.437	13.24
WT vs. +α-syn^{AAA}	0.0008871	-0.002084	0.002971	0.00435	8	10	0.6829	10.49
Row 9								
WT vs. +α-syn^{WT}	-0.003446	0.002069	-0.005515	0.004937	8	12	1.117	14.11
WT vs. +α-syn^{AAA}	-0.003446	-0.001799	-0.001647	0.005453	8	10	0.3021	15.48
Row 10								
WT vs. +α-syn^{WT}	-0.005646	-0.002347	-0.003299	0.004377	8	12	0.7537	14.81
WT vs. +α-syn^{AAA}	-0.005646	0.003829	-0.009475	0.005702	8	10	1.662	10.85
Row 11								

WT vs. +α-syn^{WT}	0.03115	0.02767	0.00348	0.005914	8	12	0.5885	17.48
WT vs. +α-syn^{AAA}	0.03115	0.03721	-0.00606	0.008377	8	10	0.7235	13.73
Row 12								
WT vs. +α-syn^{WT}	0.1459	0.1221	0.02383	0.01779	8	12	1.34	16.28
WT vs. +α-syn^{AAA}	0.1459	0.1434	0.002499	0.01699	8	10	0.147	14.45
Row 13								
WT vs. +α-syn^{WT}	0.2767	0.2134	0.06329	0.03417	8	12	1.852	17.97
WT vs. +α-syn^{AAA}	0.2767	0.2647	0.01198	0.02778	8	10	0.4313	15.1
Row 14								
WT vs. +α-syn^{WT}	0.3953	0.3357	0.05966	0.03704	8	12	1.611	16.47
WT vs. +α-syn^{AAA}	0.3953	0.3852	0.01019	0.03437	8	10	0.2963	14.02
Row 15								
WT vs. +α-syn^{WT}	0.5134	0.4455	0.06791	0.03995	8	12	1.7	16.73
WT vs. +α-syn^{AAA}	0.5134	0.4988	0.0146	0.03728	8	10	0.3917	14.44
Row 16								
WT vs. +α-syn^{WT}	0.6125	0.5486	0.06385	0.03901	8	12	1.637	16.85
WT vs. +α-syn^{AAA}	0.6125	0.5911	0.02142	0.03684	8	10	0.5814	14.8
Row 17								
WT vs. +α-syn^{WT}	0.7044	0.6315	0.07284	0.03708	8	12	1.964	17.24
WT vs. +α-syn^{AAA}	0.7044	0.6724	0.03195	0.03216	8	10	0.9934	13.46
Row 18								
WT vs. +α-syn^{WT}	0.7676	0.6976	0.06993	0.03473	8	12	2.014	17.91
WT vs. +α-syn^{AAA}	0.7676	0.7394	0.02818	0.02939	8	10	0.9591	14.58
Row 19								
WT vs. +α-syn^{WT}	0.8321	0.7556	0.07648	0.02973	8	12	2.573	17.98
WT vs. +α-syn^{AAA}	0.8321	0.7923	0.03979	0.0253	8	10	1.573	15.11
Row 20								
WT vs. +α-syn^{WT}	0.8734	0.8207	0.05275	0.02494	8	12	2.115	17.72
WT vs. +α-syn^{AAA}	0.8734	0.844	0.02947	0.02043	8	10	1.442	15.83
Row 21								
WT vs. +α-syn^{WT}	0.9172	0.864	0.05321	0.02128	8	12	2.5	17.99
WT vs. +α-syn^{AAA}	0.9172	0.8851	0.0321	0.01929	8	10	1.664	15.96
Row 22								
WT vs. +α-syn^{WT}	0.9471	0.8997	0.04734	0.01596	8	12	2.966	18
WT vs. +α-syn^{AAA}	0.9471	0.9124	0.03465	0.01573	8	10	2.204	15.9
Row 23								
WT vs. +α-syn^{WT}	0.96	0.9402	0.01986	0.01085	8	12	1.831	17.11

WT vs. +α-syn^{AAA}	0.96	0.9448	0.01522	0.01051	8	10	1.448	14.79
Row 24								
WT vs. +α-syn^{WT}	0.9814	0.9682	0.01315	0.007977	8	12	1.649	17.84
WT vs. +α-syn^{AAA}	0.9814	0.9655	0.01587	0.008482	8	10	1.871	14.91
Row 25								
WT vs. +α-syn^{WT}	0.9876	0.9916	-0.003932	0.006982	8	12	0.5632	9.392
WT vs. +α-syn^{AAA}	0.9876	0.9921	-0.004479	0.007373	8	10	0.6074	11.1
Row 26								
WT vs. +α-syn^{WT}	0.9802	0.9979	-0.01771	0.008918	8	12	1.986	7.841
WT vs. +α-syn^{AAA}	0.9802	0.9895	-0.009312	0.01017	8	10	0.9154	11.97
Row 27								
WT vs. +α-syn^{WT}	0.929	0.9637	-0.03471	0.01423	8	12	2.439	11.77
WT vs. +α-syn^{AAA}	0.929	0.952	-0.02299	0.01389	8	10	1.655	10.83
Row 28								
WT vs. +α-syn^{WT}	0.8891	0.9468	-0.05776	0.01793	8	12	3.221	15
WT vs. +α-syn^{AAA}	0.8891	0.917	-0.02789	0.01609	8	10	1.734	11.37
Row 29								
WT vs. +α-syn^{WT}	0.8585	0.9197	-0.06121	0.02069	8	12	2.959	17.24
WT vs. +α-syn^{AAA}	0.8585	0.8794	-0.02091	0.01935	8	10	1.081	15.05
Row 30								
WT vs. +α-syn^{WT}	0.8329	0.8965	-0.06361	0.0196	8	12	3.245	17.59
WT vs. +α-syn^{AAA}	0.8329	0.851	-0.01809	0.01896	8	10	0.9541	15.78
Row 31								
WT vs. +α-syn^{WT}	0.8054	0.8749	-0.06952	0.02344	8	12	2.966	16.91
WT vs. +α-syn^{AAA}	0.8054	0.8318	-0.02643	0.02216	8	10	1.193	14.89
Row 32								
WT vs. +α-syn^{WT}	0.7873	0.853	-0.06565	0.02311	8	12	2.841	15.35
WT vs. +α-syn^{AAA}	0.7873	0.8095	-0.02214	0.02544	8	10	0.8702	15.83
Row 33								
WT vs. +α-syn^{WT}	0.7609	0.8361	-0.07523	0.02687	8	12	2.8	15.87
WT vs. +α-syn^{AAA}	0.7609	0.7894	-0.02848	0.02951	8	10	0.9654	15.93
Row 34								
WT vs. +α-syn^{WT}	0.7351	0.8207	-0.0856	0.02759	8	12	3.103	15.78
WT vs. +α-syn^{AAA}	0.7351	0.7617	-0.02658	0.03011	8	10	0.8828	15.88
Row 35								
WT vs. +α-syn^{WT}	0.7138	0.8082	-0.09438	0.02915	8	12	3.238	14.3
WT vs. +α-syn^{AAA}	0.7138	0.7482	-0.03434	0.0343	8	10	1.001	15.97

Row 36								
WT vs. +α-syn^{WT}	0.6993	0.7935	-0.09425	0.03183	8	12	2.961	13.08
WT vs. +α-syn^{AAA}	0.6993	0.7183	-0.01903	0.03846	8	10	0.4948	15.93
Row 37								
WT vs. +α-syn^{WT}	0.6794	0.7794	-0.1	0.03164	8	12	3.162	14.81
WT vs. +α-syn^{AAA}	0.6794	0.7049	-0.02547	0.03705	8	10	0.6875	15.99
Row 38								
WT vs. +α-syn^{WT}	0.6571	0.7687	-0.1116	0.03052	8	12	3.656	16.74
WT vs. +α-syn^{AAA}	0.6571	0.6734	-0.01631	0.03878	8	10	0.4204	15.2
Row 39								
WT vs. +α-syn^{WT}	0.629	0.7565	-0.1275	0.02789	7	12	4.57	16.16
WT vs. +α-syn^{AAA}	0.629	0.6753	-0.04629	0.03601	7	10	1.285	14.11
Row 40								
WT vs. +α-syn^{WT}	0.6266	0.7453	-0.1187	0.03823	8	12	3.106	12.83
WT vs. +α-syn^{AAA}	0.6266	0.6667	-0.0401	0.04467	8	10	0.8976	15.65
Row 41								
WT vs. +α-syn^{WT}	0.6088	0.7351	-0.1263	0.02988	8	12	4.227	17.87
WT vs. +α-syn^{AAA}	0.6088	0.657	-0.04823	0.03928	8	10	1.228	13.98
Row 42								
WT vs. +α-syn^{WT}	0.5772	0.7266	-0.1495	0.03206	8	12	4.662	16.83
WT vs. +α-syn^{AAA}	0.5772	0.6467	-0.06953	0.04024	8	10	1.728	15.26
Row 43								
WT vs. +α-syn^{WT}	0.5556	0.7108	-0.1551	0.0376	8	12	4.126	15.03
WT vs. +α-syn^{AAA}	0.5556	0.6345	-0.07883	0.04606	8	10	1.712	15.93
Row 44								
WT vs. +α-syn^{WT}	0.5422	0.7037	-0.1616	0.04095	8	12	3.945	15.25
WT vs. +α-syn^{AAA}	0.5422	0.623	-0.08085	0.04638	8	10	1.743	15.95
Row 45								
WT vs. +α-syn^{WT}	0.5505	0.6921	-0.1416	0.03685	8	12	3.843	17.74
WT vs. +α-syn^{AAA}	0.5505	0.6142	-0.06366	0.04355	8	10	1.462	15.16
Row 46								
WT vs. +α-syn^{WT}	0.5435	0.6898	-0.1464	0.03637	8	12	4.025	17.97
WT vs. +α-syn^{AAA}	0.5435	0.6006	-0.05707	0.04245	8	10	1.344	14.38
Row 47								
WT vs. +α-syn^{WT}	0.5236	0.6731	-0.1495	0.03752	8	12	3.985	17.54
WT vs. +α-syn^{AAA}	0.5236	0.594	-0.07034	0.04571	8	10	1.539	15.08

Row 48								
WT vs. +α-syn^{WT}	0.5153	0.6766	-0.1612	0.04034	8	12	3.997	17.32
WT vs. +α-syn^{AAA}	0.5153	0.5811	-0.06575	0.0467	8	10	1.408	15.64
Row 49								
WT vs. +α-syn^{WT}	0.5033	0.6711	-0.1678	0.04129	8	12	4.063	16.96
WT vs. +α-syn^{AAA}	0.5033	0.5682	-0.06491	0.04566	8	10	1.422	15.97
Row 50								
WT vs. +α-syn^{WT}	0.4969	0.6579	-0.161	0.04305	8	12	3.741	15.62
WT vs. +α-syn^{AAA}	0.4969	0.5613	-0.06442	0.04693	8	10	1.373	15.84
Row 51								
WT vs. +α-syn^{WT}	0.4905	0.6497	-0.1591	0.04244	8	12	3.75	15.61
WT vs. +α-syn^{AAA}	0.4905	0.5511	-0.0606	0.04663	8	10	1.3	15.88
Row 52								
WT vs. +α-syn^{WT}	0.4791	0.6474	-0.1683	0.04346	8	12	3.873	16.81
WT vs. +α-syn^{AAA}	0.4791	0.55	-0.07092	0.04445	8	10	1.596	15.78
Row 53								
WT vs. +α-syn^{WT}	0.4684	0.639	-0.1706	0.04122	8	12	4.139	16.89
WT vs. +α-syn^{AAA}	0.4684	0.537	-0.06863	0.04414	8	10	1.555	15.99
Row 54								
WT vs. +α-syn^{WT}	0.4627	0.631	-0.1683	0.0412	8	12	4.085	16.46
WT vs. +α-syn^{AAA}	0.4627	0.5283	-0.06562	0.04379	8	10	1.499	15.9
Row 55								
WT vs. +α-syn^{WT}	0.4567	0.6218	-0.1651	0.04124	8	12	4.004	15.61
WT vs. +α-syn^{AAA}	0.4567	0.5179	-0.06119	0.04512	8	10	1.356	15.86
Row 56								
WT vs. +α-syn^{WT}	0.4532	0.6214	-0.1682	0.03996	8	12	4.21	16
WT vs. +α-syn^{AAA}	0.4532	0.5123	-0.05916	0.04377	8	10	1.351	15.94
Row 57								
WT vs. +α-syn^{WT}	0.445	0.616	-0.171	0.04336	8	12	3.943	15.34
WT vs. +α-syn^{AAA}	0.445	0.5072	-0.06214	0.04486	8	10	1.385	15.18
Row 58								
WT vs. +α-syn^{WT}	0.4347	0.6123	-0.1776	0.04137	8	12	4.294	16.82
WT vs. +α-syn^{AAA}	0.4347	0.5011	-0.06639	0.04188	8	10	1.585	15.71
Row 59								
WT vs. +α-syn^{WT}	0.4291	0.5998	-0.1708	0.03876	8	12	4.407	17.26
WT vs. +α-syn^{AAA}	0.4291	0.491	-0.06191	0.04009	8	10	1.544	15.97
Row 60								

WT vs. +α-syn^{WT}	0.4156	0.5959	-0.1803	0.04241	8	12	4.251	16.27
WT vs. +α-syn^{AAA}	0.4156	0.4895	-0.07392	0.04239	8	10	1.744	15.24
Row 61								
WT vs. +α-syn^{WT}	0.4105	0.5847	-0.1742	0.03715	8	12	4.691	16.5
WT vs. +α-syn^{AAA}	0.4105	0.4832	-0.07277	0.03883	8	10	1.874	15.82
Row 62								
WT vs. +α-syn^{WT}	0.4072	0.5845	-0.1772	0.03969	8	12	4.465	17.35
WT vs. +α-syn^{AAA}	0.4072	0.4784	-0.07116	0.03896	8	10	1.826	15.74
Row 63								
WT vs. +α-syn^{WT}	0.397	0.579	-0.182	0.04147	8	12	4.389	16.48
WT vs. +α-syn^{AAA}	0.397	0.4737	-0.07666	0.0397	8	10	1.931	14.69
Row 64								
WT vs. +α-syn^{WT}	0.3871	0.5709	-0.1837	0.04412	8	12	4.165	15.51
WT vs. +α-syn^{AAA}	0.3871	0.4726	-0.0855	0.04179	8	10	2.046	13.5
Row 65								
WT vs. +α-syn^{WT}	0.4068	0.5709	-0.1642	0.04773	7	12	3.439	12.81
WT vs. +α-syn^{AAA}	0.4068	0.4615	-0.05478	0.04438	7	10	1.234	10.42
Row 66								
WT vs. +α-syn^{WT}	0.3759	0.5572	-0.1812	0.04533	8	12	3.998	15.01
WT vs. +α-syn^{AAA}	0.3759	0.4648	-0.0889	0.04255	8	10	2.089	12.74
Row 67								
WT vs. +α-syn^{WT}	0.3833	0.5553	-0.1719	0.04379	8	12	3.926	16.45
WT vs. +α-syn^{AAA}	0.3833	0.46	-0.0767	0.04026	8	10	1.905	13.77
Row 68								
WT vs. +α-syn^{WT}	0.3802	0.5489	-0.1687	0.04296	8	12	3.926	16.35
WT vs. +α-syn^{AAA}	0.3802	0.453	-0.07273	0.04019	8	10	1.809	14.07
Row 69								
WT vs. +α-syn^{WT}	0.3788	0.5429	-0.1641	0.04262	8	12	3.85	16.83
WT vs. +α-syn^{AAA}	0.3788	0.4456	-0.06685	0.03933	8	10	1.7	14.32
Row 70								
WT vs. +α-syn^{WT}	0.3713	0.5338	-0.1625	0.04128	8	12	3.936	16.91
WT vs. +α-syn^{AAA}	0.3713	0.4423	-0.07101	0.03824	8	10	1.857	14.5
Row 71								
WT vs. +α-syn^{WT}	0.3646	0.5389	-0.1744	0.04252	8	12	4.101	16.98
WT vs. +α-syn^{AAA}	0.3646	0.4395	-0.07499	0.0381	8	10	1.968	13.85
Row 72								
WT vs. +α-syn^{WT}	0.3525	0.5289	-0.1764	0.04266	8	12	4.134	16.99

WT vs. +α-syn^{AAA}	0.3525	0.4341	-0.08155	0.0392	8	10	2.08	14.44
Row 73								
WT vs. +α-syn^{WT}	0.3458	0.5245	-0.1787	0.04205	8	12	4.25	17.12
WT vs. +α-syn^{AAA}	0.3458	0.428	-0.08222	0.03852	8	10	2.135	14.53
Row 74								
WT vs. +α-syn^{WT}	0.3349	0.5164	-0.1816	0.0437	8	12	4.155	16.42
WT vs. +α-syn^{AAA}	0.3349	0.434	-0.09912	0.04007	8	10	2.473	13.67
Row 75								
WT vs. +α-syn^{WT}	0.3389	0.5121	-0.1732	0.04108	8	12	4.216	17.52
WT vs. +α-syn^{AAA}	0.3389	0.4286	-0.08968	0.03657	8	10	2.452	14.57
Row 76								
WT vs. +α-syn^{WT}	0.3366	0.5075	-0.1709	0.04132	8	12	4.137	17.63
WT vs. +α-syn^{AAA}	0.3366	0.4218	-0.08518	0.03746	8	10	2.274	15.1
Row 77								
WT vs. +α-syn^{WT}	0.3298	0.507	-0.1772	0.04255	8	12	4.164	17.59
WT vs. +α-syn^{AAA}	0.3298	0.4199	-0.0901	0.03795	8	10	2.375	14.74
Row 78								
WT vs. +α-syn^{WT}	0.3171	0.5038	-0.1867	0.04253	8	12	4.391	17.76
WT vs. +α-syn^{AAA}	0.3171	0.4143	-0.09718	0.03694	8	10	2.631	14.61
Row 79								
WT vs. +α-syn^{WT}	0.3166	0.4972	-0.1806	0.04113	8	12	4.39	17.76
WT vs. +α-syn^{AAA}	0.3166	0.4102	-0.09357	0.03719	8	10	2.516	15.28
Row 80								
WT vs. +α-syn^{WT}	0.3102	0.4901	-0.1799	0.04337	8	12	4.149	17.67
WT vs. +α-syn^{AAA}	0.3102	0.4159	-0.1057	0.03762	8	10	2.81	14.35
Row 81								
WT vs. +α-syn^{WT}	0.3163	0.4924	-0.1761	0.04316	8	12	4.08	17.84
WT vs. +α-syn^{AAA}	0.3163	0.4064	-0.0901	0.03795	8	10	2.374	15.05
Row 82								
WT vs. +α-syn^{WT}	0.317	0.488	-0.171	0.04565	8	12	3.746	16.8
WT vs. +α-syn^{AAA}	0.317	0.4044	-0.0874	0.04041	8	10	2.163	13.27
Row 83								
WT vs. +α-syn^{WT}	0.3029	0.4809	-0.178	0.04541	8	12	3.92	17.25
WT vs. +α-syn^{AAA}	0.3029	0.4009	-0.09808	0.03917	8	10	2.504	13.33
Row 84								
WT vs. +α-syn^{WT}	0.3099	0.4775	-0.1676	0.04762	8	12	3.519	17.05
WT vs. +α-syn^{AAA}	0.3099	0.3953	-0.08541	0.04176	8	10	2.045	13.42

Row 85								
WT vs. +α-syn^{WT}	0.303	0.4788	-0.1758	0.04781	8	12	3.676	16.46
WT vs. +α-syn^{AAA}	0.303	0.3935	-0.09053	0.0429	8	10	2.11	13.16
Row 86								
WT vs. +α-syn^{WT}	0.2981	0.4725	-0.1744	0.04855	8	12	3.591	16.55
WT vs. +α-syn^{AAA}	0.2981	0.3672	-0.06907	0.04271	8	10	1.617	12.74
Row 87								
WT vs. +α-syn^{WT}	0.3033	0.4701	-0.1668	0.04731	8	12	3.526	16.8
WT vs. +α-syn^{AAA}	0.3033	0.3814	-0.0781	0.04165	8	10	1.875	13.12
Row 88								
WT vs. +α-syn^{WT}	0.2946	0.4665	-0.1719	0.04739	8	12	3.627	16.84
WT vs. +α-syn^{AAA}	0.2946	0.3851	-0.09046	0.04129	8	10	2.191	12.9
Row 89								
WT vs. +α-syn^{WT}	0.2894	0.4691	-0.1798	0.04892	8	12	3.674	17.22
WT vs. +α-syn^{AAA}	0.2894	0.377	-0.08764	0.04149	8	10	2.112	12.82
Row 90								
WT vs. +α-syn^{WT}	0.2912	0.4658	-0.1747	0.05034	8	12	3.47	17.35
WT vs. +α-syn^{AAA}	0.2912	0.3765	-0.08535	0.04235	8	10	2.015	12.86
Row 91								
WT vs. +α-syn^{WT}	0.2907	0.4589	-0.1682	0.04931	8	12	3.411	16.4
WT vs. +α-syn^{AAA}	0.2907	0.3704	-0.07972	0.04324	8	10	1.844	12.41
Row 92								
WT vs. +α-syn^{WT}	0.2798	0.4605	-0.1807	0.04768	8	12	3.79	16.77
WT vs. +α-syn^{AAA}	0.2798	0.369	-0.0892	0.04097	8	10	2.177	12.39
Row 93								
WT vs. +α-syn^{WT}	0.2794	0.4604	-0.181	0.04925	8	12	3.675	16.85
WT vs. +α-syn^{AAA}	0.2794	0.3618	-0.08241	0.04181	8	10	1.971	12.16
Row 94								
WT vs. +α-syn^{WT}	0.2753	0.4493	-0.1739	0.04938	8	12	3.522	16.42
WT vs. +α-syn^{AAA}	0.2753	0.362	-0.0867	0.04166	8	10	2.081	11.26
Row 95								
WT vs. +α-syn^{WT}	0.2745	0.4551	-0.1806	0.05024	8	12	3.594	16.95
WT vs. +α-syn^{AAA}	0.2745	0.3549	-0.08039	0.04057	8	10	1.981	10.78
Row 96								
WT vs. +α-syn^{WT}	0.2711	0.4556	-0.1845	0.04831	8	12	3.819	17.22
WT vs. +α-syn^{AAA}	0.2711	0.3494	-0.07831	0.03986	8	10	1.965	12

Row 97								
WT vs. +α-syn^{WT}	0.2735	0.4458	-0.1723	0.04846	8	12	3.555	16.95
WT vs. +α-syn^{AAA}	0.2735	0.3368	-0.06324	0.03995	8	10	1.583	11.44
Row 98								
WT vs. +α-syn^{WT}	0.2679	0.4465	-0.1785	0.0497	8	12	3.593	17.5
WT vs. +α-syn^{AAA}	0.2679	0.3458	-0.07789	0.03868	8	10	2.014	10.87
Row 99								
WT vs. +α-syn^{WT}	0.2545	0.4435	-0.189	0.04925	8	12	3.837	17.21
WT vs. +α-syn^{AAA}	0.2545	0.337	-0.08247	0.04004	8	10	2.06	11.54
Row 100								
WT vs. +α-syn^{WT}	0.2582	0.4395	-0.1813	0.0495	8	12	3.663	17.74
WT vs. +α-syn^{AAA}	0.2582	0.3376	-0.07939	0.0385	8	10	2.062	11.67
Row 101								
WT vs. +α-syn^{WT}	0.252	0.435	-0.1829	0.04859	8	12	3.764	17.22
WT vs. +α-syn^{AAA}	0.252	0.3293						

**FIGURE 5C**	WT	+α-syn^{WT}	+α-syn^{AAA}
Number of values	8	12	10
Mean	0.3729	0.3227	0.352
Std. Deviation	0.07218	0.1206	0.09492
Std. Error of Mean	0.02552	0.03482	0.03002
	WT	+α-syn^{WT}	+α-syn^{AAA}
Test for normal distribution			
Shapiro-Wilk test			
W	0.9503	0.9434	0.9476
P value	0.7146	0.5432	0.6405
Passed normality test (alpha=0.05)?	Yes	Yes	Yes
P value summary	ns	ns	ns

**FIGURE 5C**	
ANOVA summary	
F	0.6149
P value	0.5481
P value summary	ns
Significant diff. among means (P < 0.05)?	No
R squared	0.04356
Brown-Forsythe test	
F (DFn, DFd)	2.280 (2, 27)
P value	0.1216
P value summary	ns
Are SDs significantly different (P < 0.05)?	No
Bartlett's test	
Bartlett's statistic (corrected)	2.024
P value	0.3635
P value summary	ns
Are SDs significantly different (P < 0.05)?	No

**FIGURE 5C**					
ANOVA table	SS	DF	MS	F (DFn, DFd)	P value
Treatment (between columns)	0.01265	2	0.006323	F (2, 27) = 0.6149	P=0.5481
Residual (within columns)	0.2776	27	0.01028		
Total	0.2903	29			
Data summary					
Number of treatments (columns)	3				
Number of values (total)	30				
Number of families	1				
Number of comparisons per family	2				
Alpha	0.05				

**FIGURE 5C**								
Dunnett's multiple comparisons test	Mean Diff.	95.00% CI of diff.	Below threshold?	Summary	Adjusted P Value	A-?		
WT vs. +α-syn^{WT}	0.05017	-0.05729 to 0.1576	No	ns	0.4476	B	+α-syn^{WT}	
WT vs. +α-syn^{AAA}	0.02088	-0.09079 to 0.1326	No	ns	0.8667	C	+α-syn^{AAA}	
Test details	Mean 1	Mean 2	Mean Diff.	SE of diff.	n1	n2	q	DF
WT vs. +α-syn^{WT}	0.3729	0.3227	0.05017	0.04629	8	12	1.084	27
WT vs. +α-syn^{AAA}	0.3729	0.352	0.02088	0.0481	8	10	0.4341	27

**FIGURE 5D**	WT	+α-syn^{WT}	+α-syn^{AAA}
Number of values	8	12	10
Mean	0.05897	0.05282	0.05651
Std. Deviation	0.007815	0.008831	0.007349
Std. Error of Mean	0.002763	0.002549	0.002324
	WT	+α-syn^{WT}	+α-syn^{AAA}
Test for normal distribution			
Shapiro-Wilk test			
W	0.8326	0.9494	0.9507
P value	0.0632	0.6283	0.677
Passed normality test (alpha=0.05)?	Yes	Yes	Yes
P value summary	ns	ns	ns

**FIGURE 5D**	
ANOVA summary	
F	1.46
P value	0.2499
P value summary	ns
Significant diff. among means (P < 0.05)?	No
R squared	0.09762
Brown-Forsythe test	
F (DFn, DFd)	0.3846 (2, 27)
P value	0.6844
P value summary	ns
Are SDs significantly different (P < 0.05)?	No
Bartlett's test	
Bartlett's statistic (corrected)	0.3345
P value	0.846
P value summary	ns
Are SDs significantly different (P < 0.05)?	No

**FIGURE 5D**					
ANOVA table	SS	DF	MS	F (DFn, DFd)	P value
Treatment (between columns)	0.0001916	2	0.00009582	F (2, 27) = 1.460	P=0.2499
Residual (within columns)	0.001771	27	0.00006561		
Total	0.001963	29			
Data summary					
Number of treatments (columns)	3				
Number of values (total)	30				
Number of families	1				
Number of comparisons per family	2				
Alpha	0.05				

**FIGURE 5D**								
Dunnett's multiple comparisons test	Mean Diff.	95.00% CI of diff.	Below threshold?	Summary	Adjusted P Value	A-?		
WT vs. +α-syn^{WT}	0.006149	-0.002434 to 0.01473	No	ns	0.1815	B	+α-syn^{WT}	
WT vs. +α-syn^{AAA}	0.002456	-0.006464 to 0.01138	No	ns	0.7395	C	+α-syn^{AAA}	
Test details	Mean 1	Mean 2	Mean Diff.	SE of diff.	n1	n2	q	DF
WT vs. +α-syn^{WT}	0.05897	0.05282	0.006149	0.003697	8	12	1.663	27
WT vs. +α-syn^{AAA}	0.05897	0.05651	0.002456	0.003842	8	10	0.6392	27

**FIGURE 5E**	WT	+α-syn^{WT}	+α-syn^{AAA}
Number of values	8	12	10
Mean	74.8	56.5	67.07
Std. Deviation	9.651	11.99	6.407
Std. Error of Mean	3.412	3.46	2.026
	WT	+α-syn^{WT}	+α-syn^{AAA}
Test for normal distribution			
Shapiro-Wilk test			
W	0.9854	0.9695	0.9225
P value	0.9847	0.9053	0.3779
Passed normality test (alpha=0.05)?	Yes	Yes	Yes
P value summary	ns	ns	ns

**FIGURE 5E**	
ANOVA summary	
F	8.699
P value	0.0012
P value summary	**
Significant diff. among means (P < 0.05)?	Yes
R squared	0.3919
Brown-Forsythe test	
F (DFn, DFd)	0.8958 (2, 27)
P value	0.4201
P value summary	ns
Are SDs significantly different (P < 0.05)?	No
Bartlett's test	
Bartlett's statistic (corrected)	3.353
P value	0.187
P value summary	ns
Are SDs significantly different (P < 0.05)?	No

**FIGURE 5E**					
ANOVA table	SS	DF	MS	F (DFn, DFd)	P value
Treatment (between columns)	1676	2	838.2	F (2, 27) = 8.699	P=0.0012
Residual (within columns)	2602	27	96.36		
Total	4278	29			
Data summary					
Number of treatments (columns)	3				
Number of values (total)	30				
Number of families	1				
Number of comparisons per family	2				
Alpha	0.05				

**FIGURE 5E**								
Dunnett's multiple comparisons test	Mean Diff.	95.00% CI of diff.	Below threshold?	Summary	Adjusted P Value	A-?		
WT vs. +α-syn^{WT}	18.29	7.890 to 28.69	Yes	***	0.0007	B	+α-syn^{WT}	
WT vs. +α-syn^{AAA}	7.727	-3.083 to 18.54	No	ns	0.1827	C	+α-syn^{AAA}	
Test details	Mean 1	Mean 2	Mean Diff.	SE of diff.	n1	n2	q	DF
WT vs. +α-syn^{WT}	74.8	56.5	18.29	4.481	8	12	4.083	27
WT vs. +α-syn^{AAA}	74.8	67.07	7.727	4.656	8	10	1.659	27

**FIGURE 6B**					
Two-way RM ANOVA	Matching Stacked				
Assume sphericity?	No				
Alpha	0.05				
Source of Variation	% of total variation	P value	P value summary	Significant?	Geisser-Greenhouse's epsilon
Time x Column Factor	2.997	<0.0001	****	Yes	
Time	77.3	<0.0001	****	Yes	0.02706
Column Factor	0.6924	0.4377	ns	No	
Subject	7.619	<0.0001	****	Yes	
ANOVA table	SS	DF	MS	F (DFn, DFd)	P value
Time x Column Factor	0.5068	200	0.002534	F (200, 1900) = 2.238	P<0.0001
Time	13.07	100	0.1307	F (2.706, 51.42) = 115.5	P<0.0001
Column Factor	0.1171	2	0.05854	F (2, 19) = 0.8633	P=0.4377
Subject	1.288	19	0.06781	F (19, 1900) = 59.89	P<0.0001
Residual	2.151	1900	0.001132		

**FIGURE 6B**	
Data summary	
Number of columns (Column Factor)	3
Number of rows (Time)	101
Number of subjects (Subject)	22
Number of missing values	0
Within each row, compare columns (simple effects within rows)	
Number of families	101
Number of comparisons per family	2
Alpha	0.05

**FIGURE 6B**					
Dunnett's multiple comparisons test	Mean Diff.	95.00% CI of diff.	Below threshold?	Summary	Adjusted P Value
Row 1					
WT vs. +α-syn^{WT}	0.0005594	-0.02931 to 0.03042	No	ns	0.9984
WT vs. +α-syn^{AAA}	-0.01739	-0.05780 to 0.02302	No	ns	0.4685
Row 2					
WT vs. +α-syn^{WT}	0.006868	-0.01241 to 0.02614	No	ns	0.5717
WT vs. +α-syn^{AAA}	-0.01418	-0.04108 to 0.01272	No	ns	0.3411
Row 3					
WT vs. +α-syn^{WT}	-0.01254	-0.03324 to 0.008172	No	ns	0.2295
WT vs. +α-syn^{AAA}	-0.03492	-0.06571 to -0.004126	Yes	*	0.0276

Row 4					
WT vs. +α-syn^{WT}	0.004258	-0.01430 to 0.02282	No	ns	0.7945
WT vs. +α-syn^{AAA}	-0.0303	-0.05872 to -0.001886	Yes	*	0.038
Row 5					
WT vs. +α-syn^{WT}	-0.0007538	-0.01395 to 0.01244	No	ns	0.9853
WT vs. +α-syn^{AAA}	-0.003766	-0.02981 to 0.02228	No	ns	0.8981
Row 6					
WT vs. +α-syn^{WT}	0.007801	-0.01245 to 0.02805	No	ns	0.5393
WT vs. +α-syn^{AAA}	0.003329	-0.02196 to 0.02862	No	ns	0.917
Row 7					
WT vs. +α-syn^{WT}	0.003881	-0.01383 to 0.02159	No	ns	0.8051
WT vs. +α-syn^{AAA}	0.005359	-0.01036 to 0.02108	No	ns	0.6061
Row 8					
WT vs. +α-syn^{WT}	-0.003531	-0.01553 to 0.008468	No	ns	0.6861
WT vs. +α-syn^{AAA}	0.009073	-0.01989 to 0.03804	No	ns	0.6117
Row 9					
WT vs. +α-syn^{WT}	0.002567	-0.01923 to 0.02436	No	ns	0.9389
WT vs. +α-syn^{AAA}	-0.003005	-0.02574 to 0.01973	No	ns	0.9214
Row 10					
WT vs. +α-syn^{WT}	-0.01072	-0.02555 to 0.004111	No	ns	0.1603
WT vs. +α-syn^{AAA}	-0.01476	-0.03697 to 0.007464	No	ns	0.1878
Row 11					
WT vs. +α-syn^{WT}	0.002923	-0.02487 to 0.03071	No	ns	0.9513
WT vs. +α-syn^{AAA}	0.0002667	-0.02594 to 0.02647	No	ns	0.9995
Row 12					
WT vs. +α-syn^{WT}	0.02143	-0.03007 to 0.07294	No	ns	0.4793
WT vs. +α-syn^{AAA}	0.02306	-0.02993 to 0.07605	No	ns	0.4569
Row 13					
WT vs. +α-syn^{WT}	0.03723	-0.03888 to 0.1134	No	ns	0.3829
WT vs. +α-syn^{AAA}	0.01381	-0.06509 to 0.09272	No	ns	0.8681
Row 14					
WT vs. +α-syn^{WT}	0.0356	-0.04756 to 0.1188	No	ns	0.4641
WT vs. +α-syn^{AAA}	-0.000402	-0.08204 to 0.08124	No	ns	0.9999
Row 15					
WT vs. +α-syn^{WT}	0.0625	-0.03618 to 0.1612	No	ns	0.226
WT vs. +α-syn^{AAA}	-0.006967	-0.1072 to 0.09324	No	ns	0.9769

Row 16					
WT vs. +α-syn^{WT}	0.06723	-0.04405 to 0.1785	No	ns	0.2559
WT vs. +α-syn^{AAA}	0.007744	-0.1005 to 0.1160	No	ns	0.975
Row 17					
WT vs. +α-syn^{WT}	0.06122	-0.04896 to 0.1714	No	ns	0.3065
WT vs. +α-syn^{AAA}	-0.001197	-0.1082 to 0.1058	No	ns	0.9994
Row 18					
WT vs. +α-syn^{WT}	0.06182	-0.03254 to 0.1562	No	ns	0.2107
WT vs. +α-syn^{AAA}	0.0007016	-0.08907 to 0.09048	No	ns	0.9997
Row 19					
WT vs. +α-syn^{WT}	0.04691	-0.05896 to 0.1528	No	ns	0.439
WT vs. +α-syn^{AAA}	-0.004336	-0.1077 to 0.09900	No	ns	0.9907
Row 20					
WT vs. +α-syn^{WT}	0.07944	-0.01548 to 0.1744	No	ns	0.1009
WT vs. +α-syn^{AAA}	0.02134	-0.06949 to 0.1122	No	ns	0.769
Row 21					
WT vs. +α-syn^{WT}	0.08111	-0.01226 to 0.1745	No	ns	0.087
WT vs. +α-syn^{AAA}	0.006942	-0.08846 to 0.1023	No	ns	0.9747
Row 22					
WT vs. +α-syn^{WT}	0.07768	-0.008680 to 0.1640	No	ns	0.0772
WT vs. +α-syn^{AAA}	0.005809	-0.07593 to 0.08754	No	ns	0.9737
Row 23					
WT vs. +α-syn^{WT}	0.07108	-0.005163 to 0.1473	No	ns	0.0668
WT vs. +α-syn^{AAA}	0.01587	-0.05817 to 0.08992	No	ns	0.7986
Row 24					
WT vs. +α-syn^{WT}	0.07354	-0.004252 to 0.1513	No	ns	0.0638
WT vs. +α-syn^{AAA}	0.0009556	-0.07036 to 0.07228	No	ns	0.9991
Row 25					
WT vs. +α-syn^{WT}	0.07187	-0.002455 to 0.1462	No	ns	0.058
WT vs. +α-syn^{AAA}	0.004344	-0.06341 to 0.07210	No	ns	0.9803
Row 26					
WT vs. +α-syn^{WT}	0.06966	0.0002897 to 0.1390	Yes	*	0.049
WT vs. +α-syn^{AAA}	-0.005447	-0.07954 to 0.06865	No	ns	0.9751
Row 27					
WT vs. +α-syn^{WT}	0.06315	-0.0004421 to 0.1267	No	ns	0.0516
WT vs. +α-syn^{AAA}	0.01443	-0.04409 to 0.07294	No	ns	0.7616
Row 28					

WT vs. +α-syn^{WT}	0.0542	-0.007208 to 0.1156	No	ns	0.0844
WT vs. +α-syn^{AAA}	0.01017	-0.03932 to 0.05966	No	ns	0.8193
Row 29					
WT vs. +α-syn^{WT}	0.06327	0.001574 to 0.1250	Yes	*	0.0445
WT vs. +α-syn^{AAA}	0.01397	-0.03684 to 0.06478	No	ns	0.695
Row 30					
WT vs. +α-syn^{WT}	0.0521	-0.01235 to 0.1165	No	ns	0.1161
WT vs. +α-syn^{AAA}	0.00476	-0.04775 to 0.05727	No	ns	0.9623
Row 31					
WT vs. +α-syn^{WT}	0.03501	-0.01645 to 0.08647	No	ns	0.1969
WT vs. +α-syn^{AAA}	0.01075	-0.03140 to 0.05290	No	ns	0.7403
Row 32					
WT vs. +α-syn^{WT}	0.05202	-0.006028 to 0.1101	No	ns	0.0782
WT vs. +α-syn^{AAA}	0.02543	-0.008576 to 0.05943	No	ns	0.1463
Row 33					
WT vs. +α-syn^{WT}	0.03935	-0.007017 to 0.08571	No	ns	0.0974
WT vs. +α-syn^{AAA}	0.008557	-0.02826 to 0.04538	No	ns	0.7867
Row 34					
WT vs. +α-syn^{WT}	0.03354	-0.02071 to 0.08779	No	ns	0.2483
WT vs. +α-syn^{AAA}	0.02615	-0.01066 to 0.06295	No	ns	0.1592
Row 35					
WT vs. +α-syn^{WT}	0.03841	-0.01837 to 0.09519	No	ns	0.1928
WT vs. +α-syn^{AAA}	0.0241	-0.007220 to 0.05542	No	ns	0.1324
Row 36					
WT vs. +α-syn^{WT}	0.03834	-0.006051 to 0.08274	No	ns	0.0916
WT vs. +α-syn^{AAA}	0.01869	-0.01747 to 0.05485	No	ns	0.3534
Row 37					
WT vs. +α-syn^{WT}	0.03737	-0.01862 to 0.09336	No	ns	0.2063
WT vs. +α-syn^{AAA}	0.02066	-0.02142 to 0.06275	No	ns	0.3805
Row 38					
WT vs. +α-syn^{WT}	0.0276	-0.03340 to 0.08859	No	ns	0.4453
WT vs. +α-syn^{AAA}	0.01363	-0.03313 to 0.06039	No	ns	0.6838
Row 39					
WT vs. +α-syn^{WT}	0.01919	-0.04315 to 0.08153	No	ns	0.6689
WT vs. +α-syn^{AAA}	0.006311	-0.03881 to 0.05143	No	ns	0.9085
Row 40					
WT vs. +α-syn^{WT}	0.02633	-0.02755 to 0.08020	No	ns	0.3969

WT vs. +α-syn^{AAA}	0.01829	-0.02474 to 0.06132	No	ns	0.4616
Row 41					
WT vs. +α-syn^{WT}	0.01357	-0.04341 to 0.07055	No	ns	0.7816
WT vs. +α-syn^{AAA}	0.00609	-0.04045 to 0.05263	No	ns	0.9222
Row 42					
WT vs. +α-syn^{WT}	0.02082	-0.03680 to 0.07844	No	ns	0.5831
WT vs. +α-syn^{AAA}	0.01907	-0.02416 to 0.06230	No	ns	0.4412
Row 43					
WT vs. +α-syn^{WT}	0.03202	-0.03207 to 0.09610	No	ns	0.3827
WT vs. +α-syn^{AAA}	0.02445	-0.02615 to 0.07506	No	ns	0.3807
Row 44					
WT vs. +α-syn^{WT}	0.02231	-0.03698 to 0.08160	No	ns	0.5605
WT vs. +α-syn^{AAA}	0.01703	-0.03237 to 0.06644	No	ns	0.5974
Row 45					
WT vs. +α-syn^{WT}	0.01158	-0.05083 to 0.07400	No	ns	0.8587
WT vs. +α-syn^{AAA}	0.02229	-0.02860 to 0.07319	No	ns	0.4554
Row 46					
WT vs. +α-syn^{WT}	0.014	-0.03939 to 0.06739	No	ns	0.7438
WT vs. +α-syn^{AAA}	0.02206	-0.02446 to 0.06858	No	ns	0.4107
Row 47					
WT vs. +α-syn^{WT}	0.01008	-0.04969 to 0.06985	No	ns	0.8814
WT vs. +α-syn^{AAA}	0.007992	-0.03915 to 0.05514	No	ns	0.869
Row 48					
WT vs. +α-syn^{WT}	0.003502	-0.05554 to 0.06254	No	ns	0.9842
WT vs. +α-syn^{AAA}	0.008624	-0.03982 to 0.05707	No	ns	0.8605
Row 49					
WT vs. +α-syn^{WT}	0.01768	-0.04217 to 0.07753	No	ns	0.6908
WT vs. +α-syn^{AAA}	0.02833	-0.02102 to 0.07768	No	ns	0.2823
Row 50					
WT vs. +α-syn^{WT}	0.007842	-0.04520 to 0.06088	No	ns	0.9063
WT vs. +α-syn^{AAA}	0.01747	-0.02948 to 0.06441	No	ns	0.5436
Row 51					
WT vs. +α-syn^{WT}	0.01214	-0.05185 to 0.07613	No	ns	0.8528
WT vs. +α-syn^{AAA}	0.01504	-0.03286 to 0.06295	No	ns	0.6303
Row 52					
WT vs. +α-syn^{WT}	0.01017	-0.04946 to 0.06979	No	ns	0.8769
WT vs. +α-syn^{AAA}	0.02173	-0.03321 to 0.07668	No	ns	0.5087

Row 53					
WT vs. +α-syn^{WT}	0.0008605	-0.06011 to 0.06183	No	ns	0.9991
WT vs. +α-syn^{AAA}	0.0212	-0.02835 to 0.07076	No	ns	0.4552
Row 54					
WT vs. +α-syn^{WT}	0.001786	-0.05402 to 0.05759	No	ns	0.9953
WT vs. +α-syn^{AAA}	0.003323	-0.04431 to 0.05095	No	ns	0.9775
Row 55					
WT vs. +α-syn^{WT}	0.009773	-0.05474 to 0.07428	No	ns	0.9027
WT vs. +α-syn^{AAA}	0.01648	-0.03562 to 0.06857	No	ns	0.6296
Row 56					
WT vs. +α-syn^{WT}	-0.007911	-0.06380 to 0.04797	No	ns	0.9126
WT vs. +α-syn^{AAA}	0.01219	-0.04013 to 0.06450	No	ns	0.7767
Row 57					
WT vs. +α-syn^{WT}	0.002316	-0.05611 to 0.06074	No	ns	0.9928
WT vs. +α-syn^{AAA}	0.02494	-0.02771 to 0.07758	No	ns	0.4051
Row 58					
WT vs. +α-syn^{WT}	-0.004441	-0.05740 to 0.04852	No	ns	0.9685
WT vs. +α-syn^{AAA}	0.02612	-0.02182 to 0.07405	No	ns	0.3167
Row 59					
WT vs. +α-syn^{WT}	0.003572	-0.04859 to 0.05573	No	ns	0.9788
WT vs. +α-syn^{AAA}	0.02285	-0.02674 to 0.07243	No	ns	0.4257
Row 60					
WT vs. +α-syn^{WT}	-0.001933	-0.05114 to 0.04727	No	ns	0.993
WT vs. +α-syn^{AAA}	0.009918	-0.03302 to 0.05286	No	ns	0.7836
Row 61					
WT vs. +α-syn^{WT}	0.01169	-0.05247 to 0.07585	No	ns	0.8625
WT vs. +α-syn^{AAA}	0.02157	-0.03362 to 0.07675	No	ns	0.4924
Row 62					
WT vs. +α-syn^{WT}	-0.01184	-0.07204 to 0.04837	No	ns	0.8434
WT vs. +α-syn^{AAA}	0.004681	-0.04597 to 0.05533	No	ns	0.9607
Row 63					
WT vs. +α-syn^{WT}	-0.002157	-0.05688 to 0.05257	No	ns	0.9929
WT vs. +α-syn^{AAA}	0.01862	-0.03423 to 0.07147	No	ns	0.5893
Row 64					
WT vs. +α-syn^{WT}	-0.01254	-0.06372 to 0.03865	No	ns	0.7711
WT vs. +α-syn^{AAA}	0.01857	-0.02749 to 0.06463	No	ns	0.5103

Row 65					
WT vs. +α-syn^{WT}	0.002804	-0.05246 to 0.05807	No	ns	0.9883
WT vs. +α-syn^{AAA}	0.03763	-0.02677 to 0.1020	No	ns	0.2792
Row 66					
WT vs. +α-syn^{WT}	0.007326	-0.04790 to 0.06256	No	ns	0.9232
WT vs. +α-syn^{AAA}	0.03304	-0.02083 to 0.08690	No	ns	0.2493
Row 67					
WT vs. +α-syn^{WT}	0.001181	-0.05608 to 0.05844	No	ns	0.9981
WT vs. +α-syn^{AAA}	0.01934	-0.02236 to 0.06105	No	ns	0.4028
Row 68					
WT vs. +α-syn^{WT}	-0.004627	-0.06064 to 0.05139	No	ns	0.9696
WT vs. +α-syn^{AAA}	0.01482	-0.03382 to 0.06346	No	ns	0.6673
Row 69					
WT vs. +α-syn^{WT}	-0.001966	-0.05770 to 0.05377	No	ns	0.9943
WT vs. +α-syn^{AAA}	0.02578	-0.02403 to 0.07560	No	ns	0.3384
Row 70					
WT vs. +α-syn^{WT}	-0.002594	-0.05730 to 0.05211	No	ns	0.9898
WT vs. +α-syn^{AAA}	0.02244	-0.02880 to 0.07368	No	ns	0.4618
Row 71					
WT vs. +α-syn^{WT}	0.009398	-0.04516 to 0.06396	No	ns	0.8767
WT vs. +α-syn^{AAA}	0.01794	-0.03124 to 0.06713	No	ns	0.5719
Row 72					
WT vs. +α-syn^{WT}	-0.00676	-0.06282 to 0.04930	No	ns	0.9364
WT vs. +α-syn^{AAA}	0.0353	-0.01852 to 0.08912	No	ns	0.2128
Row 73					
WT vs. +α-syn^{WT}	-0.002607	-0.05636 to 0.05114	No	ns	0.9891
WT vs. +α-syn^{AAA}	0.0225	-0.03240 to 0.07739	No	ns	0.5021
Row 74					
WT vs. +α-syn^{WT}	-0.01147	-0.06569 to 0.04276	No	ns	0.8214
WT vs. +α-syn^{AAA}	0.01423	-0.03713 to 0.06558	No	ns	0.7142
Row 75					
WT vs. +α-syn^{WT}	-0.003903	-0.06172 to 0.05391	No	ns	0.9795
WT vs. +α-syn^{AAA}	0.03124	-0.02310 to 0.08559	No	ns	0.2886
Row 76					
WT vs. +α-syn^{WT}	-0.0008685	-0.05535 to 0.05361	No	ns	0.9988
WT vs. +α-syn^{AAA}	0.01575	-0.03686 to 0.06836	No	ns	0.6671
Row 77					

WT vs. +α-syn^{WT}	-0.01145	-0.06406 to 0.04116	No	ns	0.8119
WT vs. +α-syn^{AAA}	0.03614	-0.01914 to 0.09142	No	ns	0.2158
Row 78					
WT vs. +α-syn^{WT}	-0.002111	-0.05482 to 0.05060	No	ns	0.9927
WT vs. +α-syn^{AAA}	0.01684	-0.02978 to 0.06345	No	ns	0.5683
Row 79					
WT vs. +α-syn^{WT}	-0.002481	-0.05914 to 0.05418	No	ns	0.9912
WT vs. +α-syn^{AAA}	0.03053	-0.02627 to 0.08732	No	ns	0.3299
Row 80					
WT vs. +α-syn^{WT}	-0.008224	-0.06417 to 0.04773	No	ns	0.9035
WT vs. +α-syn^{AAA}	0.007458	-0.05048 to 0.06539	No	ns	0.9256
Row 81					
WT vs. +α-syn^{WT}	0.0005754	-0.05367 to 0.05482	No	ns	0.9995
WT vs. +α-syn^{AAA}	0.04469	-0.01492 to 0.1043	No	ns	0.1469
Row 82					
WT vs. +α-syn^{WT}	0.0004107	-0.04658 to 0.04740	No	ns	0.9996
WT vs. +α-syn^{AAA}	0.03609	-0.01591 to 0.08810	No	ns	0.1841
Row 83					
WT vs. +α-syn^{WT}	-0.01797	-0.07236 to 0.03642	No	ns	0.6256
WT vs. +α-syn^{AAA}	0.03495	-0.02432 to 0.09421	No	ns	0.2747
Row 84					
WT vs. +α-syn^{WT}	-0.01388	-0.06831 to 0.04055	No	ns	0.7554
WT vs. +α-syn^{AAA}	0.01047	-0.04234 to 0.06328	No	ns	0.8384
Row 85					
WT vs. +α-syn^{WT}	0.009445	-0.04142 to 0.06031	No	ns	0.8586
WT vs. +α-syn^{AAA}	0.02916	-0.01875 to 0.07707	No	ns	0.2555
Row 86					
WT vs. +α-syn^{WT}	-0.01095	-0.06521 to 0.04330	No	ns	0.8333
WT vs. +α-syn^{AAA}	0.01611	-0.04242 to 0.07465	No	ns	0.7193
Row 87					
WT vs. +α-syn^{WT}	-0.003789	-0.05771 to 0.05014	No	ns	0.9777
WT vs. +α-syn^{AAA}	0.02133	-0.02926 to 0.07192	No	ns	0.4817
Row 88					
WT vs. +α-syn^{WT}	-0.008482	-0.05764 to 0.04068	No	ns	0.8748
WT vs. +α-syn^{AAA}	0.03181	-0.01751 to 0.08113	No	ns	0.2223
Row 89					
WT vs. +α-syn^{WT}	-0.008473	-0.05299 to 0.03605	No	ns	0.8501

WT vs. +α-syn^{AAA}	0.016	-0.03860 to 0.07060	No	ns	0.6885
Row 90					
WT vs. +α-syn^{WT}	0.009325	-0.04094 to 0.05959	No	ns	0.8567
WT vs. +α-syn^{AAA}	0.03012	-0.02741 to 0.08765	No	ns	0.3483
Row 91					
WT vs. +α-syn^{WT}	-0.002593	-0.05651 to 0.05132	No	ns	0.9895
WT vs. +α-syn^{AAA}	0.02515	-0.03097 to 0.08127	No	ns	0.4472
Row 92					
WT vs. +α-syn^{WT}	-0.001383	-0.04938 to 0.04661	No	ns	0.9962
WT vs. +α-syn^{AAA}	0.02702	-0.02761 to 0.08166	No	ns	0.3843
Row 93					
WT vs. +α-syn^{WT}	-0.006112	-0.05385 to 0.04162	No	ns	0.9288
WT vs. +α-syn^{AAA}	0.05522	-0.01601 to 0.1265	No	ns	0.126
Row 94					
WT vs. +α-syn^{WT}	-0.01253	-0.06604 to 0.04099	No	ns	0.7862
WT vs. +α-syn^{AAA}	0.03043	-0.02844 to 0.08931	No	ns	0.3567
Row 95					
WT vs. +α-syn^{WT}	-0.0134	-0.06574 to 0.03893	No	ns	0.7538
WT vs. +α-syn^{AAA}	0.01789	-0.03474 to 0.07052	No	ns	0.614
Row 96					
WT vs. +α-syn^{WT}	-0.0002554	-0.05558 to 0.05507	No	ns	>0.9999
WT vs. +α-syn^{AAA}	0.0363	-0.01841 to 0.09101	No	ns	0.2069
Row 97					
WT vs. +α-syn^{WT}	0.004675	-0.05410 to 0.06345	No	ns	0.9714
WT vs. +α-syn^{AAA}	0.03251	-0.02628 to 0.09129	No	ns	0.3127
Row 98					
WT vs. +α-syn^{WT}	-0.0005206	-0.05353 to 0.05248	No	ns	0.9996
WT vs. +α-syn^{AAA}	0.02891	-0.03088 to 0.08870	No	ns	0.3988
Row 99					
WT vs. +α-syn^{WT}	-0.001183	-0.05074 to 0.04837	No	ns	0.9974
WT vs. +α-syn^{AAA}	0.02041	-0.03835 to 0.07917	No	ns	0.5997
Row 100					
WT vs. +α-syn^{WT}	0.006491	-0.05080 to 0.06378	No	ns	0.9438
WT vs. +α-syn^{AAA}	0.02557	-0.02986 to 0.08100	No	ns	0.4292
Row 101					
WT vs. +α-syn^{WT}	-0.001672	-0.05580 to 0.05246	No	ns	0.9957
WT vs. +α-syn^{AAA}	0.03681	-0.02407 to 0.09768	No	ns	0.2574

**FIGURE 6B**								
Test details	Mean 1	Mean 2	Mean Diff.	SE of diff.	N1	N2	q	DF
Row 1								
WT vs. +α-syn^{WT}	0.0168	0.01624	0.0005594	0.01187	7	8	0.04712	11.45
WT vs. +α-syn^{AAA}	0.0168	0.0342	-0.01739	0.01599	7	7	1.088	11.08
Row 2								
WT vs. +α-syn^{WT}	0.01357	0.006705	0.006868	0.007451	7	8	0.9218	9.497
WT vs. +α-syn^{AAA}	0.01357	0.02775	-0.01418	0.01067	7	7	1.329	11.27
Row 3								
WT vs. +α-syn^{WT}	0.0001593	0.0127	-0.01254	0.007489	7	8	1.674	6.824
WT vs. +α-syn^{AAA}	0.0001593	0.03508	-0.03492	0.01218	7	7	2.866	11.05
Row 4								
WT vs. +α-syn^{WT}	0.004937	0.0006791	0.004258	0.00748	7	8	0.5693	12.73
WT vs. +α-syn^{AAA}	0.004937	0.03524	-0.0303	0.0108	7	7	2.806	8.615
Row 5								
WT vs. +α-syn^{WT}	0.003859	0.004613	-0.0007538	0.005327	7	8	0.1415	12.92
WT vs. +α-syn^{AAA}	0.003859	0.007625	-0.003766	0.009766	7	7	0.3856	8.034
Row 6								
WT vs. +α-syn^{WT}	0.005143	-0.002658	0.007801	0.008058	7	8	0.9681	11.53
WT vs. +α-syn^{AAA}	0.005143	0.001814	0.003329	0.009646	7	7	0.3451	8.792
Row 7								
WT vs. +α-syn^{WT}	0.005674	0.001793	0.003881	0.007023	7	8	0.5526	11.23
WT vs. +α-syn^{AAA}	0.005674	0.0003144	0.005359	0.006203	7	7	0.864	10.86
Row 8								
WT vs. +α-syn^{WT}	-0.00204	0.001491	-0.003531	0.004774	7	8	0.7397	11.51
WT vs. +α-syn^{AAA}	-0.00204	-0.01111	0.009073	0.01045	7	7	0.8685	6.748
Row 9								
WT vs. +α-syn^{WT}	-0.002235	-0.004803	0.002567	0.008757	7	8	0.2931	12.44
WT vs. +α-syn^{AAA}	-0.002235	0.00077	-0.003005	0.008973	7	7	0.3349	10.87
Row 10								
WT vs. +α-syn^{WT}	-0.006541	0.004177	-0.01072	0.005813	7	8	1.844	10.38
WT vs. +α-syn^{AAA}	-0.006541	0.008215	-0.01476	0.008189	7	7	1.802	7.407
Row 11								
WT vs. +α-syn^{WT}	0.01054	0.007612	0.002923	0.01122	7	8	0.2605	12.93
WT vs. +α-syn^{AAA}	0.01054	0.01027	0.0002667	0.01045	7	7	0.02551	11.75
Row 12								

WT vs. +α-syn^{WT}	0.06524	0.0438	0.02143	0.01994	7	8	1.075	9.572
WT vs. +α-syn^{AAA}	0.06524	0.04218	0.02306	0.02076	7	7	1.111	10.33
Row 13								
WT vs. +α-syn^{WT}	0.09771	0.06048	0.03723	0.02982	7	8	1.249	10.33
WT vs. +α-syn^{AAA}	0.09771	0.0839	0.01381	0.03118	7	7	0.443	10.96
Row 14								
WT vs. +α-syn^{WT}	0.1262	0.09057	0.0356	0.03236	7	8	1.1	9.886
WT vs. +α-syn^{AAA}	0.1262	0.1266	-0.000402	0.03117	7	7	0.0129	8.842
Row 15								
WT vs. +α-syn^{WT}	0.1629	0.1004	0.0625	0.03846	7	8	1.625	9.998
WT vs. +α-syn^{AAA}	0.1629	0.1699	-0.006967	0.03921	7	7	0.1777	10.24
Row 16								
WT vs. +α-syn^{WT}	0.1879	0.1207	0.06723	0.04386	7	8	1.533	10.76
WT vs. +α-syn^{AAA}	0.1879	0.1801	0.007744	0.04182	7	7	0.1852	9.482
Row 17								
WT vs. +α-syn^{WT}	0.1984	0.1372	0.06122	0.0435	7	8	1.407	10.89
WT vs. +α-syn^{AAA}	0.1984	0.1996	-0.001197	0.04143	7	7	0.02888	9.596
Row 18								
WT vs. +α-syn^{WT}	0.2137	0.1519	0.06182	0.03718	7	8	1.663	10.74
WT vs. +α-syn^{AAA}	0.2137	0.213	0.0007016	0.03395	7	7	0.02067	8.395
Row 19								
WT vs. +α-syn^{WT}	0.218	0.1711	0.04691	0.04088	7	8	1.147	9.441
WT vs. +α-syn^{AAA}	0.218	0.2224	-0.004336	0.03859	7	7	0.1123	7.881
Row 20								
WT vs. +α-syn^{WT}	0.2435	0.164	0.07944	0.0375	7	8	2.119	10.94
WT vs. +α-syn^{AAA}	0.2435	0.2222	0.02134	0.0348	7	7	0.6131	9.024
Row 21								
WT vs. +α-syn^{WT}	0.2529	0.1718	0.08111	0.03619	7	8	2.241	9.653
WT vs. +α-syn^{AAA}	0.2529	0.246	0.006942	0.03729	7	7	0.1862	10.18
Row 22								
WT vs. +α-syn^{WT}	0.254	0.1763	0.07768	0.03401	7	8	2.284	10.71
WT vs. +α-syn^{AAA}	0.254	0.2482	0.005809	0.03058	7	7	0.1899	7.96
Row 23								
WT vs. +α-syn^{WT}	0.253	0.182	0.07108	0.02975	7	8	2.389	10.08
WT vs. +α-syn^{AAA}	0.253	0.2372	0.01587	0.02807	7	7	0.5654	8.514
Row 24								
WT vs. +α-syn^{WT}	0.2475	0.174	0.07354	0.03117	7	8	2.359	12.16

WT vs. +α-syn^{AAA}	0.2475	0.2466	0.0009556	0.02755	7	7	0.03468	9.467
Row 25								
WT vs. +α-syn^{WT}	0.258	0.1861	0.07187	0.02989	7	8	2.404	12.52
WT vs. +α-syn^{AAA}	0.258	0.2537	0.004344	0.02648	7	7	0.1641	10.16
Row 26								
WT vs. +α-syn^{WT}	0.2476	0.1779	0.06966	0.02802	7	8	2.486	13
WT vs. +α-syn^{AAA}	0.2476	0.253	-0.005447	0.02952	7	7	0.1845	11.62
Row 27								
WT vs. +α-syn^{WT}	0.2337	0.1705	0.06315	0.02564	7	8	2.463	12.79
WT vs. +α-syn^{AAA}	0.2337	0.2192	0.01443	0.02316	7	7	0.623	11.08
Row 28								
WT vs. +α-syn^{WT}	0.2167	0.1625	0.0542	0.02481	7	8	2.185	12.99
WT vs. +α-syn^{AAA}	0.2167	0.2066	0.01017	0.01919	7	7	0.5298	9.675
Row 29								
WT vs. +α-syn^{WT}	0.2113	0.148	0.06327	0.02491	7	8	2.54	12.94
WT vs. +α-syn^{AAA}	0.2113	0.1973	0.01397	0.0191	7	7	0.7315	8.14
Row 30								
WT vs. +α-syn^{WT}	0.1887	0.1366	0.0521	0.02599	7	8	2.005	12.79
WT vs. +α-syn^{AAA}	0.1887	0.1839	0.00476	0.02086	7	7	0.2282	11.38
Row 31								
WT vs. +α-syn^{WT}	0.172	0.137	0.03501	0.02079	7	8	1.684	13
WT vs. +α-syn^{AAA}	0.172	0.1612	0.01075	0.01634	7	7	0.6579	9.679
Row 32								
WT vs. +α-syn^{WT}	0.1713	0.1193	0.05202	0.02283	7	8	2.278	10.62
WT vs. +α-syn^{AAA}	0.1713	0.1459	0.02543	0.01345	7	7	1.891	11.02
Row 33								
WT vs. +α-syn^{WT}	0.1668	0.1275	0.03935	0.01863	7	8	2.112	12.42
WT vs. +α-syn^{AAA}	0.1668	0.1583	0.008557	0.01469	7	7	0.5823	11.78
Row 34								
WT vs. +α-syn^{WT}	0.1603	0.1268	0.03354	0.02177	7	8	1.54	12.31
WT vs. +α-syn^{AAA}	0.1603	0.1342	0.02615	0.01373	7	7	1.904	7.853
Row 35								
WT vs. +α-syn^{WT}	0.1528	0.1144	0.03841	0.02228	7	8	1.724	10.43
WT vs. +α-syn^{AAA}	0.1528	0.1287	0.0241	0.01226	7	7	1.966	10.25
Row 36								
WT vs. +α-syn^{WT}	0.146	0.1076	0.03834	0.01791	7	8	2.141	12.82
WT vs. +α-syn^{AAA}	0.146	0.1273	0.01869	0.01435	7	7	1.303	11.28

Row 37								
WT vs. +α-syn^{WT}	0.1416	0.1042	0.03737	0.02254	7	8	1.658	12.62
WT vs. +α-syn^{AAA}	0.1416	0.1209	0.02066	0.01648	7	7	1.254	10.32
Row 38								
WT vs. +α-syn^{WT}	0.1224	0.09482	0.0276	0.02458	7	8	1.122	12.75
WT vs. +α-syn^{AAA}	0.1224	0.1088	0.01363	0.0183	7	7	0.745	10.25
Row 39								
WT vs. +α-syn^{WT}	0.1225	0.1033	0.01919	0.02509	7	8	0.7649	12.57
WT vs. +α-syn^{AAA}	0.1225	0.1162	0.006311	0.01737	7	7	0.3634	9.266
Row 40								
WT vs. +α-syn^{WT}	0.1176	0.09127	0.02633	0.02177	7	8	1.21	12.99
WT vs. +α-syn^{AAA}	0.1176	0.0993	0.01829	0.01649	7	7	1.109	9.041
Row 41								
WT vs. +α-syn^{WT}	0.1093	0.09576	0.01357	0.02301	7	8	0.5899	12.96
WT vs. +α-syn^{AAA}	0.1093	0.1032	0.00609	0.01828	7	7	0.3331	10.53
Row 42								
WT vs. +α-syn^{WT}	0.1065	0.08566	0.02082	0.02323	7	8	0.8964	12.77
WT vs. +α-syn^{AAA}	0.1065	0.08742	0.01907	0.01667	7	7	1.144	9.357
Row 43								
WT vs. +α-syn^{WT}	0.1097	0.07765	0.03202	0.02589	7	8	1.237	12.98
WT vs. +α-syn^{AAA}	0.1097	0.08522	0.02445	0.01937	7	7	1.263	8.964
Row 44								
WT vs. +α-syn^{WT}	0.09842	0.07611	0.02231	0.02396	7	8	0.9314	13
WT vs. +α-syn^{AAA}	0.09842	0.08139	0.01703	0.01939	7	7	0.8785	10.45
Row 45								
WT vs. +α-syn^{WT}	0.09237	0.08079	0.01158	0.0252	7	8	0.4596	12.93
WT vs. +α-syn^{AAA}	0.09237	0.07007	0.02229	0.02006	7	7	1.112	10.75
Row 46								
WT vs. +α-syn^{WT}	0.08846	0.07446	0.014	0.02156	7	8	0.6495	12.92
WT vs. +α-syn^{AAA}	0.08846	0.0664	0.02206	0.01856	7	7	1.188	11.77
Row 47								
WT vs. +α-syn^{WT}	0.0785	0.06843	0.01008	0.02414	7	8	0.4174	12.98
WT vs. +α-syn^{AAA}	0.0785	0.07051	0.007992	0.01808	7	7	0.442	9.074
Row 48								
WT vs. +α-syn^{WT}	0.0733	0.0698	0.003502	0.02385	7	8	0.1468	13
WT vs. +α-syn^{AAA}	0.0733	0.06468	0.008624	0.01885	7	7	0.4574	9.891

Row 49								
WT vs. +α-syn^{WT}	0.07528	0.0576	0.01768	0.02418	7	8	0.7311	13
WT vs. +α-syn^{AAA}	0.07528	0.04696	0.02833	0.01924	7	7	1.472	10.01
Row 50								
WT vs. +α-syn^{WT}	0.07267	0.06483	0.007842	0.02134	7	8	0.3675	12.54
WT vs. +α-syn^{AAA}	0.07267	0.05521	0.01747	0.01803	7	7	0.9686	9.148
Row 51								
WT vs. +α-syn^{WT}	0.06746	0.05532	0.01214	0.02582	7	8	0.4703	12.86
WT vs. +α-syn^{AAA}	0.06746	0.05242	0.01504	0.01806	7	7	0.8327	8.276
Row 52								
WT vs. +α-syn^{WT}	0.06888	0.05871	0.01017	0.02386	7	8	0.4261	12.02
WT vs. +α-syn^{AAA}	0.06888	0.04715	0.02173	0.02119	7	7	1.026	9.36
Row 53								
WT vs. +α-syn^{WT}	0.06273	0.06187	0.0008605	0.02463	7	8	0.03493	12.99
WT vs. +α-syn^{AAA}	0.06273	0.04153	0.0212	0.0189	7	7	1.122	8.783
Row 54								
WT vs. +α-syn^{WT}	0.05023	0.04844	0.001786	0.02253	7	8	0.07925	12.94
WT vs. +α-syn^{AAA}	0.05023	0.04691	0.003323	0.01895	7	7	0.1753	11.51
Row 55								
WT vs. +α-syn^{WT}	0.05546	0.04569	0.009773	0.02606	7	8	0.375	13
WT vs. +α-syn^{AAA}	0.05546	0.03898	0.01648	0.01979	7	7	0.8328	8.592
Row 56								
WT vs. +α-syn^{WT}	0.04147	0.04938	-0.007911	0.02234	7	8	0.3542	11.92
WT vs. +α-syn^{AAA}	0.04147	0.02928	0.01219	0.02031	7	7	0.5999	9.752
Row 57								
WT vs. +α-syn^{WT}	0.05249	0.05017	0.002316	0.02356	7	8	0.09833	12.79
WT vs. +α-syn^{AAA}	0.05249	0.02755	0.02494	0.02071	7	7	1.204	10.62
Row 58								
WT vs. +α-syn^{WT}	0.04933	0.05377	-0.004441	0.02134	7	8	0.2081	12.73
WT vs. +α-syn^{AAA}	0.04933	0.02321	0.02612	0.01884	7	7	1.387	10.54
Row 59								
WT vs. +α-syn^{WT}	0.04722	0.04365	0.003572	0.02098	7	8	0.1703	12.51
WT vs. +α-syn^{AAA}	0.04722	0.02438	0.02285	0.01964	7	7	1.163	11.13
Row 60								
WT vs. +α-syn^{WT}	0.04158	0.04351	-0.001933	0.01987	7	8	0.09731	12.94
WT vs. +α-syn^{AAA}	0.04158	0.03166	0.009918	0.01686	7	7	0.5881	10.51
Row 61								

WT vs. +α-syn^{WT}	0.04526	0.03357	0.01169	0.02581	7	8	0.4529	12.54
WT vs. +α-syn^{AAA}	0.04526	0.0237	0.02157	0.02022	7	7	1.066	7.223
Row 62								
WT vs. +α-syn^{WT}	0.03022	0.04206	-0.01184	0.02431	7	8	0.4868	12.96
WT vs. +α-syn^{AAA}	0.03022	0.02554	0.004681	0.02007	7	7	0.2333	11.16
Row 63								
WT vs. +α-syn^{WT}	0.03433	0.03649	-0.002157	0.02196	7	8	0.09823	12.28
WT vs. +α-syn^{AAA}	0.03433	0.01571	0.01862	0.02093	7	7	0.8898	11.11
Row 64								
WT vs. +α-syn^{WT}	0.0212	0.03374	-0.01254	0.02067	7	8	0.6066	12.96
WT vs. +α-syn^{AAA}	0.0212	0.002629	0.01857	0.01827	7	7	1.016	11.27
Row 65								
WT vs. +α-syn^{WT}	0.02995	0.02714	0.002804	0.02226	7	8	0.126	12.65
WT vs. +α-syn^{AAA}	0.02995	-0.007687	0.03763	0.02566	7	7	1.467	11.62
Row 66								
WT vs. +α-syn^{WT}	0.03525	0.02792	0.007326	0.02216	7	8	0.3306	12.27
WT vs. +α-syn^{AAA}	0.03525	0.002211	0.03304	0.02137	7	7	1.546	11.28
Row 67								
WT vs. +α-syn^{WT}	0.02738	0.0262	0.001181	0.02307	7	8	0.05121	12.7
WT vs. +α-syn^{AAA}	0.02738	0.008038	0.01934	0.01583	7	7	1.222	8.572
Row 68								
WT vs. +α-syn^{WT}	0.02407	0.0287	-0.004627	0.02263	7	8	0.2045	13
WT vs. +α-syn^{AAA}	0.02407	0.009248	0.01482	0.01927	7	7	0.7691	11.18
Row 69								
WT vs. +α-syn^{WT}	0.0232	0.02517	-0.001966	0.02238	7	8	0.08785	12.38
WT vs. +α-syn^{AAA}	0.0232	-0.002582	0.02578	0.01908	7	7	1.351	9
Row 70								
WT vs. +α-syn^{WT}	0.02952	0.03211	-0.002594	0.0221	7	8	0.1174	12.99
WT vs. +α-syn^{AAA}	0.02952	0.007077	0.02244	0.02047	7	7	1.097	11.86
Row 71								
WT vs. +α-syn^{WT}	0.02194	0.01255	0.009398	0.02204	7	8	0.4263	13
WT vs. +α-syn^{AAA}	0.02194	0.004	0.01794	0.01959	7	7	0.9161	11.59
Row 72								
WT vs. +α-syn^{WT}	0.02196	0.02872	-0.00676	0.02259	7	8	0.2993	12.7
WT vs. +α-syn^{AAA}	0.02196	-0.01334	0.0353	0.02143	7	7	1.647	11.56
Row 73								
WT vs. +α-syn^{WT}	0.01624	0.01884	-0.002607	0.02147	7	8	0.1214	11.88

WT vs. +α-syn^{AAA}	0.01624	-0.006259	0.0225	0.02186	7	7	1.029	11.6
Row 74								
WT vs. +α-syn^{WT}	0.01075	0.02222	-0.01147	0.02186	7	8	0.5246	12.75
WT vs. +α-syn^{AAA}	0.01075	-0.003474	0.01423	0.02042	7	7	0.6966	11.46
Row 75								
WT vs. +α-syn^{WT}	0.01418	0.01809	-0.003903	0.02333	7	8	0.1673	12.85
WT vs. +α-syn^{AAA}	0.01418	-0.01706	0.03124	0.02163	7	7	1.445	11.54
Row 76								
WT vs. +α-syn^{WT}	0.009371	0.01024	-0.0008685	0.02158	7	8	0.04024	11.16
WT vs. +α-syn^{AAA}	0.009371	-0.00638	0.01575	0.02041	7	7	0.7717	9.715
Row 77								
WT vs. +α-syn^{WT}	0.007637	0.01909	-0.01145	0.02118	7	8	0.5407	12.6
WT vs. +α-syn^{AAA}	0.007637	-0.0285	0.03614	0.02211	7	7	1.634	12
Row 78								
WT vs. +α-syn^{WT}	0.008263	0.01037	-0.002111	0.02125	7	8	0.09931	12.78
WT vs. +α-syn^{AAA}	0.008263	-0.008573	0.01684	0.01819	7	7	0.9256	10.07
Row 79								
WT vs. +α-syn^{WT}	0.006099	0.00858	-0.002481	0.02268	7	8	0.1094	12.07
WT vs. +α-syn^{AAA}	0.006099	-0.02443	0.03053	0.02259	7	7	1.351	11.49
Row 80								
WT vs. +α-syn^{WT}	0.003425	0.01165	-0.008224	0.02201	7	8	0.3736	10.63
WT vs. +α-syn^{AAA}	0.003425	-0.004033	0.007458	0.02294	7	7	0.3252	11.1
Row 81								
WT vs. +α-syn^{WT}	0.007209	0.006634	0.0005754	0.0214	7	8	0.02689	10.82
WT vs. +α-syn^{AAA}	0.007209	-0.03749	0.04469	0.02382	7	7	1.877	11.87
Row 82								
WT vs. +α-syn^{WT}	-0.00008243	-0.0004931	0.0004107	0.0189	7	8	0.02173	12.53
WT vs. +α-syn^{AAA}	-0.00008243	-0.03617	0.03609	0.02079	7	7	1.736	11.93
Row 83								
WT vs. +α-syn^{WT}	-0.0006247	0.01734	-0.01797	0.02157	7	8	0.833	11.24
WT vs. +α-syn^{AAA}	-0.0006247	-0.03557	0.03495	0.02368	7	7	1.476	11.9
Row 84								
WT vs. +α-syn^{WT}	-0.009065	0.004815	-0.01388	0.02198	7	8	0.6314	12.97
WT vs. +α-syn^{AAA}	-0.009065	-0.01954	0.01047	0.02112	7	7	0.4959	11.97
Row 85								
WT vs. +α-syn^{WT}	-0.003422	-0.01287	0.009445	0.02054	7	8	0.4599	12.93
WT vs. +α-syn^{AAA}	-0.003422	-0.03258	0.02916	0.01911	7	7	1.526	11.73

Row 86								
WT vs. +α-syn^{WT}	-0.00456	0.006393	-0.01095	0.0217	7	8	0.5048	11.97
WT vs. +α-syn^{AAA}	-0.00456	-0.02067	0.01611	0.02341	7	7	0.6883	11.98
Row 87								
WT vs. +α-syn^{WT}	-0.007036	-0.003247	-0.003789	0.02171	7	8	0.1745	12.65
WT vs. +α-syn^{AAA}	-0.007036	-0.02837	0.02133	0.02003	7	7	1.065	11.12
Row 88								
WT vs. +α-syn^{WT}	-0.004057	0.004425	-0.008482	0.01973	7	8	0.43	12.28
WT vs. +α-syn^{AAA}	-0.004057	-0.03587	0.03181	0.01966	7	7	1.618	11.66
Row 89								
WT vs. +α-syn^{WT}	-0.007129	0.001343	-0.008473	0.01782	7	8	0.4754	12.06
WT vs. +α-syn^{AAA}	-0.007129	-0.02313	0.016	0.02174	7	7	0.736	11.57
Row 90								
WT vs. +α-syn^{WT}	-0.003758	-0.01308	0.009325	0.02012	7	8	0.4636	12.03
WT vs. +α-syn^{AAA}	-0.003758	-0.03388	0.03012	0.023	7	7	1.31	11.93
Row 91								
WT vs. +α-syn^{WT}	-0.01014	-0.007545	-0.002593	0.02178	7	8	0.119	13
WT vs. +α-syn^{AAA}	-0.01014	-0.03529	0.02515	0.02241	7	7	1.122	11.84
Row 92								
WT vs. +α-syn^{WT}	-0.01509	-0.01371	-0.001383	0.01928	7	8	0.07175	12.37
WT vs. +α-syn^{AAA}	-0.01509	-0.04211	0.02702	0.02183	7	7	1.238	11.87
Row 93								
WT vs. +α-syn^{WT}	-0.01037	-0.004261	-0.006112	0.01925	7	8	0.3176	12.78
WT vs. +α-syn^{AAA}	-0.01037	-0.0656	0.05522	0.02717	7	7	2.032	8.802
Row 94								
WT vs. +α-syn^{WT}	-0.02152	-0.008999	-0.01253	0.02149	7	8	0.5827	12.39
WT vs. +α-syn^{AAA}	-0.02152	-0.05196	0.03043	0.02355	7	7	1.292	11.97
Row 95								
WT vs. +α-syn^{WT}	-0.02111	-0.007704	-0.0134	0.02114	7	8	0.634	12.99
WT vs. +α-syn^{AAA}	-0.02111	-0.03899	0.01789	0.02105	7	7	0.8498	11.98
Row 96								
WT vs. +α-syn^{WT}	-0.01543	-0.01518	-0.0002554	0.02225	7	8	0.01148	12.5
WT vs. +α-syn^{AAA}	-0.01543	-0.05173	0.0363	0.02181	7	7	1.665	11.67
Row 97								
WT vs. +α-syn^{WT}	-0.01319	-0.01786	0.004675	0.02358	7	8	0.1983	12.27
WT vs. +α-syn^{AAA}	-0.01319	-0.04569	0.03251	0.02342	7	7	1.388	11.61

Row 98								
WT vs. +α-syn^{WT}	-0.01668	-0.01616	-0.0005206	0.0213	7	8	0.02444	12.44
WT vs. +α-syn^{AAA}	-0.01668	-0.0456	0.02891	0.02389	7	7	1.21	11.89
Row 99								
WT vs. +α-syn^{WT}	-0.0194	-0.01822	-0.001183	0.01994	7	8	0.05933	12.6
WT vs. +α-syn^{AAA}	-0.0194	-0.03982	0.02041	0.02339	7	7	0.8727	11.54
Row 100								
WT vs. +α-syn^{WT}	-0.02209	-0.02858	0.006491	0.02313	7	8	0.2806	12.94
WT vs. +α-syn^{AAA}	-0.02209	-0.04766	0.02557	0.02216	7	7	1.154	11.95
Row 101								
WT vs. +α-syn^{WT}	-0.02565	-0.02398	-0.001672	0.02187	7	8	0.07648	12.99
WT vs. +α-syn^{AAA}	-0.02565	-0.06246						

**FIGURE 6C**	WT	+α-syn^{WT}	+α-syn^{AAA}
Number of values	7	8	7
Mean	0.2747	0.2045	0.2714
Std. Deviation	0.07189	0.04264	0.04609
Std. Error of Mean	0.02717	0.01508	0.01742
	WT	+α-syn^{WT}	+α-syn^{AAA}
Test for normal distribution			
Shapiro-Wilk test			
W	0.9296	0.9666	0.9161
P value	0.5471	0.8701	0.4395
Passed normality test (alpha=0.05)?	Yes	Yes	Yes
P value summary	ns	ns	ns

**FIGURE 6C**	
ANOVA summary	
F	4.028
P value	0.0348
P value summary	*
Significant diff. among means (P < 0.05)?	Yes
R squared	0.2977
Brown-Forsythe test	
F (DFn, DFd)	1.527 (2, 19)
P value	0.2426
P value summary	ns
Are SDs significantly different (P < 0.05)?	No
Bartlett's test	
Bartlett's statistic (corrected)	1.999
P value	0.3681
P value summary	ns
Are SDs significantly different (P < 0.05)?	No

**FIGURE 6C**					
ANOVA table	SS	DF	MS	F (DFn, DFd)	P value
Treatment (between columns)	0.02395	2	0.01197	F (2, 19) = 4.028	P=0.0348
Residual (within columns)	0.05648	19	0.002973		
Total	0.08043	21			
Data summary					
Number of treatments (columns)	3				
Number of values (total)	22				
Number of families	1				
Number of comparisons per family	2				
Alpha	0.05				

**FIGURE 6C**								
Dunnett's multiple comparisons test	Mean Diff.	95.00% CI of diff.	Below threshold?	Summary	Adjusted P Value	A-?		
WT vs. +α-syn^{WT}	0.07018	0.002858 to 0.1375	Yes	*	0.0407	B	+α-syn^{WT}	
WT vs. +α-syn^{AAA}	0.003301	-0.06623 to 0.07283	No	ns	0.9905	C	+α-syn^{AAA}	
Test details	Mean 1	Mean 2	Mean Diff.	SE of diff.	n1	n2	q	DF
WT vs. +α-syn^{WT}	0.2747	0.2045	0.07018	0.02822	7	8	2.487	19
WT vs. +α-syn^{AAA}	0.2747	0.2714	0.003301	0.02914	7	7	0.1133	19

**FIGURE 6D**	WT	+α-syn^{WT}	+α-syn^{AAA}
Number of values	7	8	7
Mean	0.01726	0.01093	0.01822
Std. Deviation	0.007643	0.005815	0.005261
Std. Error of Mean	0.002889	0.002056	0.001988
	WT	+α-syn^{WT}	+α-syn^{AAA}
Test for normal distribution			
Shapiro-Wilk test			
W	0.9387	0.9542	0.931
P value	0.6267	0.7535	0.5592
Passed normality test (alpha=0.05)?	Yes	Yes	Yes
P value summary	ns	ns	ns

**FIGURE 6D**	
ANOVA summary	
F	3.017
P value	0.0728
P value summary	ns
Significant diff. among means (P < 0.05)?	No
R squared	0.241
Brown-Forsythe test	
F (DFn, DFd)	0.5057 (2, 19)
P value	0.611
P value summary	ns
Are SDs significantly different (P < 0.05)?	No
Bartlett's test	
Bartlett's statistic (corrected)	0.8821
P value	0.6433
P value summary	ns
Are SDs significantly different (P < 0.05)?	No

**FIGURE 6D**					
ANOVA table	SS	DF	MS	F (DFn, DFd)	P value
Treatment (between columns)	0.0002392	2	0.0001196	F (2, 19) = 3.017	P=0.0728
Residual (within columns)	0.0007532	19	0.00003964		
Total	0.0009924	21			
Data summary					
Number of treatments (columns)	3				
Number of values (total)	22				
Number of families	1				
Number of comparisons per family	2				
Alpha	0.05				

**FIGURE 6D**								
Dunnett's multiple comparisons test	Mean Diff.	95.00% CI of diff.	Below threshold?	Summary	Adjusted P Value	A-?		
WT vs. +α-syn^{WT}	0.00633	-0.001444 to 0.01410	No	ns	0.1177	B	+α-syn^{WT}	
WT vs. +α-syn^{AAA}	-0.000956	-0.008985 to 0.007073	No	ns	0.9424	C	+α-syn^{AAA}	
Test details	Mean 1	Mean 2	Mean Diff.	SE of diff.	n1	n2	q	DF
WT vs. +α-syn^{WT}	0.01726	0.01093	0.00633	0.003259	7	8	1.943	19
WT vs. +α-syn^{AAA}	0.01726	0.01822	-0.000956	0.003366	7	7	0.2841	19

**SUPP FIG 1A**	EV double	EV triple	α-syn double	α-syn triple
Number of values	7	8	8	7
Mean	0.9872	0.9803	2.079	1.943
Std. Deviation	0.06794	0.05306	0.2145	0.2187
Std. Error of Mean	0.02568	0.01876	0.07584	0.08267
	EV double	EV triple	α-syn double	α-syn triple
Test for normal distribution				
Shapiro-Wilk test				
W	0.8841	0.9651	0.9831	0.9683
P value	0.2452	0.8568	0.9766	0.8861
Passed normality test (alpha=0.05)?	Yes	Yes	Yes	Yes
P value summary	ns	ns	ns	ns

**SUPP FIG1A**	
ANOVA summary	
F	106.3
P value	<0.0001
P value summary	****
Significant diff. among means (P < 0.05)?	Yes
R squared	0.9246
Brown-Forsythe test	
F (DFn, DFd)	4.753 (3, 26)
P value	0.009
P value summary	**
Are SDs significantly different (P < 0.05)?	Yes
Bartlett's test	
Bartlett's statistic (corrected)	16.46
P value	0.0009
P value summary	***
Are SDs significantly different (P < 0.05)?	Yes

**SUPP FIG 1A**					
ANOVA table	SS	DF	MS	F (DFn, DFd)	P value
Treatment (between columns)	8.053	3	2.684	F (3, 26) = 106.3	P<0.0001
Residual (within columns)	0.6565	26	0.02525		
Total	8.709	29			
Data summary					
Number of treatments (columns)	4				
Number of values (total)	30				
Number of families	1				
Number of comparisons per family	4				
Alpha	0.05				

**SUPP FIG 1A**								
Šídák's multiple comparisons test	Mean Diff.	95.00% CI of diff.	Below threshold?	Summary	Adjusted P Value			
EV double vs. α-syn double	-1.091	-1.311 to -0.8714	Yes	****	<0.0001	A-C		
EV triple vs. α-syn triple	-0.9625	-1.183 to -0.7425	Yes	****	<0.0001	B-D		
EV double vs. EV triple	0.00689	-0.2131 to 0.2269	No	ns	>0.9999	A-B		
α-syn double vs. α-syn triple	0.1358	-0.08419 to 0.3559	No	ns	0.3743	C-D		
Test details	Mean 1	Mean 2	Mean Diff.	SE of diff.	n1	n2	t	DF
EV double vs. α-syn double	0.9872	2.079	-1.091	0.08224	7	8	13.27	26
EV triple vs. α-syn triple	0.9803	1.943	-0.9625	0.08224	8	7	11.7	26
EV double vs. EV triple	0.9872	0.9803	0.00689	0.08224	7	8	0.08378	26
α-syn double vs. α-syn triple	2.079	1.943	0.1358	0.08224	8	7	1.652	26

**SUPP FIG 1B**	EV double	a-syn double
Number of values	7	8
Mean	1.281	2.321
Std. Deviation	0.1191	0.775
Std. Error of Mean	0.04501	0.274
	EV double	a-syn double
Shapiro-Wilk test		
W	0.9686	0.8896
P value	0.8879	0.2319
Passed normality test (alpha=0.05)?	Yes	Yes
P value summary	ns	ns

**SUPP FIG 1B**	
Column B	a-syn double
vs.	vs.
Column A	EV double
Unpaired t test with Welch's correction	
P value	0.0066
P value summary	**
Significantly different (P < 0.05)?	Yes
One- or two-tailed P value?	Two-tailed
Welch-corrected t, df	t=3.745, df=7.377
How big is the difference?	
Mean of column A	1.281
Mean of column B	2.321
Difference between means (B - A) ± SEM	1.040 ± 0.2777
95% confidence interval	0.3900 to 1.690
R squared (eta squared)	0.6553
F test to compare variances	
F, DFn, Dfd	42.35, 7, 6
P value	0.0002
P value summary	***
Significantly different (P < 0.05)?	Yes
Data analyzed	
Sample size, column A	7
Sample size, column B	8

**SUPP FIG 1C**	EV triple	a-syn triple
Number of values	8	7
Mean	1.354	2.097
Std. Deviation	0.1285	0.4551
Std. Error of Mean	0.04543	0.172
	EV triple	a-syn triple
Shapiro-Wilk test		
W	0.9539	0.9926
P value	0.7504	0.9971
Passed normality test (alpha=0.05)?	Yes	Yes
P value summary	ns	ns

**SUPP FIG 1C**	
Column B	a-syn triple
vs.	vs.
Column A	EV triple
Unpaired t test with Welch's correction	
P value	0.0044
P value summary	**
Significantly different (P < 0.05)?	Yes
One- or two-tailed P value?	Two-tailed
Welch-corrected t, df	t=4.177, df=6.838
How big is the difference?	
Mean of column A	1.354
Mean of column B	2.097
Difference between means (B - A) ± SEM	0.7431 ± 0.1779
95% confidence interval	0.3203 to 1.166
R squared (eta squared)	0.7184
F test to compare variances	
F, DFn, Dfd	12.54, 6, 7
P value	0.0039
P value summary	**
Significantly different (P < 0.05)?	Yes
Data analyzed	
Sample size, column A	8
Sample size, column B	7

**SUPP FIG 2C**	α-syn^{WT}	α-syn^{AAA}
Number of values	3	3
Mean	0.348469	0.350334
Std. Deviation	0.029659	0.022806
Std. Error of Mean	0.0171	0.0132
Shapiro-Wilk test		
W	0.8445	0.9429
P value	0.2258	0.5393
Passed normality test (alpha=0.05)?	Yes	Yes
P value summary	ns	ns

**SUPP FIG 2C**	
Column B	α-syn^{AAA}
vs.	vs.
Column A	α-syn^{WT}
Paired t test	
P value	0.7276
P value summary	ns
Significantly different (P < 0.05)?	No
One- or two-tailed P value?	Two-tailed
t, df	t=0.4003, df=2
Number of pairs	3
How large is the effect?	
Mean of differences (B - A)	0.001865
SD of differences	0.008071
SEM of differences	0.00466
95% confidence interval	-0.01818 to 0.02191
R squared (partial eta squared)	0.07418
How effective was the pairing?	
Correlation coefficient (r)	0.9866
P value (one tailed)	0.0522
P value summary	ns
Was the pairing significantly effective?	No

**SUPP FIG 2D**	α-syn^{WT}	α-syn^{AAA}
Number of values	3	3
Mean	0.8214	0.9192
Std. Deviation	0.2255	0.3199
Std. Error of Mean	0.1302	0.1847
Shapiro-Wilk test		
W	0.93	0.8501
P value	0.4884	0.2407
Passed normality test (alpha=0.05)?	Yes	Yes
P value summary	ns	ns

**SUPP FIG 2D**	
Column B	α-syn^{AAA}
vs.	vs.
Column A	α-syn^{WT}
Paired t test	
P value	0.6877
P value summary	ns
Significantly different (P < 0.05)?	No
One- or two-tailed P value?	Two-tailed
t, df	t=0.4650, df=2
Number of pairs	3
How large is the effect?	
Mean of differences (B - A)	0.09784
SD of differences	0.3644
SEM of differences	0.2104
95% confidence interval	-0.8075 to 1.003
R squared (partial eta squared)	0.09756
How effective was the pairing?	
Correlation coefficient (r)	0.1414
P value (one tailed)	0.4548
P value summary	ns
Was the pairing significantly effective?	No

**SUPP FIG 2F**	No GFP ab	No FLAG ab	α-syn^{WT}	α-syn^{AAA}
Number of values	3	3	3	3
Mean	802.2769	781.9467	811.4994	782.4533
Std. Deviation	24.3777	39.4269	71.068	61.7831
Std. Error of Mean	14.0745	22.7631	41.0311	35.6705
Shapiro-Wilk test				
W	0.9901	0.9681	0.9699	0.864
P value	0.8099	0.6568	0.667	0.2786
Passed normality test (alpha=0.05)?	Yes	Yes	Yes	Yes
P value summary	ns	ns	ns	ns

**SUPP FIG 2F**								
Number of families	1							
Number of comparisons per family	6							
Alpha	0.05							
Tukey's multiple comparisons test	Mean diff.	95.00% CI of diff.	Below threshold?	Summary	Adjusted P Value			
No GFP ab vs. No FLAG ab	20.33	-116.9 to 157.5	No	ns	0.9626	A-B		
No GFP ab vs. α-syn^{WT}	-9.222	-146.4 to 128.0	No	ns	0.9962	A-C		
No GFP ab vs. α-syn^{AAA}	19.82	-117.4 to 157.0	No	ns	0.9652	A-D		
No FLAG ab vs. α-syn^{WT}	-29.55	-166.8 to 107.7	No	ns	0.8982	B-C		
No FLAG ab vs. α-syn^{AAA}	-0.5066	-137.7 to 136.7	No	ns	>0.9999	B-D		
α-syn^{WT} vs. α-syn^{AAA}	29.05	-108.2 to 166.3	No	ns	0.9026	C-D		
Test details	Mean 1	Mean 2	Mean diff.	SE of diff.	n1	n2	q	DF
No GFP ab vs. No FLAG ab	802.3	781.9	20.33	42.85	3	3	0.671	8
No GFP ab vs. α-syn^{WT}	802.3	811.5	-9.222	42.85	3	3	0.3044	8
No GFP ab vs. α-syn^{AAA}	802.3	782.5	19.82	42.85	3	3	0.6543	8
No FLAG ab vs. α-syn^{WT}	781.9	811.5	-29.55	42.85	3	3	0.9754	8
No FLAG ab vs. α-syn^{AAA}	781.9	782.5	-0.5066	42.85	3	3	0.01672	8
α-syn^{WT} vs. α-syn^{AAA}	811.5	782.5	29.05	42.85	3	3	0.9586	8

**SUPP FIG 2G**	No GFP ab	No FLAG ab	α-syn^{WT}	α-syn^{AAA}
Number of values	3	3	3	3
Mean	0.3768	0.9031	45.2584	16.4089
Std. Deviation	0.1102	0.9984	5.7677	8.7868
Std. Error of Mean	0.0636	0.5764	3.3298	5.073
Shapiro-Wilk test				
W	0.9439	0.8275	0.9005	0.7799
P value	0.5433	0.182	0.3871	0.0673
Passed normality test (alpha=0.05)?	Yes	Yes	Yes	Yes
P value summary	ns	ns	ns	ns

**SUPP FIG 2G**	
Column D	α-syn^{AAA}
vs.	vs.
Column C	α-syn^{WT}
Unpaired t test	
P value	0.0201
P value summary	*
Significantly different (P < 0.05)?	Yes
One- or two-tailed P value?	Two-tailed
t, df	t=3.741, df=4
How large is the effect?	
Mean of column C	45.26
Mean of column D	16.41
Difference between means (D - C) ± SEM	-28.85 ± 7.711
95% confidence interval	-50.26 to -7.440
R squared (eta squared)	0.7777
F test to compare variances	
F, DFn, Dfd	1.310, 2, 2
P value	0.8656
P value summary	ns
Significantly different (P < 0.05)?	No
Data analyzed	
Sample size, column C	3
Sample size, column D	3

**SUPP FIG 4**	WT	+α-syn^{WT}	+α-syn^{AAA}
Number of values	4	4	4
Mean	1.086	2.244	2.446
Std. Deviation	0.1287	0.5299	0.7162
Std. Error of Mean	0.06436	0.265	0.3581
	WT	+α-syn^{WT}	+α-syn^{AAA}
Test for normal distribution			
Shapiro-Wilk test			
W	0.901	0.8522	0.9436
P value	0.4359	0.2334	0.6766
Passed normality test (alpha=0.05)?	Yes	Yes	Yes
P value summary	ns	ns	ns

**SUPP FIG 4**	
ANOVA summary	
F	7.979
P value	0.0102
P value summary	*
Significant diff. among means (P < 0.05)?	Yes
R squared	0.6394
Brown-Forsythe test	
F (DFn, DFd)	1.621 (2, 9)
P value	0.2505
P value summary	ns
Are SDs significantly different (P < 0.05)?	No
Bartlett's test	
Bartlett's statistic (corrected)	5.516
P value	0.0634
P value summary	ns
Are SDs significantly different (P < 0.05)?	No

**SUPP FIG 4**					
ANOVA table	SS	DF	MS	F (DFn, DFd)	P value
Treatment (between columns)	4.31	2	2.155	F (2, 9) = 7.979	P=0.0102
Residual (within columns)	2.431	9	0.2701		
Total	6.742	11			
Data summary					
Number of treatments (columns)	3				
Number of values (total)	12				
Number of families	1				
Number of comparisons per family	3				
Alpha	0.05				

**SUPP FIG 4**								
Tukey's multiple comparisons test	Mean Diff.	95.00% CI of diff.	Below threshold?	Summary	Adjusted P Value			
WT vs. +α-syn^{WT}	-1.158	-2.184 to -0.1323	Yes	*	0.0285	A-B		
WT vs. +α-syn^{AAA}	-1.36	-2.386 to -0.3341	Yes	*	0.0123	A-C		
+α-syn^{WT} vs. +α-syn^{AAA}	-0.2018	-1.228 to 0.8242	No	ns	0.8494	B-C		
Test details	Mean 1	Mean 2	Mean Diff.	SE of diff.	n1	n2	q	DF
WT vs. +α-syn^{WT}	1.086	2.244	-1.158	0.3675	4	4	4.458	9
WT vs. +α-syn^{AAA}	1.086	2.446	-1.36	0.3675	4	4	5.234	9
+α-syn^{WT} vs. +α-syn^{AAA}	2.244	2.446	-0.2018	0.3675	4	4	0.7767	9

**SUPP FIG 5B**	α-syn^{WT}	α-syn^{AAA}
Number of values	6	5
Mean	91.1114	76.7115
Std. Deviation	13.8968	20.1114
Std. Error of Mean	5.6733	8.9941
Shapiro-Wilk test		
W	0.879	0.9299
P value	0.2644	0.596
Passed normality test (alpha=0.05)?	Yes	Yes
P value summary	ns	ns

**SUPP FIG 5B**	
Column B	α-syn^{AAA}
vs.	vs.
Column A	α-syn^{WT}
Unpaired t test	
P value	0.194
P value summary	ns
Significantly different (P < 0.05)?	No
One- or two-tailed P value?	Two-tailed
t, df	t=1.404, df=9
How large is the effect?	
Mean of column A	91.11
Mean of column B	76.71
Difference between means (B - A) ± SEM	-14.40 ± 10.26
95% confidence interval	-37.61 to 8.808
R squared (eta squared)	0.1796
F test to compare variances	
F, DFn, Dfd	2.094, 4, 5
P value	0.4382
P value summary	ns
Significantly different (P < 0.05)?	No
Data analyzed	
Sample size, column A	6
Sample size, column B	5

**SUPP FIG 5C**	α-syn^{WT}	α-syn^{AAA}
Number of values	6	5
Mean	0.62436	0.59005
Std. Deviation	0.09255	0.07794
Std. Error of Mean	0.03778	0.03486
Shapiro-Wilk test		
W	0.8687	0.6773
P value	0.2212	0.0055
Passed normality test (alpha=0.05)?	Yes	No
P value summary	ns	**

**SUPP FIG 5C**	
Column B	α-syn^{AAA}
vs.	vs.
Column A	α-syn^{WT}
Mann Whitney test	
P value	0.9307
Exact or approximate P value?	Exact
P value summary	ns
Significantly different (P < 0.05)?	No
One- or two-tailed P value?	Two-tailed
Sum of ranks in column A,B	37 , 29
Mann-Whitney U	14
Difference between medians	
Median of column A	0.6250, n=6
Median of column B	0.5570, n=5
Difference: Actual	-0.06803
Difference: Hodges-Lehmann	-0.009835

**SUPP FIG 5E**	α-syn^{WT}	α-syn^{AAA}
Number of values	6	5
Mean	-0.20259	-0.11853
Std. Deviation	0.04228	0.05092
Std. Error of Mean	0.01726	0.02277
Shapiro-Wilk test		
W	0.9424	0.8797
P value	0.6782	0.3078
Passed normality test (alpha=0.05)?	Yes	Yes
P value summary	ns	ns

**SUPP FIG 5E**	
Column B	α-syn^{AAA}
vs.	vs.
Column A	α-syn^{WT}
Unpaired t test	
P value	0.015
P value summary	*
Significantly different (P < 0.05)?	Yes
One- or two-tailed P value?	Two-tailed
t, df	t=2.997, df=9
How large is the effect?	
Mean of column A	-0.2026
Mean of column B	-0.1185
Difference between means (B - A) ± SEM	0.08406 ± 0.02805
95% confidence interval	0.02061 to 0.1475
R squared (eta squared)	0.4995
F test to compare variances	
F, DFn, Dfd	1.450, 4, 5
P value	0.6831
P value summary	ns
Significantly different (P < 0.05)?	No
Data analyzed	
Sample size, column A	6
Sample size, column B	5

**SUPP FIG 5G**	α-syn^{WT}	α-syn^{AAA}
Number of values	6	5
Mean	12.8254	-25.5379
Std. Deviation	5.8208	25.9537
Std. Error of Mean	2.3763	11.6069
Shapiro-Wilk test		
W	0.9094	0.7723
P value	0.4324	0.0473
Passed normality test (alpha=0.05)?	Yes	No
P value summary	ns	*

**SUPP FIG 5G**	
Column B	α-syn^{AAA}
vs.	vs.
Column A	α-syn^{WT}
Mann Whitney test	
P value	0.0043
Exact or approximate P value?	Exact
P value summary	**
Significantly different (P < 0.05)?	Yes
One- or two-tailed P value?	Two-tailed
Sum of ranks in column A,B	51 , 15
Mann-Whitney U	0
Difference between medians	
Median of column A	11.37, n=6
Median of column B	-10.34, n=5
Difference: Actual	-21.71
Difference: Hodges-Lehmann	-26.41

**SUPP FIG 7B**					
Two-way RM ANOVA	Matching Stacked				
Assume sphericity?	No				
Alpha	0.05				
Source of Variation	% of total variation	P value	P value summary	Significant?	Geisser-Greenhouse's epsilon
Time x Column Factor	1.905	0.1694	ns	No	0.02644
Time	72.15	<0.0001	****	Yes	0.02644
Column Factor	2.075	0.2628	ns	No	
Subject	13.74	<0.0001	****	Yes	
ANOVA table	SS	DF	MS	F (DFn, DFd)	P value
Time x Column Factor	0.2341	200	0.001171	F (5.287, 50.23) = 1.617	P=0.1694
Time	8.869	100	0.08869	F (2.644, 50.23) = 122.5	P<0.0001
Column Factor	0.2551	2	0.1276	F (2, 19) = 1.435	P=0.2628
Subject	1.689	19	0.08889	F (19, 1900) = 122.8	P<0.0001
Residual	1.375	1900	0.000724		

**SUPP FIG 7B**	
Data summary	
Number of columns (Column Factor)	3
Number of rows (Time)	101
Number of subjects (Subject)	22
Number of missing values	0
Within each row, compare columns (simple effects within rows)	
Number of families	101
Number of comparisons per family	2
Alpha	0.05

**SUPP FIG 7B**					
Dunnett's multiple comparisons test	Mean diff.	95.00% CI of diff.	Below threshold?	Summary	Adjusted P Value
Row 1					
WT vs. +α-syn^{WT}	0.0003457	-0.02410 to 0.02479	No	ns	0.9991
WT vs. +α-syn^{AAA}	-0.004477	-0.03223 to 0.02328	No	ns	0.8888
Row 2					
WT vs. +α-syn^{WT}	-0.00869	-0.02199 to 0.004610	No	ns	0.2167
WT vs. +α-syn^{AAA}	-0.009361	-0.03372 to 0.01499	No	ns	0.5203
Row 3					
WT vs. +α-syn^{WT}	0.002484	-0.01463 to 0.01960	No	ns	0.9087
WT vs. +α-syn^{AAA}	-0.003404	-0.02195 to 0.01514	No	ns	0.8593

Row 4					
WT vs. +α-syn^{WT}	-0.004996	-0.01751 to 0.007518	No	ns	0.5248
WT vs. +α-syn^{AAA}	-0.00241	-0.01515 to 0.01033	No	ns	0.8512
Row 5					
WT vs. +α-syn^{WT}	0.0006336	-0.02385 to 0.02512	No	ns	0.9965
WT vs. +α-syn^{AAA}	0.0008033	-0.02515 to 0.02675	No	ns	0.9954
Row 6					
WT vs. +α-syn^{WT}	-0.005811	-0.01688 to 0.005256	No	ns	0.3506
WT vs. +α-syn^{AAA}	-0.004354	-0.01587 to 0.007163	No	ns	0.551
Row 7					
WT vs. +α-syn^{WT}	-0.006009	-0.01939 to 0.007366	No	ns	0.4027
WT vs. +α-syn^{AAA}	-0.004083	-0.01853 to 0.01036	No	ns	0.7009
Row 8					
WT vs. +α-syn^{WT}	0.007879	-0.006274 to 0.02203	No	ns	0.3045
WT vs. +α-syn^{AAA}	0.005029	-0.009764 to 0.01982	No	ns	0.6104
Row 9					
WT vs. +α-syn^{WT}	-7.25E-05	-0.009925 to 0.009780	No	ns	0.9998
WT vs. +α-syn^{AAA}	-0.004563	-0.01608 to 0.006956	No	ns	0.5226
Row 10					
WT vs. +α-syn^{WT}	0.004015	-0.009366 to 0.01740	No	ns	0.6831
WT vs. +α-syn^{AAA}	0.007972	-0.005085 to 0.02103	No	ns	0.2545
Row 11					
WT vs. +α-syn^{WT}	0.007231	-0.002227 to 0.01669	No	ns	0.1366
WT vs. +α-syn^{AAA}	-0.003287	-0.01470 to 0.008124	No	ns	0.6771
Row 12					
WT vs. +α-syn^{WT}	0.008574	-0.01146 to 0.02861	No	ns	0.4789
WT vs. +α-syn^{AAA}	-0.01664	-0.04045 to 0.007171	No	ns	0.1799
Row 13					
WT vs. +α-syn^{WT}	0.01178	-0.01804 to 0.04160	No	ns	0.5195
WT vs. +α-syn^{AAA}	-0.01711	-0.05361 to 0.01940	No	ns	0.4188
Row 14					
WT vs. +α-syn^{WT}	0.02908	-0.01098 to 0.06914	No	ns	0.1557
WT vs. +α-syn^{AAA}	-0.01307	-0.06513 to 0.03899	No	ns	0.7573
Row 15					
WT vs. +α-syn^{WT}	0.0377	0.001882 to 0.07353	Yes	*	0.0401
WT vs. +α-syn^{AAA}	-0.0159	-0.07991 to 0.04811	No	ns	0.75
Row 16					

WT vs. +α-syn^{WT}	0.03857	-0.004444 to 0.08158	No	ns	0.0787
WT vs. +α-syn^{AAA}	-0.01804	-0.07981 to 0.04373	No	ns	0.6844
Row 17					
WT vs. +α-syn^{WT}	0.03696	-0.02171 to 0.09564	No	ns	0.2314
WT vs. +α-syn^{AAA}	-0.01303	-0.08970 to 0.06364	No	ns	0.8766
Row 18					
WT vs. +α-syn^{WT}	0.05598	-0.001835 to 0.1138	No	ns	0.0574
WT vs. +α-syn^{AAA}	-0.01428	-0.08679 to 0.05822	No	ns	0.8397
Row 19					
WT vs. +α-syn^{WT}	0.05192	-0.009370 to 0.1132	No	ns	0.0971
WT vs. +α-syn^{AAA}	-0.01679	-0.1007 to 0.06710	No	ns	0.8327
Row 20					
WT vs. +α-syn^{WT}	0.05683	-0.002757 to 0.1164	No	ns	0.0611
WT vs. +α-syn^{AAA}	-0.0009646	-0.07722 to 0.07529	No	ns	0.9993
Row 21					
WT vs. +α-syn^{WT}	0.05062	-0.02224 to 0.1235	No	ns	0.1746
WT vs. +α-syn^{AAA}	-0.01359	-0.09979 to 0.07261	No	ns	0.8933
Row 22					
WT vs. +α-syn^{WT}	0.06695	-0.001970 to 0.1359	No	ns	0.0564
WT vs. +α-syn^{AAA}	0.002564	-0.07872 to 0.08385	No	ns	0.9954
Row 23					
WT vs. +α-syn^{WT}	0.06059	-0.01562 to 0.1368	No	ns	0.118
WT vs. +α-syn^{AAA}	0.01017	-0.07340 to 0.09374	No	ns	0.9339
Row 24					
WT vs. +α-syn^{WT}	0.05585	-0.01575 to 0.1274	No	ns	0.1261
WT vs. +α-syn^{AAA}	-0.004438	-0.09330 to 0.08442	No	ns	0.9885
Row 25					
WT vs. +α-syn^{WT}	0.05661	-0.01723 to 0.1304	No	ns	0.1328
WT vs. +α-syn^{AAA}	-0.007656	-0.09977 to 0.08446	No	ns	0.9686
Row 26					
WT vs. +α-syn^{WT}	0.05486	-0.01866 to 0.1284	No	ns	0.1414
WT vs. +α-syn^{AAA}	-0.003728	-0.1012 to 0.09371	No	ns	0.9932
Row 27					
WT vs. +α-syn^{WT}	0.06097	-0.006371 to 0.1283	No	ns	0.0743
WT vs. +α-syn^{AAA}	-0.003441	-0.09452 to 0.08764	No	ns	0.9933
Row 28					
WT vs. +α-syn^{WT}	0.0548	-0.008874 to 0.1185	No	ns	0.09

WT vs. +α-syn^{AAA}	-0.01041	-0.09946 to 0.07863	No	ns	0.9382
Row 29					
WT vs. +α-syn^{WT}	0.05773	-0.007533 to 0.1230	No	ns	0.081
WT vs. +α-syn^{AAA}	0.00612	-0.08479 to 0.09703	No	ns	0.979
Row 30					
WT vs. +α-syn^{WT}	0.05664	-0.004067 to 0.1173	No	ns	0.0666
WT vs. +α-syn^{AAA}	-0.0009433	-0.08577 to 0.08388	No	ns	0.9994
Row 31					
WT vs. +α-syn^{WT}	0.04467	-0.002619 to 0.09195	No	ns	0.0639
WT vs. +α-syn^{AAA}	-0.0135	-0.08983 to 0.06283	No	ns	0.8607
Row 32					
WT vs. +α-syn^{WT}	0.04339	-0.01170 to 0.09848	No	ns	0.1237
WT vs. +α-syn^{AAA}	-0.009742	-0.08104 to 0.06156	No	ns	0.9177
Row 33					
WT vs. +α-syn^{WT}	0.03748	-0.009583 to 0.08454	No	ns	0.121
WT vs. +α-syn^{AAA}	-0.01716	-0.09900 to 0.06468	No	ns	0.8099
Row 34					
WT vs. +α-syn^{WT}	0.04091	-0.01160 to 0.09341	No	ns	0.1267
WT vs. +α-syn^{AAA}	-0.005507	-0.07529 to 0.06428	No	ns	0.9714
Row 35					
WT vs. +α-syn^{WT}	0.0287	-0.02669 to 0.08410	No	ns	0.3511
WT vs. +α-syn^{AAA}	-0.01081	-0.08667 to 0.06505	No	ns	0.9099
Row 36					
WT vs. +α-syn^{WT}	0.04615	-0.006636 to 0.09893	No	ns	0.0865
WT vs. +α-syn^{AAA}	-0.006865	-0.06893 to 0.05520	No	ns	0.9453
Row 37					
WT vs. +α-syn^{WT}	0.0373	-0.01020 to 0.08479	No	ns	0.1281
WT vs. +α-syn^{AAA}	-0.007158	-0.08123 to 0.06692	No	ns	0.9547
Row 38					
WT vs. +α-syn^{WT}	0.03381	-0.01132 to 0.07894	No	ns	0.1475
WT vs. +α-syn^{AAA}	-0.01455	-0.08220 to 0.05311	No	ns	0.8065
Row 39					
WT vs. +α-syn^{WT}	0.03803	-0.008649 to 0.08470	No	ns	0.113
WT vs. +α-syn^{AAA}	-0.009408	-0.07486 to 0.05605	No	ns	0.9061
Row 40					
WT vs. +α-syn^{WT}	0.01999	-0.01626 to 0.05624	No	ns	0.3197
WT vs. +α-syn^{AAA}	-0.01529	-0.07404 to 0.04346	No	ns	0.7247

Row 41					
WT vs. +α-syn^{WT}	0.03937	-0.005949 to 0.08468	No	ns	0.0887
WT vs. +α-syn^{AAA}	0.002481	-0.05795 to 0.06291	No	ns	0.9921
Row 42					
WT vs. +α-syn^{WT}	0.03165	-0.01043 to 0.07374	No	ns	0.1447
WT vs. +α-syn^{AAA}	-0.003585	-0.05913 to 0.05196	No	ns	0.9806
Row 43					
WT vs. +α-syn^{WT}	0.02605	-0.01726 to 0.06936	No	ns	0.2611
WT vs. +α-syn^{AAA}	-0.006905	-0.07067 to 0.05686	No	ns	0.9456
Row 44					
WT vs. +α-syn^{WT}	0.03438	-0.002426 to 0.07119	No	ns	0.0672
WT vs. +α-syn^{AAA}	-0.005127	-0.06395 to 0.05370	No	ns	0.9627
Row 45					
WT vs. +α-syn^{WT}	0.02719	-0.01386 to 0.06824	No	ns	0.2065
WT vs. +α-syn^{AAA}	-0.0178	-0.08974 to 0.05414	No	ns	0.75
Row 46					
WT vs. +α-syn^{WT}	0.02396	-0.01538 to 0.06331	No	ns	0.2586
WT vs. +α-syn^{AAA}	-0.009021	-0.06670 to 0.04866	No	ns	0.8892
Row 47					
WT vs. +α-syn^{WT}	0.02311	-0.01264 to 0.05886	No	ns	0.2241
WT vs. +α-syn^{AAA}	-0.01864	-0.07789 to 0.04061	No	ns	0.629
Row 48					
WT vs. +α-syn^{WT}	0.01855	-0.01386 to 0.05096	No	ns	0.2966
WT vs. +α-syn^{AAA}	-0.01231	-0.07255 to 0.04794	No	ns	0.8123
Row 49					
WT vs. +α-syn^{WT}	0.02622	-0.006235 to 0.05867	No	ns	0.1163
WT vs. +α-syn^{AAA}	-0.01203	-0.06341 to 0.03935	No	ns	0.7722
Row 50					
WT vs. +α-syn^{WT}	0.02914	-0.01136 to 0.06963	No	ns	0.1679
WT vs. +α-syn^{AAA}	-0.007371	-0.06162 to 0.04688	No	ns	0.9164
Row 51					
WT vs. +α-syn^{WT}	0.0169	-0.02048 to 0.05428	No	ns	0.4469
WT vs. +α-syn^{AAA}	-0.02127	-0.07714 to 0.03460	No	ns	0.5296
Row 52					
WT vs. +α-syn^{WT}	0.01465	-0.02437 to 0.05366	No	ns	0.5601
WT vs. +α-syn^{AAA}	-0.01138	-0.07627 to 0.05351	No	ns	0.8609

Row 53					
WT vs. +α-syn^{WT}	0.01568	-0.02001 to 0.05136	No	ns	0.4622
WT vs. +α-syn^{AAA}	-0.01404	-0.07616 to 0.04808	No	ns	0.783
Row 54					
WT vs. +α-syn^{WT}	0.01333	-0.02327 to 0.04993	No	ns	0.5787
WT vs. +α-syn^{AAA}	-0.007587	-0.06476 to 0.04959	No	ns	0.9173
Row 55					
WT vs. +α-syn^{WT}	0.01428	-0.02057 to 0.04913	No	ns	0.4956
WT vs. +α-syn^{AAA}	-0.01013	-0.06374 to 0.04349	No	ns	0.8466
Row 56					
WT vs. +α-syn^{WT}	0.01302	-0.01947 to 0.04551	No	ns	0.5205
WT vs. +α-syn^{AAA}	-0.01503	-0.07477 to 0.04471	No	ns	0.7382
Row 57					
WT vs. +α-syn^{WT}	0.0084	-0.03173 to 0.04853	No	ns	0.8183
WT vs. +α-syn^{AAA}	-0.01292	-0.06852 to 0.04269	No	ns	0.7841
Row 58					
WT vs. +α-syn^{WT}	0.003759	-0.02796 to 0.03547	No	ns	0.9383
WT vs. +α-syn^{AAA}	-0.01797	-0.07264 to 0.03670	No	ns	0.6121
Row 59					
WT vs. +α-syn^{WT}	0.01128	-0.01780 to 0.04036	No	ns	0.5405
WT vs. +α-syn^{AAA}	-0.004663	-0.06095 to 0.05163	No	ns	0.9638
Row 60					
WT vs. +α-syn^{WT}	0.01166	-0.01807 to 0.04140	No	ns	0.5344
WT vs. +α-syn^{AAA}	-0.007698	-0.05771 to 0.04231	No	ns	0.8897
Row 61					
WT vs. +α-syn^{WT}	0.01062	-0.02059 to 0.04182	No	ns	0.6177
WT vs. +α-syn^{AAA}	-0.01404	-0.06964 to 0.04157	No	ns	0.7358
Row 62					
WT vs. +α-syn^{WT}	0.008739	-0.02022 to 0.03770	No	ns	0.6804
WT vs. +α-syn^{AAA}	-0.005429	-0.06482 to 0.05396	No	ns	0.9567
Row 63					
WT vs. +α-syn^{WT}	0.008775	-0.02517 to 0.04272	No	ns	0.7469
WT vs. +α-syn^{AAA}	-0.01217	-0.07002 to 0.04569	No	ns	0.7995
Row 64					
WT vs. +α-syn^{WT}	0.008108	-0.02118 to 0.03739	No	ns	0.7204
WT vs. +α-syn^{AAA}	-0.01242	-0.07148 to 0.04664	No	ns	0.7986
Row 65					

WT vs. +α-syn^{WT}	0.005227	-0.02736 to 0.03781	No	ns	0.8897
WT vs. +α-syn^{AAA}	-0.007065	-0.06217 to 0.04804	No	ns	0.9224
Row 66					
WT vs. +α-syn^{WT}	0.002809	-0.02985 to 0.03546	No	ns	0.9666
WT vs. +α-syn^{AAA}	-0.007511	-0.06797 to 0.05295	No	ns	0.9252
Row 67					
WT vs. +α-syn^{WT}	0.0004811	-0.02699 to 0.02796	No	ns	0.9986
WT vs. +α-syn^{AAA}	-0.008195	-0.06479 to 0.04840	No	ns	0.894
Row 68					
WT vs. +α-syn^{WT}	-0.001987	-0.04003 to 0.03605	No	ns	0.9875
WT vs. +α-syn^{AAA}	-0.01707	-0.07382 to 0.03968	No	ns	0.6664
Row 69					
WT vs. +α-syn^{WT}	-0.004345	-0.03570 to 0.02701	No	ns	0.9168
WT vs. +α-syn^{AAA}	-0.01642	-0.08206 to 0.04923	No	ns	0.7361
Row 70					
WT vs. +α-syn^{WT}	-0.001614	-0.03311 to 0.02988	No	ns	0.9881
WT vs. +α-syn^{AAA}	-0.008189	-0.06431 to 0.04794	No	ns	0.8962
Row 71					
WT vs. +α-syn^{WT}	0.004713	-0.02616 to 0.03559	No	ns	0.9013
WT vs. +α-syn^{AAA}	-0.003878	-0.05676 to 0.04900	No	ns	0.9727
Row 72					
WT vs. +α-syn^{WT}	-0.00105	-0.03219 to 0.03009	No	ns	0.9948
WT vs. +α-syn^{AAA}	-0.008281	-0.07388 to 0.05732	No	ns	0.9203
Row 73					
WT vs. +α-syn^{WT}	0.005642	-0.02832 to 0.03961	No	ns	0.8842
WT vs. +α-syn^{AAA}	-0.01043	-0.07015 to 0.04928	No	ns	0.8598
Row 74					
WT vs. +α-syn^{WT}	0.002485	-0.03623 to 0.04120	No	ns	0.981
WT vs. +α-syn^{AAA}	-0.006111	-0.07077 to 0.05854	No	ns	0.9536
Row 75					
WT vs. +α-syn^{WT}	0.0004612	-0.03768 to 0.03860	No	ns	0.9993
WT vs. +α-syn^{AAA}	-0.008065	-0.07660 to 0.06047	No	ns	0.9334
Row 76					
WT vs. +α-syn^{WT}	0.002195	-0.03124 to 0.03563	No	ns	0.9806
WT vs. +α-syn^{AAA}	-0.008092	-0.07775 to 0.06157	No	ns	0.9308
Row 77					
WT vs. +α-syn^{WT}	-0.005697	-0.04283 to 0.03143	No	ns	0.8992

WT vs. +α-syn^{AAA}	-0.009596	-0.07739 to 0.05820	No	ns	0.9
Row 78					
WT vs. +α-syn^{WT}	0.004076	-0.02841 to 0.03656	No	ns	0.9306
WT vs. +α-syn^{AAA}	-0.006488	-0.06957 to 0.05660	No	ns	0.9444
Row 79					
WT vs. +α-syn^{WT}	-0.0001373	-0.03273 to 0.03245	No	ns	>0.9999
WT vs. +α-syn^{AAA}	-0.001579	-0.06283 to 0.05967	No	ns	0.9966
Row 80					
WT vs. +α-syn^{WT}	0.003053	-0.03395 to 0.04005	No	ns	0.9693
WT vs. +α-syn^{AAA}	-0.0153	-0.07656 to 0.04595	No	ns	0.7464
Row 81					
WT vs. +α-syn^{WT}	-0.001619	-0.03505 to 0.03181	No	ns	0.9893
WT vs. +α-syn^{AAA}	-0.007789	-0.06354 to 0.04797	No	ns	0.9084
Row 82					
WT vs. +α-syn^{WT}	0.0004666	-0.03486 to 0.03580	No	ns	0.9992
WT vs. +α-syn^{AAA}	-0.01342	-0.07402 to 0.04717	No	ns	0.7834
Row 83					
WT vs. +α-syn^{WT}	0.006737	-0.02717 to 0.04065	No	ns	0.8405
WT vs. +α-syn^{AAA}	-0.0108	-0.07613 to 0.05452	No	ns	0.8687
Row 84					
WT vs. +α-syn^{WT}	0.004163	-0.02957 to 0.03790	No	ns	0.9332
WT vs. +α-syn^{AAA}	-0.001109	-0.06638 to 0.06416	No	ns	0.9984
Row 85					
WT vs. +α-syn^{WT}	0.01342	-0.01989 to 0.04673	No	ns	0.5148
WT vs. +α-syn^{AAA}	0.005985	-0.06185 to 0.07382	No	ns	0.9583
Row 86					
WT vs. +α-syn^{WT}	0.002817	-0.02886 to 0.03449	No	ns	0.9646
WT vs. +α-syn^{AAA}	0.0003339	-0.05466 to 0.05533	No	ns	0.9998
Row 87					
WT vs. +α-syn^{WT}	0.001282	-0.03971 to 0.04227	No	ns	0.9954
WT vs. +α-syn^{AAA}	-0.009096	-0.07227 to 0.05408	No	ns	0.897
Row 88					
WT vs. +α-syn^{WT}	0.01017	-0.02236 to 0.04270	No	ns	0.6425
WT vs. +α-syn^{AAA}	0.004897	-0.05721 to 0.06701	No	ns	0.9651
Row 89					
WT vs. +α-syn^{WT}	0.006001	-0.02867 to 0.04067	No	ns	0.8718
WT vs. +α-syn^{AAA}	-0.002265	-0.06976 to 0.06523	No	ns	0.9937

Row 90					
WT vs. +α-syn^{WT}	0.009246	-0.02570 to 0.04419	No	ns	0.7303
WT vs. +α-syn^{AAA}	0.0005383	-0.05991 to 0.06099	No	ns	0.9996
Row 91					
WT vs. +α-syn^{WT}	-0.003201	-0.03548 to 0.02908	No	ns	0.9552
WT vs. +α-syn^{AAA}	-0.002822	-0.06778 to 0.06214	No	ns	0.9895
Row 92					
WT vs. +α-syn^{WT}	0.008033	-0.03032 to 0.04639	No	ns	0.8199
WT vs. +α-syn^{AAA}	0.006313	-0.05947 to 0.07210	No	ns	0.9515
Row 93					
WT vs. +α-syn^{WT}	0.01092	-0.03153 to 0.05337	No	ns	0.7371
WT vs. +α-syn^{AAA}	0.005657	-0.06550 to 0.07681	No	ns	0.9652
Row 94					
WT vs. +α-syn^{WT}	0.005262	-0.03398 to 0.04451	No	ns	0.9202
WT vs. +α-syn^{AAA}	0.007404	-0.05150 to 0.06631	No	ns	0.9209
Row 95					
WT vs. +α-syn^{WT}	2.99E-05	-0.03816 to 0.03822	No	ns	>0.9999
WT vs. +α-syn^{AAA}	-0.004741	-0.05836 to 0.04888	No	ns	0.9627
Row 96					
WT vs. +α-syn^{WT}	0.001522	-0.03331 to 0.03635	No	ns	0.9913
WT vs. +α-syn^{AAA}	-0.006852	-0.06681 to 0.05310	No	ns	0.9364
Row 97					
WT vs. +α-syn^{WT}	0.00961	-0.02380 to 0.04302	No	ns	0.7012
WT vs. +α-syn^{AAA}	0.005772	-0.06265 to 0.07419	No	ns	0.9621
Row 98					
WT vs. +α-syn^{WT}	0.004905	-0.03101 to 0.04082	No	ns	0.9197
WT vs. +α-syn^{AAA}	0.004678	-0.06189 to 0.07125	No	ns	0.9742
Row 99					
WT vs. +α-syn^{WT}	0.004174	-0.03564 to 0.04399	No	ns	0.9516
WT vs. +α-syn^{AAA}	0.002591	-0.05492 to 0.06010	No	ns	0.9899
Row 100					
WT vs. +α-syn^{WT}	0.009698	-0.02878 to 0.04817	No	ns	0.7579
WT vs. +α-syn^{AAA}	0.00257	-0.05364 to 0.05878	No	ns	0.9893
Row 101					
WT vs. +α-syn^{WT}	0.005589	-0.03416 to 0.04534	No	ns	0.9153
WT vs. +α-syn^{AAA}	0.01048	-0.05365 to 0.07462	No	ns	0.8752

**SUPP FIG 7B**								
Test details	Mean 1	Mean 2	Mean diff.	SE of diff.	N1	N2	q	DF
Row 1								
WT vs. +α-syn^{WT}	0.008841	0.008496	0.0003457	0.009701	7	8	0.03563	11.31
WT vs. +α-syn^{AAA}	0.008841	0.01332	-0.004477	0.0111	7	7	0.4032	12
Row 2								
WT vs. +α-syn^{WT}	0.001161	0.009851	-0.00869	0.005332	7	8	1.63	12.2
WT vs. +α-syn^{AAA}	0.001161	0.01052	-0.009361	0.009279	7	7	1.009	8.738
Row 3								
WT vs. +α-syn^{WT}	0.01131	0.008822	0.002484	0.006854	7	8	0.3625	12.07
WT vs. +α-syn^{AAA}	0.01131	0.01471	-0.003404	0.007419	7	7	0.4588	11.99
Row 4								
WT vs. +α-syn^{WT}	0.002611	0.007607	-0.004996	0.005056	7	8	0.9882	13
WT vs. +α-syn^{AAA}	0.002611	0.005021	-0.00241	0.005091	7	7	0.4734	11.92
Row 5								
WT vs. +α-syn^{WT}	0.00883	0.008196	0.0006336	0.009274	7	8	0.06832	8.472
WT vs. +α-syn^{AAA}	0.00883	0.008027	0.0008033	0.0102	7	7	0.07873	10.59
Row 6								
WT vs. +α-syn^{WT}	0.003082	0.008893	-0.005811	0.004469	7	8	1.3	12.94
WT vs. +α-syn^{AAA}	0.003082	0.007436	-0.004354	0.004586	7	7	0.9494	11.58
Row 7								
WT vs. +α-syn^{WT}	-0.004874	0.001136	-0.006009	0.00485	7	8	1.239	6.899
WT vs. +α-syn^{AAA}	-0.004874	-0.0007909	-0.004083	0.005686	7	7	0.718	10.67
Row 8								
WT vs. +α-syn^{WT}	0.002227	-0.005652	0.007879	0.005577	7	8	1.413	10.74
WT vs. +α-syn^{AAA}	0.002227	-0.002802	0.005029	0.005872	7	7	0.8565	11.32
Row 9								
WT vs. +α-syn^{WT}	-0.002071	-0.001999	-7.25E-05	0.00397	7	8	0.01826	12.72
WT vs. +α-syn^{AAA}	-0.002071	0.002492	-0.004563	0.004585	7	7	0.9952	11.55
Row 10								
WT vs. +α-syn^{WT}	0.001637	-0.002378	0.004015	0.005406	7	8	0.7427	13
WT vs. +α-syn^{AAA}	0.001637	-0.006335	0.007972	0.005224	7	7	1.526	12
Row 11								
WT vs. +α-syn^{WT}	0.00312	-0.004111	0.007231	0.003739	7	8	1.934	10.99
WT vs. +α-syn^{AAA}	0.00312	0.006408	-0.003287	0.004334	7	7	0.7586	8.589
Row 12								
WT vs. +α-syn^{WT}	0.02891	0.02033	0.008574	0.008046	7	8	1.066	12.35
WT vs. +α-syn^{AAA}	0.02891	0.04554	-0.01664	0.009489	7	7	1.753	11.66

Row 13								
WT vs. +α-syn^{WT}	0.05508	0.0433	0.01178	0.01175	7	8	1.003	10.73
WT vs. +α-syn^{AAA}	0.05508	0.07218	-0.01711	0.01458	7	7	1.173	11.87
Row 14								
WT vs. +α-syn^{WT}	0.08741	0.05833	0.02908	0.01547	7	8	1.879	9.449
WT vs. +α-syn^{AAA}	0.08741	0.1005	-0.01307	0.02077	7	7	0.6292	11.74
Row 15								
WT vs. +α-syn^{WT}	0.1034	0.06567	0.0377	0.0138	7	8	2.733	9.299
WT vs. +α-syn^{AAA}	0.1034	0.1193	-0.0159	0.02473	7	7	0.6429	9.47
Row 16								
WT vs. +α-syn^{WT}	0.1209	0.08238	0.03857	0.01716	7	8	2.247	11.76
WT vs. +α-syn^{AAA}	0.1209	0.139	-0.01804	0.02426	7	7	0.7437	10.51
Row 17								
WT vs. +α-syn^{WT}	0.138	0.1011	0.03696	0.02306	7	8	1.603	10.55
WT vs. +α-syn^{AAA}	0.138	0.1511	-0.01303	0.03052	7	7	0.4269	11.54
Row 18								
WT vs. +α-syn^{WT}	0.1571	0.1012	0.05598	0.02274	7	8	2.461	10.63
WT vs. +α-syn^{AAA}	0.1571	0.1714	-0.01428	0.02893	7	7	0.4937	11.77
Row 19								
WT vs. +α-syn^{WT}	0.1653	0.1134	0.05192	0.02433	7	8	2.134	11.32
WT vs. +α-syn^{AAA}	0.1653	0.1821	-0.01679	0.03318	7	7	0.5061	11.03
Row 20								
WT vs. +α-syn^{WT}	0.1846	0.1278	0.05683	0.02339	7	8	2.429	10.47
WT vs. +α-syn^{AAA}	0.1846	0.1856	-0.0009646	0.0304	7	7	0.03173	11.69
Row 21								
WT vs. +α-syn^{WT}	0.1826	0.132	0.05062	0.02794	7	8	1.812	9.06
WT vs. +α-syn^{AAA}	0.1826	0.1962	-0.01359	0.03448	7	7	0.3942	11.98
Row 22								
WT vs. +α-syn^{WT}	0.2004	0.1334	0.06695	0.02664	7	8	2.513	9.492
WT vs. +α-syn^{AAA}	0.2004	0.1978	0.002564	0.03251	7	7	0.07885	11.99
Row 23								
WT vs. +α-syn^{WT}	0.2088	0.1482	0.06059	0.02953	7	8	2.052	9.635
WT vs. +α-syn^{AAA}	0.2088	0.1987	0.01017	0.03328	7	7	0.3056	11.58
Row 24								
WT vs. +α-syn^{WT}	0.2062	0.1504	0.05585	0.02786	7	8	2.004	9.897
WT vs. +α-syn^{AAA}	0.2062	0.2107	-0.004438	0.03551	7	7	0.125	11.91

Row 25								
WT vs. +α-syn^{WT}	0.2165	0.1599	0.05661	0.02866	7	8	1.975	9.75
WT vs. +α-syn^{AAA}	0.2165	0.2242	-0.007656	0.03681	7	7	0.208	11.9
Row 26								
WT vs. +α-syn^{WT}	0.2173	0.1624	0.05486	0.02801	7	8	1.959	8.735
WT vs. +α-syn^{AAA}	0.2173	0.221	-0.003728	0.03886	7	7	0.09595	11.71
Row 27								
WT vs. +α-syn^{WT}	0.2144	0.1534	0.06097	0.02593	7	8	2.351	9.279
WT vs. +α-syn^{AAA}	0.2144	0.2178	-0.003441	0.03624	7	7	0.09495	11.52
Row 28								
WT vs. +α-syn^{WT}	0.1991	0.1443	0.0548	0.02469	7	8	2.22	9.67
WT vs. +α-syn^{AAA}	0.1991	0.2095	-0.01041	0.03531	7	7	0.2949	11.23
Row 29								
WT vs. +α-syn^{WT}	0.1934	0.1356	0.05773	0.02506	7	8	2.303	9.139
WT vs. +α-syn^{AAA}	0.1934	0.1873	0.00612	0.0361	7	7	0.1696	11.33
Row 30								
WT vs. +α-syn^{WT}	0.1884	0.1317	0.05664	0.02374	7	8	2.386	10.21
WT vs. +α-syn^{AAA}	0.1884	0.1893	-0.0009433	0.0336	7	7	0.02808	11.13
Row 31								
WT vs. +α-syn^{WT}	0.1719	0.1272	0.04467	0.01894	7	8	2.359	12.1
WT vs. +α-syn^{AAA}	0.1719	0.1854	-0.0135	0.02953	7	7	0.4572	9.534
Row 32								
WT vs. +α-syn^{WT}	0.1677	0.1243	0.04339	0.0217	7	8	2	10.71
WT vs. +α-syn^{AAA}	0.1677	0.1774	-0.009742	0.02839	7	7	0.3431	11.58
Row 33								
WT vs. +α-syn^{WT}	0.1597	0.1223	0.03748	0.01879	7	8	1.994	11.84
WT vs. +α-syn^{AAA}	0.1597	0.1769	-0.01716	0.03143	7	7	0.5459	9.147
Row 34								
WT vs. +α-syn^{WT}	0.1518	0.1109	0.04091	0.02046	7	8	2	9.966
WT vs. +α-syn^{AAA}	0.1518	0.1573	-0.005507	0.02777	7	7	0.1983	11.54
Row 35								
WT vs. +α-syn^{WT}	0.1431	0.1144	0.0287	0.02194	7	8	1.308	11.12
WT vs. +α-syn^{AAA}	0.1431	0.1539	-0.01081	0.03002	7	7	0.3601	11.08
Row 36								
WT vs. +α-syn^{WT}	0.1462	0.1001	0.04615	0.02099	7	8	2.199	11.46
WT vs. +α-syn^{AAA}	0.1462	0.1531	-0.006865	0.02481	7	7	0.2767	11.93
Row 37								

WT vs. +α-syn^{WT}	0.1401	0.1028	0.0373	0.01917	7	8	1.945	12.9
WT vs. +α-syn^{AAA}	0.1401	0.1473	-0.007158	0.02851	7	7	0.2511	9.261
Row 38								
WT vs. +α-syn^{WT}	0.1375	0.1037	0.03381	0.01806	7	8	1.872	12.05
WT vs. +α-syn^{AAA}	0.1375	0.1521	-0.01455	0.0264	7	7	0.5509	10.08
Row 39								
WT vs. +α-syn^{WT}	0.132	0.09395	0.03803	0.01883	7	8	2.02	12.82
WT vs. +α-syn^{AAA}	0.132	0.1414	-0.009408	0.02555	7	7	0.3682	10.09
Row 40								
WT vs. +α-syn^{WT}	0.1207	0.1007	0.01999	0.01464	7	8	1.365	12.99
WT vs. +α-syn^{AAA}	0.1207	0.1359	-0.01529	0.02236	7	7	0.6839	8.682
Row 41								
WT vs. +α-syn^{WT}	0.1277	0.08837	0.03937	0.01803	7	8	2.183	11.52
WT vs. +α-syn^{AAA}	0.1277	0.1253	0.002481	0.02394	7	7	0.1036	11.16
Row 42								
WT vs. +α-syn^{WT}	0.119	0.08738	0.03165	0.01674	7	8	1.891	11.5
WT vs. +α-syn^{AAA}	0.119	0.1226	-0.003585	0.02203	7	7	0.1627	11.25
Row 43								
WT vs. +α-syn^{WT}	0.1126	0.08656	0.02605	0.01721	7	8	1.514	11.4
WT vs. +α-syn^{AAA}	0.1126	0.1195	-0.006905	0.02502	7	7	0.276	10.44
Row 44								
WT vs. +α-syn^{WT}	0.1119	0.07754	0.03438	0.01486	7	8	2.313	12.94
WT vs. +α-syn^{AAA}	0.1119	0.1171	-0.005127	0.02256	7	7	0.2273	9.061
Row 45								
WT vs. +α-syn^{WT}	0.1061	0.07886	0.02719	0.01628	7	8	1.67	11.25
WT vs. +α-syn^{AAA}	0.1061	0.1239	-0.0178	0.02769	7	7	0.6431	9.253
Row 46								
WT vs. +α-syn^{WT}	0.1031	0.07909	0.02396	0.01587	7	8	1.51	12.85
WT vs. +α-syn^{AAA}	0.1031	0.1121	-0.009021	0.02239	7	7	0.4029	9.742
Row 47								
WT vs. +α-syn^{WT}	0.09653	0.07342	0.02311	0.01441	7	8	1.604	12.73
WT vs. +α-syn^{AAA}	0.09653	0.1152	-0.01864	0.02233	7	7	0.8349	8.24
Row 48								
WT vs. +α-syn^{WT}	0.09373	0.07518	0.01855	0.01309	7	8	1.417	12.99
WT vs. +α-syn^{AAA}	0.09373	0.106	-0.01231	0.02267	7	7	0.5428	8.192
Row 49								
WT vs. +α-syn^{WT}	0.09438	0.06816	0.02622	0.01308	7	8	2.004	12.74

WT vs. +α-syn^{AAA}	0.09438	0.1064	-0.01203	0.0198	7	7	0.6077	9.326
Row 50								
WT vs. +α-syn^{WT}	0.09121	0.06207	0.02914	0.01633	7	8	1.784	12.79
WT vs. +α-syn^{AAA}	0.09121	0.09858	-0.007371	0.02129	7	7	0.3461	10.47
Row 51								
WT vs. +α-syn^{WT}	0.0835	0.0666	0.0169	0.0151	7	8	1.119	12.98
WT vs. +α-syn^{AAA}	0.0835	0.1048	-0.02127	0.02145	7	7	0.9918	9.114
Row 52								
WT vs. +α-syn^{WT}	0.08423	0.06958	0.01465	0.01571	7	8	0.9327	12.61
WT vs. +α-syn^{AAA}	0.08423	0.09561	-0.01138	0.02491	7	7	0.4569	9.108
Row 53								
WT vs. +α-syn^{WT}	0.0775	0.06183	0.01568	0.01432	7	8	1.094	12.34
WT vs. +α-syn^{AAA}	0.0775	0.09154	-0.01404	0.02378	7	7	0.5904	8.97
Row 54								
WT vs. +α-syn^{WT}	0.07821	0.06488	0.01333	0.01476	7	8	0.9032	12.82
WT vs. +α-syn^{AAA}	0.07821	0.0858	-0.007587	0.02204	7	7	0.3442	9.345
Row 55								
WT vs. +α-syn^{WT}	0.07235	0.05807	0.01428	0.01368	7	8	1.043	10.49
WT vs. +α-syn^{AAA}	0.07235	0.08248	-0.01013	0.021	7	7	0.4821	10.34
Row 56								
WT vs. +α-syn^{WT}	0.07129	0.05827	0.01302	0.01307	7	8	0.9962	12.56
WT vs. +α-syn^{AAA}	0.07129	0.08633	-0.01503	0.02268	7	7	0.6627	8.581
Row 57								
WT vs. +α-syn^{WT}	0.06356	0.05516	0.0084	0.01583	7	8	0.5307	10.81
WT vs. +α-syn^{AAA}	0.06356	0.07648	-0.01292	0.022	7	7	0.587	11.07
Row 58								
WT vs. +α-syn^{WT}	0.05405	0.05029	0.003759	0.01276	7	8	0.2946	12.54
WT vs. +α-syn^{AAA}	0.05405	0.07202	-0.01797	0.02091	7	7	0.8593	8.939
Row 59								
WT vs. +α-syn^{WT}	0.06284	0.05156	0.01128	0.0117	7	8	0.9639	12.6
WT vs. +α-syn^{AAA}	0.06284	0.06751	-0.004663	0.02084	7	7	0.2237	7.568
Row 60								
WT vs. +α-syn^{WT}	0.05769	0.04603	0.01166	0.01198	7	8	0.9733	12.74
WT vs. +α-syn^{AAA}	0.05769	0.06539	-0.007698	0.01914	7	7	0.4022	8.962
Row 61								
WT vs. +α-syn^{WT}	0.05457	0.04395	0.01062	0.0126	7	8	0.8426	12.95
WT vs. +α-syn^{AAA}	0.05457	0.06861	-0.01404	0.02106	7	7	0.6667	8.456

Row 62								
WT vs. +α-syn^{WT}	0.05403	0.04529	0.008739	0.0117	7	8	0.7469	13
WT vs. +α-syn^{AAA}	0.05403	0.05946	-0.005429	0.02211	7	7	0.2455	7.774
Row 63								
WT vs. +α-syn^{WT}	0.04687	0.03809	0.008775	0.0136	7	8	0.6453	12.12
WT vs. +α-syn^{AAA}	0.04687	0.05903	-0.01217	0.02154	7	7	0.5648	7.763
Row 64								
WT vs. +α-syn^{WT}	0.049	0.04089	0.008108	0.01182	7	8	0.6857	12.94
WT vs. +α-syn^{AAA}	0.049	0.06142	-0.01242	0.02193	7	7	0.5664	7.666
Row 65								
WT vs. +α-syn^{WT}	0.04421	0.03898	0.005227	0.01302	7	8	0.4014	11.9
WT vs. +α-syn^{AAA}	0.04421	0.05127	-0.007065	0.02123	7	7	0.3328	9.301
Row 66								
WT vs. +α-syn^{WT}	0.04845	0.04565	0.002809	0.01309	7	8	0.2145	12.2
WT vs. +α-syn^{AAA}	0.04845	0.05597	-0.007511	0.02301	7	7	0.3265	8.681
Row 67								
WT vs. +α-syn^{WT}	0.04018	0.0397	0.0004811	0.01103	7	8	0.04361	12.36
WT vs. +α-syn^{AAA}	0.04018	0.04837	-0.008195	0.02079	7	7	0.3943	7.288
Row 68								
WT vs. +α-syn^{WT}	0.03058	0.03256	-0.001987	0.01528	7	8	0.1301	12.37
WT vs. +α-syn^{AAA}	0.03058	0.04765	-0.01707	0.02211	7	7	0.7723	9.967
Row 69								
WT vs. +α-syn^{WT}	0.03034	0.03468	-0.004345	0.0126	7	8	0.3449	12.44
WT vs. +α-syn^{AAA}	0.03034	0.04675	-0.01642	0.02461	7	7	0.667	8.034
Row 70								
WT vs. +α-syn^{WT}	0.03558	0.03719	-0.001614	0.0127	7	8	0.1271	12.81
WT vs. +α-syn^{AAA}	0.03558	0.04377	-0.008189	0.02102	7	7	0.3895	7.996
Row 71								
WT vs. +α-syn^{WT}	0.02954	0.02483	0.004713	0.01248	7	8	0.3778	13
WT vs. +α-syn^{AAA}	0.02954	0.03342	-0.003878	0.02003	7	7	0.1936	8.468
Row 72								
WT vs. +α-syn^{WT}	0.02808	0.02913	-0.00105	0.01255	7	8	0.08366	12.74
WT vs. +α-syn^{AAA}	0.02808	0.03636	-0.008281	0.02451	7	7	0.3379	7.897
Row 73								
WT vs. +α-syn^{WT}	0.02985	0.02421	0.005642	0.0137	7	8	0.412	12.78
WT vs. +α-syn^{AAA}	0.02985	0.04029	-0.01043	0.02272	7	7	0.4592	8.686

Row 74								
WT vs. +α-syn^{WT}	0.02438	0.0219	0.002485	0.01545	7	8	0.1608	11.79
WT vs. +α-syn^{AAA}	0.02438	0.03049	-0.006111	0.02401	7	7	0.2545	7.679
Row 75								
WT vs. +α-syn^{WT}	0.02355	0.02309	0.0004612	0.01517	7	8	0.0304	11.48
WT vs. +α-syn^{AAA}	0.02355	0.03162	-0.008065	0.02627	7	7	0.307	9.046
Row 76								
WT vs. +α-syn^{WT}	0.02282	0.02062	0.002195	0.0135	7	8	0.1625	12.96
WT vs. +α-syn^{AAA}	0.02282	0.03091	-0.008092	0.02581	7	7	0.3135	7.591
Row 77								
WT vs. +α-syn^{WT}	0.02015	0.02585	-0.005697	0.0149	7	8	0.3823	12.32
WT vs. +α-syn^{AAA}	0.02015	0.02975	-0.009596	0.02514	7	7	0.3817	7.619
Row 78								
WT vs. +α-syn^{WT}	0.01994	0.01587	0.004076	0.01301	7	8	0.3133	12.11
WT vs. +α-syn^{AAA}	0.01994	0.02643	-0.006488	0.02321	7	7	0.2795	7.351
Row 79								
WT vs. +α-syn^{WT}	0.01698	0.01712	-0.0001373	0.01312	7	8	0.01046	12.63
WT vs. +α-syn^{AAA}	0.01698	0.01856	-0.001579	0.02318	7	7	0.0681	8.44
Row 80								
WT vs. +α-syn^{WT}	0.01445	0.0114	0.003053	0.01485	7	8	0.2056	12.3
WT vs. +α-syn^{AAA}	0.01445	0.02976	-0.0153	0.02359	7	7	0.6488	9.289
Row 81								
WT vs. +α-syn^{WT}	0.01279	0.01441	-0.001619	0.01344	7	8	0.1205	12.47
WT vs. +α-syn^{AAA}	0.01279	0.02058	-0.007789	0.02142	7	7	0.3636	9.166
Row 82								
WT vs. +α-syn^{WT}	0.0116	0.01113	0.0004666	0.01423	7	8	0.0328	12.64
WT vs. +α-syn^{AAA}	0.0116	0.02502	-0.01342	0.02271	7	7	0.591	8.027
Row 83								
WT vs. +α-syn^{WT}	0.01124	0.004504	0.006737	0.01369	7	8	0.492	12.95
WT vs. +α-syn^{AAA}	0.01124	0.02204	-0.0108	0.02437	7	7	0.4432	7.849
Row 84								
WT vs. +α-syn^{WT}	0.01169	0.007524	0.004163	0.01356	7	8	0.3071	12.45
WT vs. +α-syn^{AAA}	0.01169	0.0128	-0.001109	0.02412	7	7	0.04599	7.498
Row 85								
WT vs. +α-syn^{WT}	0.01474	0.001318	0.01342	0.01333	7	8	1.007	12.05
WT vs. +α-syn^{AAA}	0.01474	0.008756	0.005985	0.02485	7	7	0.2408	7.211
Row 86								

WT vs. +α-syn^{WT}	0.00397	0.001153	0.002817	0.01274	7	8	0.2211	12.51
WT vs. +α-syn^{AAA}	0.00397	0.003636	0.0003339	0.02055	7	7	0.01625	7.896
Row 87								
WT vs. +α-syn^{WT}	0.005935	0.004653	0.001282	0.01623	7	8	0.079	11.12
WT vs. +α-syn^{AAA}	0.005935	0.01503	-0.009096	0.02345	7	7	0.388	7.651
Row 88								
WT vs. +α-syn^{WT}	0.007949	-0.002218	0.01017	0.01254	7	8	0.8107	9.349
WT vs. +α-syn^{AAA}	0.007949	0.003052	0.004897	0.02228	7	7	0.2198	6.597
Row 89								
WT vs. +α-syn^{WT}	0.004116	-0.001886	0.006001	0.01377	7	8	0.436	11.33
WT vs. +α-syn^{AAA}	0.004116	0.006381	-0.002265	0.02464	7	7	0.09193	7.097
Row 90								
WT vs. +α-syn^{WT}	0.004733	-0.004512	0.009246	0.01375	7	8	0.6725	10.63
WT vs. +α-syn^{AAA}	0.004733	0.004195	0.0005383	0.0221	7	7	0.02436	7.141
Row 91								
WT vs. +α-syn^{WT}	-0.002296	0.0009054	-0.003201	0.01282	7	8	0.2496	11.38
WT vs. +α-syn^{AAA}	-0.002296	0.0005266	-0.002822	0.02367	7	7	0.1192	7.04
Row 92								
WT vs. +α-syn^{WT}	0.0001063	-0.007927	0.008033	0.01522	7	8	0.5278	11.29
WT vs. +α-syn^{AAA}	0.0001063	-0.006207	0.006313	0.02424	7	7	0.2605	7.397
Row 93								
WT vs. +α-syn^{WT}	-0.005161	-0.01608	0.01092	0.01646	7	8	0.663	9.693
WT vs. +α-syn^{AAA}	-0.005161	-0.01082	0.005657	0.02579	7	7	0.2193	6.89
Row 94								
WT vs. +α-syn^{WT}	-0.006417	-0.01168	0.005262	0.01559	7	8	0.3374	11.4
WT vs. +α-syn^{AAA}	-0.006417	-0.01382	0.007404	0.02199	7	7	0.3366	7.876
Row 95								
WT vs. +α-syn^{WT}	-0.01135	-0.01138	2.99E-05	0.01542	7	8	0.001941	12.92
WT vs. +α-syn^{AAA}	-0.01135	-0.006609	-0.004741	0.02088	7	7	0.227	9.948
Row 96								
WT vs. +α-syn^{WT}	-0.01296	-0.01448	0.001522	0.01405	7	8	0.1083	12.79
WT vs. +α-syn^{AAA}	-0.01296	-0.006105	-0.006852	0.02287	7	7	0.2996	8.793
Row 97								
WT vs. +α-syn^{WT}	-0.008903	-0.01851	0.00961	0.01343	7	8	0.7156	12.47
WT vs. +α-syn^{AAA}	-0.008903	-0.01468	0.005772	0.02517	7	7	0.2293	7.346
Row 98								
WT vs. +α-syn^{WT}	-0.01359	-0.01849	0.004905	0.0145	7	8	0.3384	12.88

WT vs. +α-syn^{AAA}	-0.01359	-0.01826	0.004678	0.02488	7	7	0.188	7.913
Row 99								
WT vs. +α-syn^{WT}	-0.006884	-0.01106	0.004174	0.01608	7	8	0.2596	12.97
WT vs. +α-syn^{AAA}	-0.006884	-0.009475	0.002591	0.02216	7	7	0.1169	9.313
Row 100								
WT vs. +α-syn^{WT}	-0.009599	-0.0193	0.009698	0.01545	7	8	0.6279	12.34
WT vs. +α-syn^{AAA}	-0.009599	-0.01217	0.00257	0.02132	7	7	0.1205	8.539
Row 101								
WT vs. +α-syn^{WT}	-0.01727	-0.02286	0.005589	0.01605	7	8	0.3482	12.93
WT vs. +α-syn^{AAA}	-0.01727	-0.02775	0.01048	0.02436	7	7	0.4305	8.586

**SUPP FIG 7C**	WT	+α-syn^{WT}	+α-syn^{AAA}
Number of values	7	8	7
Mean	0.2278	0.16617	0.22989
Std. Deviation	0.06514	0.03702	0.07401
Std. Error of Mean	0.02462	0.01309	0.02797
Test for normal distribution			
Shapiro-Wilk test			
W	0.8212	0.968	0.871
P value	0.0659	0.8821	0.1895
Passed normality test (alpha=0.05)?	Yes	Yes	Yes
P value summary	ns	ns	ns

**SUPP FIG 7C**								
Number of families	1							
Number of comparisons per family	2							
Alpha	0.05							
Dunnett's multiple comparisons test	Mean diff.	95.00% CI of diff.	Below threshold?	Summary	Adjusted P Value	A-?		
WT vs. +α-syn^{WT}	0.06163	-0.01219 to 0.1355	No	ns	0.1074	B	+α-syn^{WT}	
WT vs. +α-syn^{AAA}	-0.002094	-0.07834 to 0.07415	No	ns	0.9968	C	+α-syn^{AAA}	
Test details	Mean 1	Mean 2	Mean diff.	SE of diff.	n1	n2	q	DF
WT vs. +α-syn^{WT}	0.2278	0.1662	0.06163	0.03094	7	8	1.992	19
WT vs. +α-syn^{AAA}	0.2278	0.2299	-0.002094	0.03196	7	7	0.06552	19

**SUPP FIG 7D**	WT	+α-syn^{WT}	+α-syn^{AAA}
Number of values	7	8	7
Mean	0.0198	0.013777	0.019613
Std. Deviation	0.0057	0.004354	0.007001
Std. Error of Mean	0.0021	0.00154	0.002646
Test for normal distribution			
Shapiro-Wilk test			
W	0.7881	0.9045	0.8713
P value	0.0312	0.317	0.1906
Passed normality test (alpha=0.05)?	No	Yes	Yes
P value summary	*	ns	ns

**SUPP FIG 7D**	
Kruskal-Wallis test	
P value	0.0955
Exact or approximate P value?	Exact
P value summary	ns
Do the medians vary signif. (P < 0.05)?	No
Number of groups	3
Kruskal-Wallis statistic	4.646
Data summary	
Number of treatments (columns)	3
Number of values (total)	22

**SUPP FIG 7E**	WT	α-syn^{WT}	α-syn^{AAA}
Number of values	7	8	7
Mean	49.7998	51.0951	49.4262
Std. Deviation	8.9036	5.9118	9.7542
Std. Error of Mean	3.3652	2.0901	3.6867
Shapiro-Wilk test			
W	0.9496	0.909	0.9737
P value	0.7264	0.3474	0.924
Passed normality test (alpha=0.05)?	Yes	Yes	Yes
P value summary	ns	ns	ns

**SUPP FIG 7E**								
Number of families	1							
Number of comparisons per family	2							
Alpha	0.05							
Dunnett's multiple comparisons test	Mean diff.	95.00% CI of diff.	Below threshold?	Summary	Adjusted P Value	A-?		
WT vs. α-syn^{WT}	-1.295	-11.47 to 8.883	No	ns	0.9345	B	α-syn^{WT}	
WT vs. α-syn^{AAA}	0.3736	-10.14 to 10.89	No	ns	0.9947	C	α-syn^{AAA}	
Test details	Mean 1	Mean 2	Mean diff.	SE of diff.	n1	n2	q	DF
WT vs. α-syn^{WT}	49.8	51.1	-1.295	4.266	7	8	0.3036	19
WT vs. α-syn^{AAA}	49.8	49.43	0.3736	4.406	7	7	0.08479	19

**SUPP FIG 7F**	WT	α-syn^{WT}	α-syn^{AAA}
Number of values	7	8	7
Mean	38.0904	43.9426	34.99
Std. Deviation	9.2043	12.4139	9.824
Std. Error of Mean	3.4789	4.389	3.7131
Shapiro-Wilk test			
W	0.9171	0.9084	0.9327
P value	0.4469	0.3427	0.5743
Passed normality test (alpha=0.05)?	Yes	Yes	Yes
P value summary	ns	ns	ns

**SUPP FIG 7F**								
Number of families	1							
Number of comparisons per family	2							
Alpha	0.05							
Dunnett's multiple comparisons test	Mean diff.	95.00% CI of diff.	Below threshold?	Summary	Adjusted P Value	A-?		
WT vs. α-syn^{WT}	-5.852	-19.04 to 7.331	No	ns	0.4757	B	α-syn^{WT}	
WT vs. α-syn^{AAA}	3.1	-10.52 to 16.72	No	ns	0.8093	C	α-syn^{AAA}	
Test details	Mean 1	Mean 2	Mean diff.	SE of diff.	n1	n2	q	DF
WT vs. α-syn^{WT}	38.09	43.94	-5.852	5.526	7	8	1.059	19
WT vs. α-syn^{AAA}	38.09	34.99	3.1	5.707	7	7	0.5432	19
